# Metazoan Ribosome Inactivating Protein encoding genes acquired by Horizontal Gene Transfer

**DOI:** 10.1038/s41598-017-01859-1

**Published:** 2017-05-12

**Authors:** Walter J. Lapadula, Paula L. Marcet, María L. Mascotti, M. Virginia Sanchez-Puerta, Maximiliano Juri Ayub

**Affiliations:** 1 Instituto Multidisciplinario de Investigaciones Biológicas de San Luis, IMIBIO-SL-CONICET and Facultad de Química, Bioquímica y Farmacia, Universidad Nacional de San Luis, San Luis, Argentina; 2 Centers for Disease Control and Prevention, Division of Parasitic Diseases and Malaria, Atlanta, USA; 3 IBAM, Universidad Nacional de Cuyo, CONICET, Facultad de Ciencias Agrarias, Almirante Brown 500, M5528AHB Chacras de Coria, Argentina

## Abstract

Ribosome inactivating proteins (RIPs) are RNA *N*-glycosidases that depurinate a specific adenine residue in the conserved sarcin/ricin loop of 28S rRNA. These enzymes are widely distributed among plants and their presence has also been confirmed in several bacterial species. Recently, we reported for the first time *in silico* evidence of RIP encoding genes in metazoans, in two closely related species of insects: *Aedes aegypti* and *Culex quinquefasciatus*. Here, we have experimentally confirmed the presence of these genes in mosquitoes and attempted to unveil their evolutionary history. A detailed study was conducted, including evaluation of taxonomic distribution, phylogenetic inferences and microsynteny analyses, indicating that mosquito RIP genes derived from a single Horizontal Gene Transfer (HGT) event, probably from a cyanobacterial donor species. Moreover, evolutionary analyses show that, after the HGT event, these genes evolved under purifying selection, strongly suggesting they play functional roles in these organisms.

## Introduction

Ribosome inactivating proteins (RIPs, EC 3.2.2.22) irreversibly modify ribosomes through the depurination of an adenine residue in the conserved alpha-sarcin/ricin loop of 28S rRNA^1–4^. This modification prevents the binding of elongation factor 2 to the ribosome, arresting protein synthesis^5, 6^. The occurrence of RIP genes has been experimentally confirmed in a wide range of plant taxa, as well as in several species of Gram positive and Gram negative bacteria^7–9^. Additionally, the exponential increase of information in databases has suggested the presence of genes coding for RIP–domain containing proteins in lineages of Fungi, Cyanobacteria and Metazoa^10–12^. Although several of these toxins have been extensively studied at the biochemical level, their biological roles remain open to speculation. In some cases, it seems reasonable to predict their functions. For instance, the high toxicity of the prototypical RIP ricin supports an antifeedant role, whereas bacterial RIPs shiga and shiga-like toxins are strong virulence factors for their harboring bacteria. Antiviral and other defense activities have been postulated for other plant RIPs, but no concluding evidence has been obtained. Recently, the RIP of the symbiotic *Spiroplasma* (class Mollicutes) in *Drosophila neotestacea* was shown to play a defensive role in preventing a virulent nematode from infecting this insect^13^.

In a previous work, we have described that the phylogeny of RIP genes shows incongruence with that of the species. Most of these inconsistencies can be explained by gene duplication, loss and/or lineage sorting^11^. Another mechanism leading to phylogenetic incongruence is horizontal gene transfer (HGT); namely the non-genealogical transmission of genes among organisms. HGT is accepted as an important force driving prokaryotic genome evolution^14, 15^. In contrast, its impact on genomes from multicellular eukaryotes, in particular animals, is largely controversial^16–18^. To be maintained permanently in animal species, heritable changes (*i.e*. the transferred gene) must be incorporated into germline cells and transmitted to the offspring. Nevertheless, in the particular case of herbivore arthropods and nematodes, HGT has been postulated to play a role in the adaptation to phytophagy, including the efficient assimilation and detoxification of plant metabolites^19–21^.

Detection of *bona fide* HGT derived genes is not trivial, and careful data revision is required for its corroboration. Many cases of putative foreign genes have been shown, after further revision, to result from artifacts or misinterpretations, such as contamination of genomic data, incomplete sampling of sequences and/or taxa, incorrect phylogenetic inferences or hidden paralogy. Two emblematic cases illustrating these issues are the initial conclusion that the human genome contained a high percent of bacterial derived genes^22^, and the recent claim that tardigrade genomes contain significant amounts of foreign DNA^23^. In both cases, subsequent sounder analyses demonstrated that contamination or incomplete sampling better explained the available data^24, 25^. Consequently, tidy case-by-case analyses of HGT candidates are required for their efficient detection. To do so, independent evidence and alternative evolutionary scenarios should be taken into account.

Based on the previous finding of *in silico* evidence for the presence of genes coding putative proteins harboring RIP domains in the genomes of two closely related species of mosquitoes^11^, we aim to confirm the presence and determine the location of RIP encoding genes in species of the mosquito subfamily Culicinae. Moreover, we provide solid evidence supporting the hypothesis that these genes derive from a single prokaryotic transferred gene.

## Results

### *Culex* spp genomes harbor RIP encoding genes

**Figure 1:**
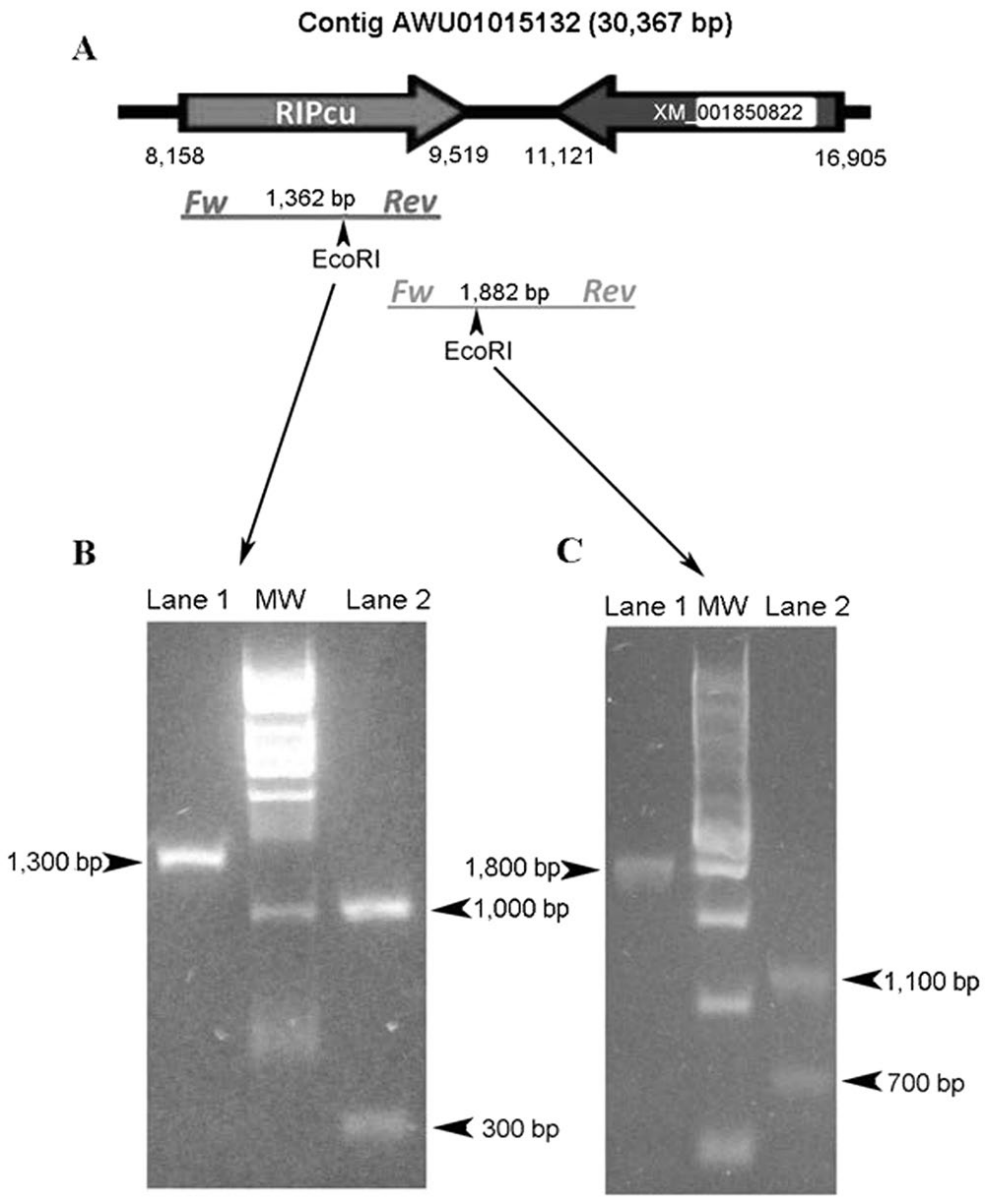
Experimental confirmation of the presence and location of RIP gene in *C. quinquefasciatus* JHB genome. (**A**) Schematic representation of a fragment of the contig AAWU01015132 depicting the RIP gene (RIPcu) and its closest neighbor gene (XM_001850822). Expected amplicons and relevant EcoRI restriction sites are also presented. The intron of XM_001850822 is represented with a white box. (**B**) RIP ORF was amplified by PCR and the product was analyzed by gel electrophoresis before (lane 1) and after EcoRI treatment (lane 2). (**C**) A fragment of 1,882 bp linking the RIP gene with its neighbor-gene was amplified and electrophoresed before (lane 1) and after EcoRI treatment (lane 2).

Recently, we found *in silico* evidence for the presence of genes coding for RIP-containing proteins in two closely related species of Metazoa: *Aedes aegypti* and *Culex quinquefasciatus* ^11^. These intriguing findings led us to design experimental strategies to confirm their presence by ruling out possible database artifacts (*i.e*. contamination). For this purpose, genomic DNA was obtained from a pool of four mosquitoes of *C. quinquefasciatus* strain JHB (same strain as the originally sequenced and available in GenBank). Then, two independent PCR experiments were designed to demonstrate the presence of the intronless RIP gene, and to confirm its physical linkage to the predicted neighbor gene, which is an intron-containing metazoan-derived gene (XM_001850822). Figure 1 shows that both PCR products presented the expected size. Also, further cleavage of each amplicon with EcoRI yielded the predicted patterns, confirming their identity. We have also successfully amplified partial coding regions of two putative RIP genes from *Ae. aegypti* (data not shown).

Once the presence and location of the RIP sequence in *C*. *quinquefasciatus* genome were experimentally confirmed, we successfully amplified the full length RIP coding sequences (~1300 bp) of the closely related species *C. pipiens*, *C. molestus*, and *C. torrentium* (Supplementary Figure 1). PCR products were cloned and sequenced, and the obtained sequences were aligned. As expected, nucleotide sequences showed high similarity (93–97% identity) in relation to the reported sequence in the *C. quinquefasciatus* genome database. The reference sequence of *C. quinquefasciatus* JHB (RipCu) obtained from the genome database revealed an in-frame, three-nucleotide (ACC) insertion (encoding an additional Thr residue). Interestingly, the sequence obtained from the *C. quinquefasciatus* JHB MR4-CDC colony harbors a ten-nucleotide frame-shifting deletion (nt 542–551) generating a premature stop codon disrupting the RIP domain (Supplementary Figure 2). By direct sequencing of PCR products from six individual specimens of *C. quinquefasciatus* JHB from the MR4-CDC colony, we confirmed all these individuals were homozygous for the deletion, strongly suggesting this null mutation was fixed in this colony (Supplementary Figure 3).

### Culicinae RIP genes are monophyletic and syntenic

**Figure 2:**
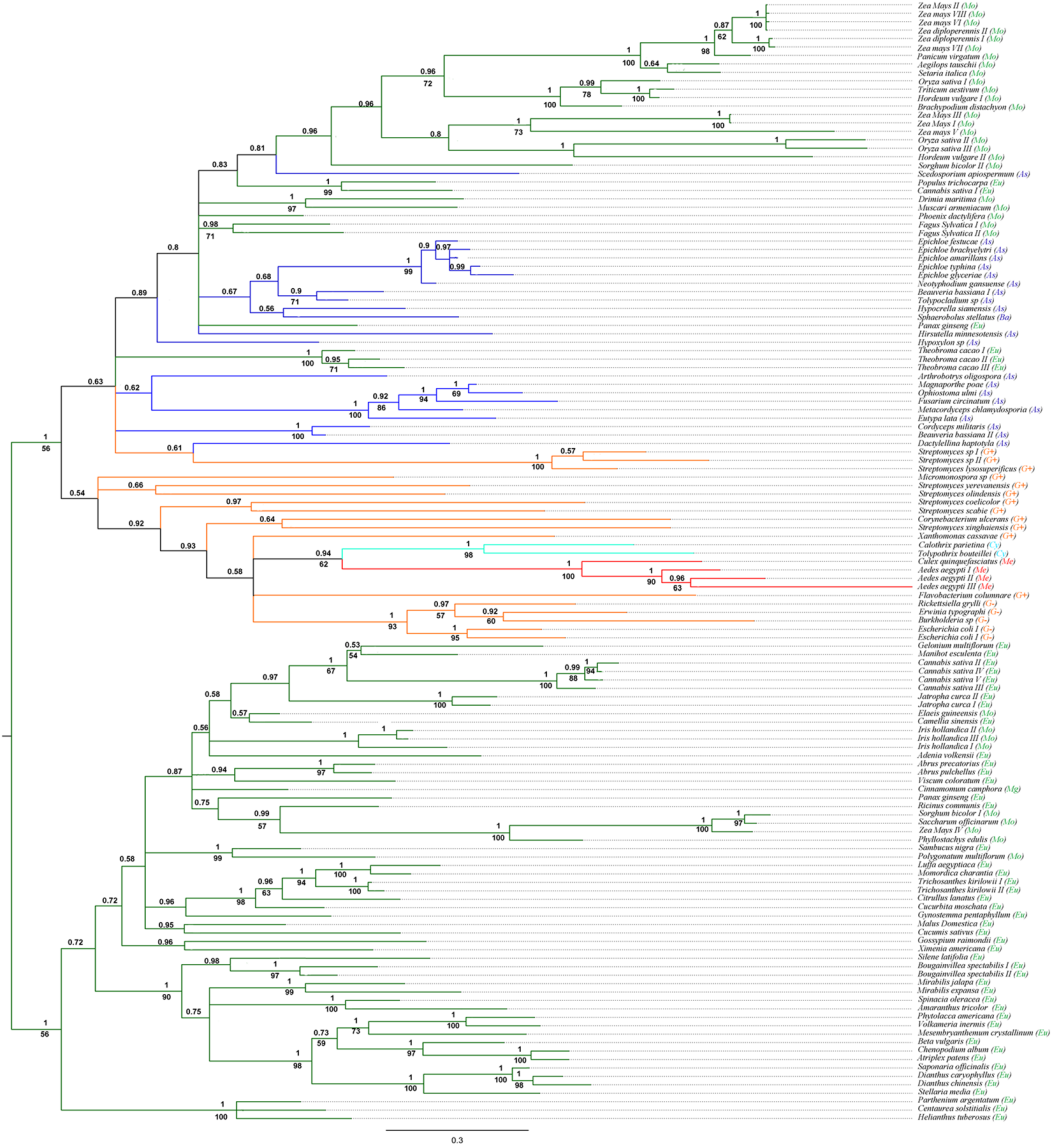
Phylogenetic tree of RIP proteins family. Bayesian tree topology based on a matrix analysis of 133 proteins sequences with 209 informative sites is presented. Numbers above branches indicate PP support values. Bootstrap values (BS) >50% are shown below branches for nodes where topology of ML analysis was coincident with Bayesian inference. Lineages are indicated by different colors as follows: green (Plantae, including Mo: monocots; Eu: eudicots and Mg: magnoliids), blue (Fungi, including As: Ascomycota and Ba: Basidiomycota), red (Me: Metazoa), orange (Bacteria, including G+: Gram positive, G-: Gram negative, and excluding Cyanobacteria) and turquoise (Cy: Cyanobacteria). The clade of Culicinae RIPs is emphasized with red branches. Information related to the sequences used to infer these trees is available in Supplementary Table 2.

**Figure 3:**
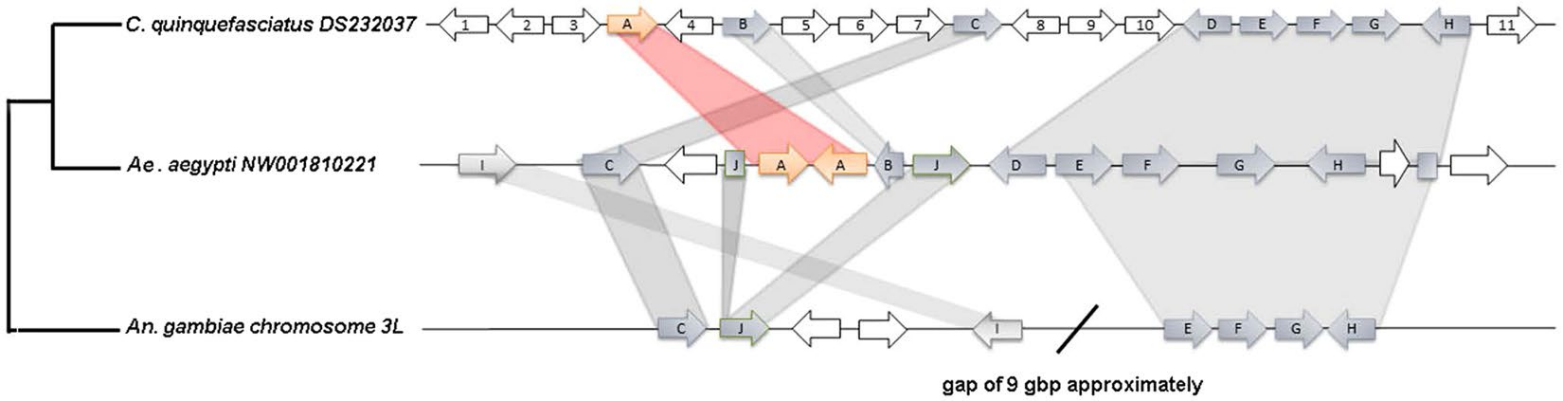
Schematic representation of the shared genomic context between *C. quinquefasciatus* (DS232037), *Aedes aegypti* (NW001810221) and *Anopheles gambiae* (chromosome 3L). Grey shadows link conserved syntenic ORFs. RIP genes are represented with orange arrows. The ORF 4 is equivalent to XM_001850822 and is absent in other scaffolds. Additional information about each gene is available in Supplementary Table 1.

We have previously shown that RIP encoding genes from particular lineages (*e.g*. monocots or dicots, bacteria and fungi) are not monophyletic^11^. Moreover, many RIP clades include sequences belonging to largely distant taxa^11, 26^. Based on this evidence, we postulated that the evolutionary history of RIP genes is consistent with the existence of several ancient paralogues, followed by multiple lineage-specific gene duplications and losses^11^. However, metazoan RIP genes are particularly interesting because they are restricted to closely related insects of the subfamily Culicinae (therefore hereafter referred as Culicinae RIPs). As can be seen in Fig. 2, RIPs from *C. quinquefasciatus* and *Ae. aegypti* form a well-supported clade [posterior probability (PP): 1, bootstrap (BS): 100%]. Monophyly of Culicinae RIPs along with their apparent narrow taxonomic distribution, suggest that these genes are derived from a rather recent, single ancestral sequence. In order to test this, microsynteny analyses were carried out using scaffolds from *Ae. aegypti, C. quinquefasciatus* and *Anopheles gambiae* (the closest relative lacking RIP genes). As expected, partially conserved syntenic blocks were identified in the three species (Fig. 3 and Supplementary Table 1).

### Culicinae RIP genes are derived from a single HGT event

Sequence similarity searches further confirmed that metazoan RIP genes are restricted to the subfamily Culicinae. In addition to the above described genes, we found *in silico* seven RIP genes in *Ae. albopictus* (GenBank: KXJ78156, KXJ78155, KXJ78158, KXJ73132, KXJ78157, KXJ73133 and KXJ73764). A more detailed analysis on the evolutionary history of mosquito RIPs (including synteny analysis and hypothesis on gene duplications and losses) can be found in the Supplementary Data File 1. In addition, two transcriptomic sequences from another Culicinae mosquito; *Armigeres subalbatus*, partially covering the ORFs (GenBank: EU212208, EU211398) were found. In light of these findings, two alternative hypotheses were postulated:

(i) These genes have been vertically inherited from the metazoan cenancestor, and were purged from other metazoan genomes by a number of independent gene loss events.
(ii) These genes are derived from a unique HGT event which took place in the common ancestor of the *Culex* and *Aedes* species.

Regarding the first alternative, the minimal number of independent gene losses required was determined in order to evaluate the plausibility of the vertical transmission hypothesis (see Supplementary Data File 2 for details). Following a conservative approach, at least 15 gene losses should be postulated, from Bilateria to Culicinae, to explain the narrow taxonomic distribution of RIP encoding genes in Metazoa. The HGT hypothesis involves the loss of the ancestral RIP genes before the origin of Metazoa, followed by a single recent HGT event to the ancestor of *Culex* and *Aedes*, yielding a more parsimonious evolutionary scenario.

**Figure 4:**
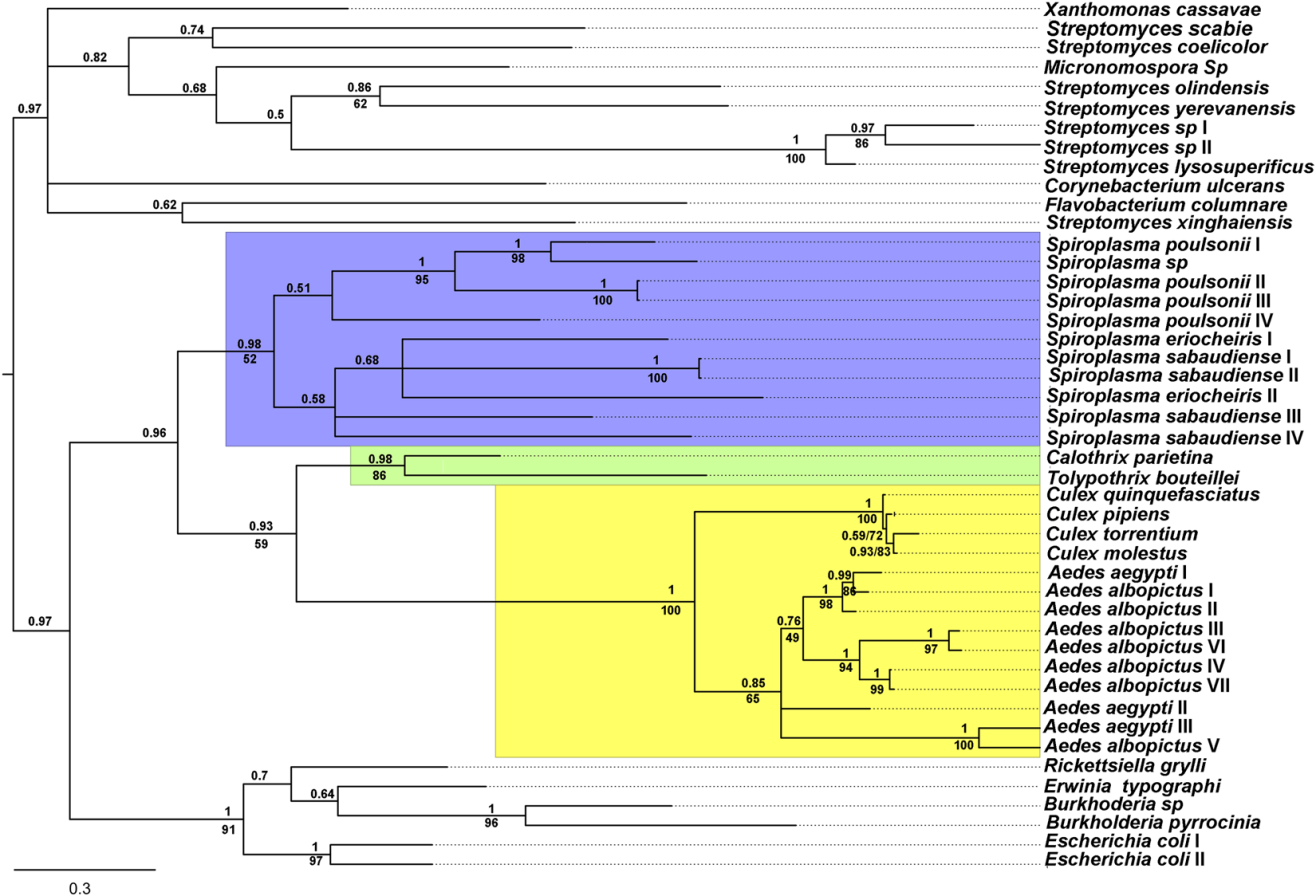
Phylogenetic relationships among Culicinae and bacterial RIPs. Bayesian tree topology based on matrix analysis of 45 proteins sequences with 275 informative sites is presented. Numbers above branches indicate PP support values. Bootstrap values (BS) >50% are shown below branches for nodes where topology of ML analysis was coincident with Bayesian inference. Light blue, green and yellow backgrounds indicate *Spiroplasma*, *Cyanobacteria* and mosquito RIPs, respectively. Information related to the sequences used to infer these trees is available in Supplementary Table 2.

Based on the strong evidence supporting that Culicinae RIPs are derived from an HGT event, a search for possible donors was conducted. The phylogeny shows that Culicinae RIPs form a well-supported monophyletic group (PP: 1, BS: 100%) embedded within bacterial sequences (Fig. 2), suggesting a prokaryotic origin. The lack of introns in Culicinae RIPs also supports this fact. Moreover, homology searches using DELTA-BLAST tool (in Bacteria taxa) and using one of the *Ae. aegypti* RIPs (XP_001650164) as query, yielded sequences belonging to *Calothrix parietina* (Cyanobacteria), and *Xanthomonas cassavae* (Xanthomonadales). In a second iteration using these sequences, additional bacterial sequences are retrieved, including those belonging to *Tolypothrix bouteillei* (Cyanobacteria) and *Spiroplasma poulsonii* (Tenericutes). In order to perform a more robust study we focused on Culicinae, including the recently reported sequences from *Ae. albopictus*, and on bacterial RIPs. Phylogenetic analysis showed that Culicinae RIPs form a monophyletic group (PP: 0.93, BS: 59%) with sequences from Cyanobacteria. This clade also groups with *Spiroplasma* spp sequences (PP: 0.96) although with low bootstrap support (BS: 30%) (Fig. 4). Thus, these organisms −or others closely related- could have been potential donors.

### Horizontally acquired RIP genes evolve under purifying selection pressure

**Figure 5:**
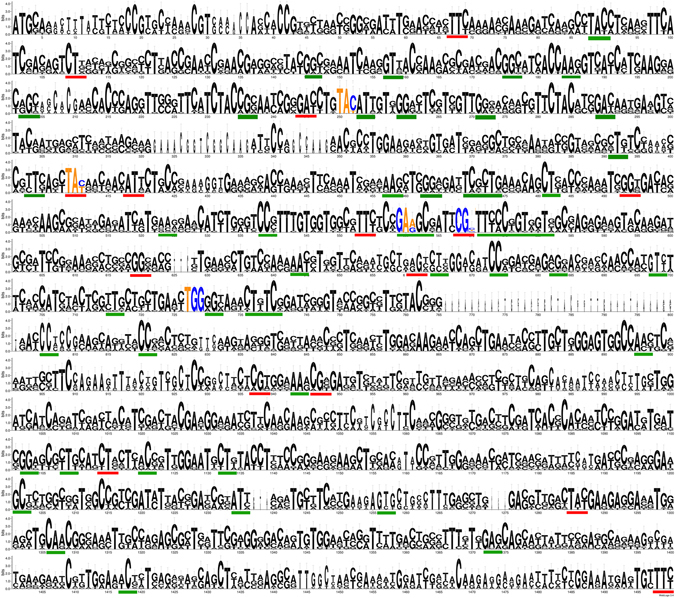
Analyses of the synonymous *vs*. nonsynonymous substitution rates. Codons forming the active site are indicated by colored nucleotides (A and T: orange, G and C: blue). Codons under significant purifying (negative) selection determined by the three tests (SLAC, FEL and REL), or by two out of the three tests, are underlined in red or green color, respectively.

Except for a few well characterized potent toxins (*e.g*. ricin, shiga and shiga-like), the physiological role of most RIPs remains unknown^27^. The defensive role demonstrated for *Spiroplasma* RIP genes in *Drosophila neotestacea* ^13^ induced us to postulate a biological function for the foreign RIP genes in mosquitoes. In addition, searches on transcriptomic databases revealed that RIP genes from *C. quinquefasciatus*, *Ae. aegypti* and *Ae albopictus* are expressed at the RNA level (see Supplementary Data File 1). Besides, as mentioned above, two transcriptomic sequences from *Armigeres subalbatus* have also been deposited (GenBank: EU212208, EU211398). However, mRNA expression is needed for, but not proof of biological significance. Therefore, we searched for traces of selective pressure (i.e. impact on the fitness) on HGT-derived sequences, as reliable evidence of functionality^28^. To do this, all RIP encoding sequences from *C. quinquefasciatus*, *C. molestus*, *C. pipiens*, *C. torrentium*, *Ae. Aegypti*, and *Ae. albopictus* were aligned. Interestingly, the observed INDELs were always multiple of three nucleotides, strongly suggesting that frame-shifting mutations were actively purged by negative selection pressure (except for the RIP sequence of *C. quinquefasciatus* JHB MR4 mentioned above; see discussion below). Figure 5 shows a logo representation of the MSA, where higher conservation of first and second codon position seems to be the rule. Moreover, an integrative analysis of synonymous *vs*. nonsynonymous substitutions employing three different methods; SLAC, FEL and REL (see Materials and Methods) showed purifying (negative) selection for 64 codons, including most of the amino acids forming the active site (Fig. 5 and Supplementary Table 3). Additionally, the global nonsynonymous (Ka)/synonymous (Ks) rate (ω value) was calculated for whole coding sequences of Culicinae RIPs. This yielded a ω = 0.23877, being values of less than 1 indicative of purifying selection. Overall, our results suggested that Culicinae RIP genes have been under purifying (negative) selection, supporting the idea that these genes have biological significance.

## Discussion

Ribosome inactivating proteins form a very interesting protein family displaying a patchy taxonomic distribution. As it was mentioned above, in a previous report we have found *in silico* evidence of the presence of RIP genes in two closely related species of mosquitoes^11^. Due to the uniqueness of this finding, in this work we have experimentally confirmed the presence and location of RIP genes in the *C. quinquefasciatus* genome, as well as in other *Culex* species. Moreover, new exhaustive searches on metazoan databases, revealed the presence of additional homologous genes in *Ae. albopictus* and *Armigeres subalbatus*, confirming the RIP gene family is taxonomically restricted to the Culicinae subfamily (Supplementary Data Files 1 and 2).

Currently, there is not a “gold standard” methodology for automatic and reliable detection of HGT. Moreover, several reports claiming the presence of foreign genes have been undermined after more thorough analyses due to artifacts or misinterpretations^22, 29–31^. Therefore, careful integration of information derived from taxonomic distribution, phylogenetic inferences and biological information is needed to detect *bona fide* horizontally acquired genes. The evidence obtained in this work shows that the most plausible origin of Culicinae RIPs is a single HGT event to the cenancestor of *Culex*, *Aedes* and *Armigeres* genera. This model is supported by the monophyly of metazoan RIPs and their very narrow taxonomic distribution, the gathering into a clade along with prokaryotic sequences and the shared genomic context among Culicinae species.

According to phylogenetic inferences (Figs 2 and 4), the donor of the RIP gene was, most likely a prokaryotic organism. An obvious donor candidate for insects is *Wolbachia* spp, since several HGT events between these bacteria and arthropods have been clearly documented^32–34^. It is expected that animal genomes are marginally affected by HGT because of the separation of the germline from somatic cells. This barrier to HGT -known as Weismann barrier- is not present in the case of bacteria infecting germline cells as *Wolbachia* spp, which is consistent with the relatively high number of *Wolbachia* to insect HGT events^16^. However, no RIP encoding sequences can be found in any of the *Wolbachia* spp databases (including 27 fully sequenced genomes). Interestingly, HMMER searches showed hypothetical proteins harboring the RIP domain on Tenericutes class, specifically in *Spiroplasma* species. The fact that *Spiroplasma* species lack a cell wall and are frequent endosymbionts of arthropods makes them logical donor candidates. However, considering *Spiroplasma* sp. as donor involves two major drawbacks. *Spiroplasma* coding sequences harbor very low GC content (around 23%), whereas Culicinae RIP genes range from 39.8% to 55.6% (Table 1). Secondly, *Spiroplasma* spp. and some species of *Millicutes* use a non-universal UGA tryptophan codon. This variation in the genetic code is presumed to have occurred in the early divergence of these genera (dating 250 mya approximately)^35^ while the Culicinae subfamily has diverged more recently (between 51 to 204 mya)^36, 37^. Therefore, the transferred genes containing the non-universal UGA tryptophan codons would be read as a stop. Although GC content of a transferred functional gene could be gradually modified by amelioration, the reversion of several nonsense codons to Trp does not seem plausible. Therefore, these pieces of evidence lead us to reject that Culicinae RIP genes are derived from *Spiroplasma*.

**Table 1:** GC content analysis.

Organism	RIP name	GenBank	GC %	Average GC^*^
*Culex quinquefasciatus*	RIPcu	XM_001850821.1	53.7	51.07%
*Culex pipiens*	RIPpi	KX674697	55.6	52.48%
*Aedes aegypti*	RIPaeI	XM_001650114.1	50.7	50.65%
	RIPaeI LIKE	AAGE02007824	44.8	
	RIPaeII	XM_001658803.1	39.8	
*Aedes albopictus*	RIPalI	JXUM01048494	50.9	53.2%
	RIPalII	JXUM01048494	50.2	
	RIPalIII	JXUM01048511	46.5	
	RIPalIV	JXUM01048511	46.9	
	RIPalV	JXUM01085134	45.5	
	RIPalVI	JXUM01090901	46.8	
	RIPalVII	JXUM01090901	47.2	
*Spiroplasma poulsonii*		JHEG02000036.1	26.3	24.90%
		JTLV01000005.1	25.3	
*Tolypothrix sp*		JHEG02000036.1	38.1	41.73%
*Calothrix sp*		CP003610.1	35.8	45.97%
*Ancestral RIP Aedes*			51.1	
*Ancestral RIP Culex*			54.5	
*Ancestral RIP metazoan*			55.4	

The GC content of RIP genes was calculated using DNA/RNA GC Content Calculator (http://www.endmemo.com/bio/gc.php). ^*^The average of GC content in coding regions was obtained from the codon usage database (http://www.kazusa.or.jp/codon/).

The phylogenetically closest sequences to Culicinae RIPs belong to two cyanobacteria (*Tolypothrix bouteillei* and *Calothrix parietina*). Cyanobacteria constitute a significant fraction of the microbiota at breeding sites of mosquitoes. Remarkably, cyanobacterial species account for 40% of the bacterial midgut content of larval and pupal stages in *An. gambiae* ^38^. Moreover, *Calothrix sp*. has been detected in the midgut bacterial flora of *An. stephensi* in larval stage^39^. In addition, the GC content of cyanobacterial genomes is closer to the Culicinae RIPs (Table 1). Altogether, the presented phylogenetic inferences, the shared ecological niches between these bacteria and insects, and the colonization of mosquitoes in their early developmental stages^38^ strongly suggest that Cyanobacteria is the most plausible donor lineage of RIP genes to the subfamily Culicinae via HGT. In line with Huang^16^ we postulate that Weismann barrier is, if not absent, markedly weakened in the egg, pupal, and larval stages of mosquitoes. Consequently, these early developmental stages could be particularly prone to the acquisition of heritable foreign genes by environmental bacteria.

An important question about the HGT derived genes is their fate. In other words, the issue at stake is whether “foreign” genes will have an impact on fitness, and if so, to what extent these genes will be affected by natural selection and/or genetic drift. Probably, mosquito RIP genes display a defensive role, which has helped them to be fixed by natural selection. However, the fact that individuals from the *C. quinquefasciatus* MR4 colony are homozygous for a null mutation in the RIP gene shows it is not essential for viability under laboratory conditions. On the other hand, evolutionary analyses of mosquito sequences revealed evidence of purifying (negative) selection both at the whole-sequence level (ω < 1) and at several codons, which is also reflected by the occurrence of the majority of changes at the third codon position (Fig. 5). According to the nearly-neutral evolutionary theory, slightly deleterious mutations can be fixed in populations with low effective size, as in the case of laboratory colonies. On the contrary, these mutations are efficiently purged from larger populations, such as natural populations^40^. This seems to be the case of Culicinae RIPs, since null mutant *C. quinquefasciatus* are viable in captivity, while clear evidence of selection pressure on coding sequences in wild specimens has been found.

## Materials and Methods

### PCR experiments

PCR experiments were conducted to confirm the presence of RIP encoding sequences in selected organisms. Individuals of *C. quinquefasciatus* strain JHB were obtained from the MR4 colony (Malaria Research and Reference Reagent Resource Center) of the Center for Diseases Control and Prevention (CDC). Genomic DNA from these specimens was extracted employing the protocol previously described by Collins^41^. Genomic DNA from wild specimens of *C. molestus, C. pipiens* and *C. torrentium* was kindly provided by Dr. Stefanie Becker (Bernhard Nocht Institute for Tropical Medicine, Hamburg)^42^. Primer sets were designed to amplify the full-length RIP coding sequence of *C. quinquefasciatus* and also to identify the predicted neighbor gene (Supplementary Table 4). High fidelity *Phusion* DNA polymerase (New England Biolabs) was used under the following PCR conditions: initial denaturation for 30 s at 98 °C, followed by 35 cycles of denaturation (10 s at 98 °C), annealing (30 s at a particular Ta for each primer pair, see Supplementary Table 4) and extension (30 s at 72 °C), and a final extension at 72 °C for 10 min. PCR products were cloned into the pGEMT-easy^®^ vector (Promega) following standard methods, and sequenced. Alternatively, PCR products from individual specimens belonging to the MR4-CDC colony were directly sequenced. The obtained sequences are available under the following GenBank accession numbers: KX674699, KX674697, KX644696, and KX674698.

### Data collection and Multiple Sequence Alignments (MSAs)

We used a previously reported alignment (Data Set S1 in ref. 11) as a matrix to perform searches on reference proteomes using the hmmsearch tool (http://www.ebi.ac.uk/Tools/hmmer/search/hmmsearch)^43^ under default parameters. Although this search strategy is powerful, it is designed to explore only protein databases. Therefore, each one of the retrieved sequences was used as query to perform tBLASTn searches under default parameters against different nucleotide (WGS, ESTs, nr/nt, refseq-rna) databases. In order to confirm the absence of RIP genes in relevant lineages (e.g. Archaea, metazoan other than Culicinae) searches were performed with taxonomic restriction. All retrieved sequences were curated by confirming the presence of a canonical RIP domain (PF00161) using the Pfam server (http://pfam.xfam.org/) and those amino acids predicted to form the active site. The conserved region from new sequences was selected and used for constructing MSAs using the MAFFT 7 online server (http://mafft.cbrc.jp/alignment/server). A few regions where the alignment showed rare insertions exclusively found in a group of sequences with high sequence identity among them were manually removed by blocks. Even though the alignment was difficult as a consequence of the high divergence of the sequences, we confirmed that residues predicted to form the active sites were correctly aligned.

### Phylogenetic inferences

The MSAs obtained were used to perform phylogenetic analysis using Bayesian and Maximum Likelihood inference methods. The substitution matrix and gamma distribution model with invariable sites were calculated using ProtTest3.4^44^ with WAG selected always as the best model. PhyML^45^ was run using the algorithm Tree-Bisection-Reconnection (TBR) with 5 random starting trees. To estimate the robustness of the phylogenetic inference, we ran 100 bootstrap (BS) replicates. Bayesian inferences were performed using Mr. Bayes 3.2 software^46^. A mixed amino acid substitution model was set up, and WAG was retrieved as the best fit model, 4 gamma categories and a proportion of invariable sites were considered. The analyses were concluded after 2,000,000 generations when the split frequency was <0.02. FigTree 1.4.2 software was used to visualize and edit the trees. Sequence alignments (in Fasta format) and phylogenetic trees (in Newick format) used for constructing Figs 2 and 4 are in Supplementary Data File 3.

### Genomic context analyses

The complete sequence of the scaffolds containing RIPs of *C. quinquefasciatus* (DS232037), *Ae. aegypti* (NW001810221) and *An. gambiae* (chromosome 3 L) were subjected to BLASTx searches on different protein databases to identify individual genes upstream and downstream of the RIP open reading frame (ORF). The retrieved sequences were subjected to reciprocal BLASTp and tBLASTn searches in order to determine putative orthologous sequences. Orthologs were confirmed using the comparative genomic tool available at VectorBase website (https://www.vectorbase.org/).

### Evolutionary analyses

Nucleotide encoding RIP sequences found in mosquitoes were aligned by codons, using PAL2NAL algorithm^47^. Then, the codons under purifying (negative) selection were estimated employing the Single Likelihood Ancestor Counting (SLAC), Fixed Effects Likelihood (FEL), and Random Effects Likelihood (REL) tests, available at the Datamonkey Package^48, 49^. The omega (ω) value represented the global ratio between nonsynonymous and synonymous mutations and was assessed using the ML method of CodeML in PAML^50, 51^. This analysis was performed under the one ratio model (M0). Finally, a sequence logo was constructed employing the codon alignment using the Weblogo server^52^ and codons under purifying selection were highlighted.

## Electronic supplementary material

Supplementary information 1

Supplementary information 2
