# Supplementary material for: Metazoan Ribosome Inactivating Protein encoding genes acquired by Horizontal Gene Transfer
Source: Sci Rep. 2017 May 12;7:1863. doi: 10.1038/s41598-017-01859-1 (PMC5431988; doi:10.1038/s41598-017-01859-1)
Supplement: Supplementary file 1 — Supplementary information 1 [file 41598_2017_1859_MOESM1_ESM.pdf]

Metazoan Ribosome Inactivating Protein encoding genes acquired by Horizontal Gene Transfer

Walter J. Lapadula[1*], Paula L. Marcet[2], María L. Mascotti[1], M. Virginia Sanchez-Puerta[3], Maximiliano Juri Ayub[1*]

1. Instituto Multidisciplinario de Investigaciones Biológicas de San Luis, IMIBIO-SL-CONICET and Facultad de Química, Bioquímica y Farmacia, Universidad Nacional de San Luis, San Luis Argentina.

2. Centers for Disease Control and Prevention, Division of Parasitic Diseases and Malaria, Atlanta, USA.

3. IBAM, Universidad Nacional de Cuyo, CONICET, Facultad de Ciencias Agrarias, Almirante Brown 500, M5528AHB, Chacras de Coria, Argentina.

*Corresponding authors: mjuriayub@hotmail.com, wlapadula@gmail.com

Supplementary Information

Supplementary Figure 1. PCR amplification of RIP genes in species belonging to the genus *Culex*. *C. pipiens* (C.p, lane 1), C. *molestus* (C.m, lanes 2 and 3) and *C. torrentium* (C.t, lanes 4 and 5). Lane 6: 1Kb molecular weight size marker, lane 7: negative control.

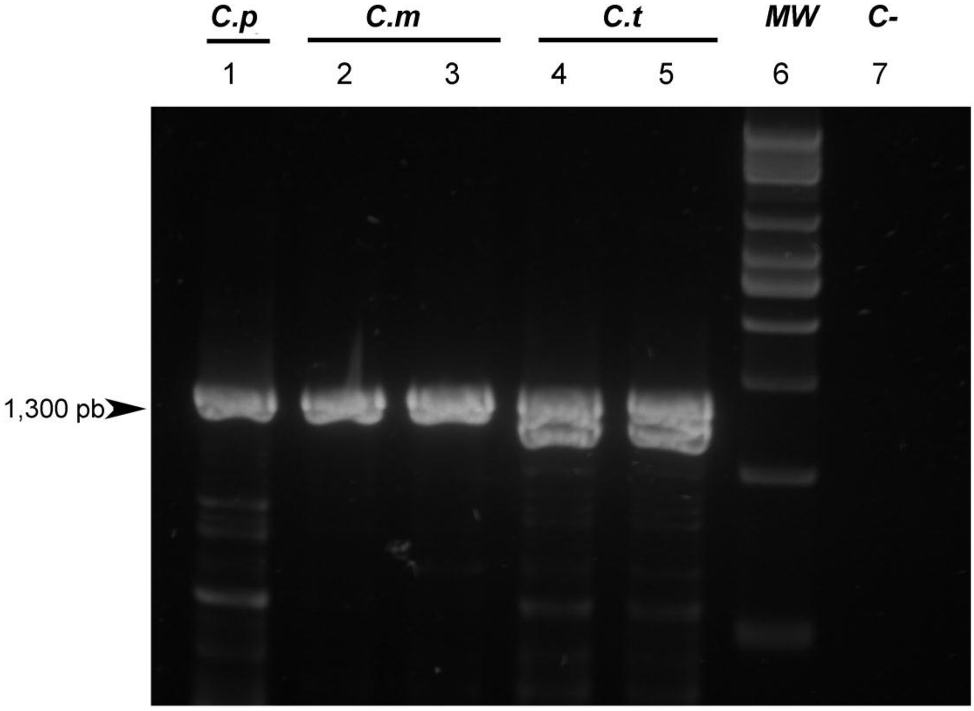

Supplementary Figure 2. Sequence alignment of partial RIP genes in *Culex* spp. Gaps are indicated in green color boxes.

```
RIPcu                 ATGCAGTTCA ACCGCGCCAA ACGTGCCACC ACCACCGTGC GAACGGGCAA CATCGACGAC TTCAAGAAGA
C.quinquefasciatus    ATGCAGTTCA ACCGCGCCAA ACGTGCCACC ACC---GTGC GAACGGGCAA CATCGACGAC TTCAAGAAGA
Culex molestus        ATGCAGTTCA ACCGCGCCAA ACGTGCCACC ACC---GTGC GAACGGGCAA CATCGACGAC TTCAAGAAGA
Culex pipiens         ATGCAGTTCA ACCGCGCCAA ACGTGCCACA TCC---GTGC GAACGGGCAA CATCGACGAC TTCAAGAAGA
Culex torrentium      ATGCAGTTCA ACCGCGCCAA ACGTGCCACC ACC---GTGC GAACGGGCAA CATCGACGAC TTCAAGAAGA

                               80         90        100        110        120        130        140
RIPcu                 ACGGCCAAAA CTACCTGACG TTCATCGACA GTCTGCGCGG TGACCTGAGC CAACCGAACG TGGCTCACGG
C.quinquefasciatus    ACGGCCAAAA CTACCTGACG TTCATCGACG GTCTGCGCGG TGACCTGAGC CAACCGAACG TGGCTCACGG
Culex molestus        ACGGCCAAAA CTACCTGACG TTCATCAACA GTCTGCGCGG TGACCTGAGC CAACCGAAAG TGGCTCACGG
Culex pipiens         ACGGCCAAAA CTACCTGACG TTCATCGACG GTCTGCGCGG TGACCTGAGC CAACCGAAAG TGGCCCACGG
Culex torrentium      ACGGCCAAAA CTATCTGACG TTCATCGACG GTCTGCGCGG TGACCTGAGC CAACCGAAAG TGGCTCACGG

                              150        160        170        180        190        200        210
RIPcu                 CGGAATCCGC GTAACGAAAA CCGACAACGG CATCACCAAG GTCATCCTGA AGAGTGGCAC CACCCAGATT
C.quinquefasciatus    CGGAATCCGC GTAACGAAAA CCGACAACGG CATCACCAAG GTCATCCTGA AGAGTGGCAC CACCCAGATT
Culex molestus        CGGAATCCGC GTGACGAAAA CCGACAACGG CATCACCAAG GTCATCCTGA AGAGCGGCTC CACCCAGATT
Culex pipiens         TGGAATTCGA GTAACGAAAA CCGACAACGG CATCACCAAG GTCATCCTGA AGAGCGGCAC CACCCAGATT
Culex torrentium      CGGAATCCGC GTGACGAAAA CCGACAACGG CATCACCAAG GTCATCCTGA AGAGCGGCTC CACCCAGATT

                              220        230        240        250        260        270        280
RIPcu                 CCGTTCATCT ACCGGAACAG CGACCTGTAC ATTGTTGGGT TCGTGGTTGG GTCGGTGTTC TACGCGGACA
C.quinquefasciatus    CCGTTCATCT ACCGGAACAG CGACCTGTAC ATTGTTGGGT TCGTGGTTGG GTCGGTGTTC TACGCGGACA
Culex molestus        CCGTTCATCT ACCGGAACAG CGACCTGTAC ATTGTTGGGT TCGTGGTTGG GTCGGTGTTC TACGCGGACA
Culex pipiens         CCGTTCATCT ACCGGAACAG CGACCTGTAC ATTGTTGGGT TCGTGATTGG GTCGGTGTTC TACGCGGATA
Culex torrentium      CCGTTCATCT ACCGGAACAG CGACCTGTAC ATTGTTGGGT TCGTGGTTGG GTCGGTGTTC TACGCGGACA

                              290        300        310        320        330        340        350
RIPcu                 ATGACGTCTT CAAGTGGATT TCTCCGCGGG AAACGGTCGG CAAGTTGCCG GCCAAGCACG CCTGGAGGTC
C.quinquefasciatus    ATGACGTCTT CAAGTGGATT TCTCCGCGGG AAACGGTCGG CAAGTTGCCG GCCAAGCACG CCTGGAGGTC
Culex molestus        ACGATGTCTT CAAGTGGATT TCTCCGCGGG AAACGGTCGG CAAGTTGCCG GCCAAGCACG CCTGGCGGTC
Culex pipiens         ATGACGTCTT CAAGTGGATT TCTCCGCGGG AAACGGTCGG CAAGTTGCCG GCTAAGCACG CCTGGAGGTC
Culex torrentium      ACGATGTCTT CAAGTGGATT TCTCCGCGGG AAACGGTCGG CAAGTTGCCG GCCAAGCACG CCTGGAGGTC

                              360        370        380        390        400        410        420
RIPcu                 GGTGCTGGGG GATGTCACGG TGGATACGCT GTCGGTTCCG CTGAGTTACA ATAACATCCT GCAGGGGCGC
C.quinquefasciatus    GGTGCTGGGG GATGTCAGCG TGGACACGCT GTCGGTGCCG CTGAGTTACA ACAACATCCT GCAGGGGCGC
Culex molestus        GGTGCTGGGG GATGTCAGCG TGGACACGCT TTCGGTAGCG CTGAGTTACA ACAACATTCT GCAGGGGCGC
Culex pipiens         GGTGCTGGGG GATGTCACGG TGGACACGCT TTCTGTGCCG CTGAGTTACA ACAACATCCT GCAGGGACGC
Culex torrentium      GGTGCTGGGG GATGTCAGCG TGGACACGCT GTCGGTAGCG CTGAGTTACA ACAACATTCT GCAGGGGCGC

                              430        440        450        460        470        480        490
RIPcu                 AGTAAGGTTC CGATCAACAA GATGGGAGAT TCGCTGAAGA AGCTGACCGA GGTGGGAGAT CGGAACAAGA
C.quinquefasciatus    AGTAAGGTTC CGATCAACAA GATGGGAGAT TCGCTGAAGA AGCTGACCGA GGTGGGAGAT CGGAACAAGA
Culex molestus        AGTAAGGTTC CGATCAACAA GATGGGAGAT TCGCTGAAGA AGCTGACCGA GGTGGGAGAT CGAAACAAGA
Culex pipiens         AGTAAGGTTC CGATCAACAA GATGGGAGAT TCGCTGAAGA AGCTGACCGA GGTGGGGGAT CGGAACAAGA
Culex torrentium      AGTAAGGTTC CGATCAACAA GATGGGAGAT TCGCTGAAGA AGCTGACCGA GGTGGGGGAT CGAAACAAGA

                              500        510        520        530        540        550        560
RIPcu                 AGAGTGCGAT CGTCAAGGAA CATCTGGTAC CGTTTGTGGT GGCGTTTTCC GAGGCGATCC GCTTCACGGT
C.quinquefasciatus    AGAGTGCGAT CGTCAAGGAA CATCTGGTAC CGTTTGTGGT GGCGTTTTCC G--------- -CTTCACGGT
Culex molestus        AGAGTGGGAT CGTCAAGGAA CATCTGGTTC CGTTTGTGGT GGCGTTCTCC GAGGCGATCC GCTTCACGGT
Culex pipiens         AGAGTGCGAT CGTCAAGGAA CATCTGGTTC CGTTTGTGGT GGCGTTTTCC GAGGCGATCC GCTTCACGGT
Culex torrentium      AGAGTGGGAT CGTCAAAGAA CATCTGGTTC CGTTTGTGGT GGCGTTCTCC GAGGCGATCC GCTTCACGGT

                              570        580        590        600        610        620        630
RIPcu                 GGTCGCTAGG GCCGTGAGGG ATGCCTTCGT GAAGAATGGA GGGGAGTTGG ACATGAAGAC GAACGTGCTG
C.quinquefasciatus    GGTCGCTAGG GCCGTGAGGG ATGCCTTCGT GAAGAATGGA GGGGAGTTGG ACATGAAGAC GAACGTGCTG
Culex molestus        GGTCGCTAGG GCCGTGAGGG ATGCCTTCGT GAAGAATGGA GGGGAGTTGG ACATGAAGAC GAACGTGCTG
Culex pipiens         GGTGGCCAGG GCTGTGAAGG ATGCCTTTGT GAAGAATGGA GGGGAGTTGG ACATGAAAAC GAACGTGTTG
Culex torrentium      GGTCGCTAGG GCTGTGAGGG ATGCCTTCGT GAAGAATGGA GGGGAGTTGG ACATGAAGAC GAACGTGCTG
```

Supplementary Figure 3. Electropherograms of the region encompassing the deletion of ten nucleotides in *C. quinquefasciatus* JHB MR4 colony. Top: sequence of *C. quinquefasciatus* JHB MR4 colony displaying the ten nucleotides deletion. Bottom: Chromatogram of *C. molestus* RIP sequence where no deletion is observed.

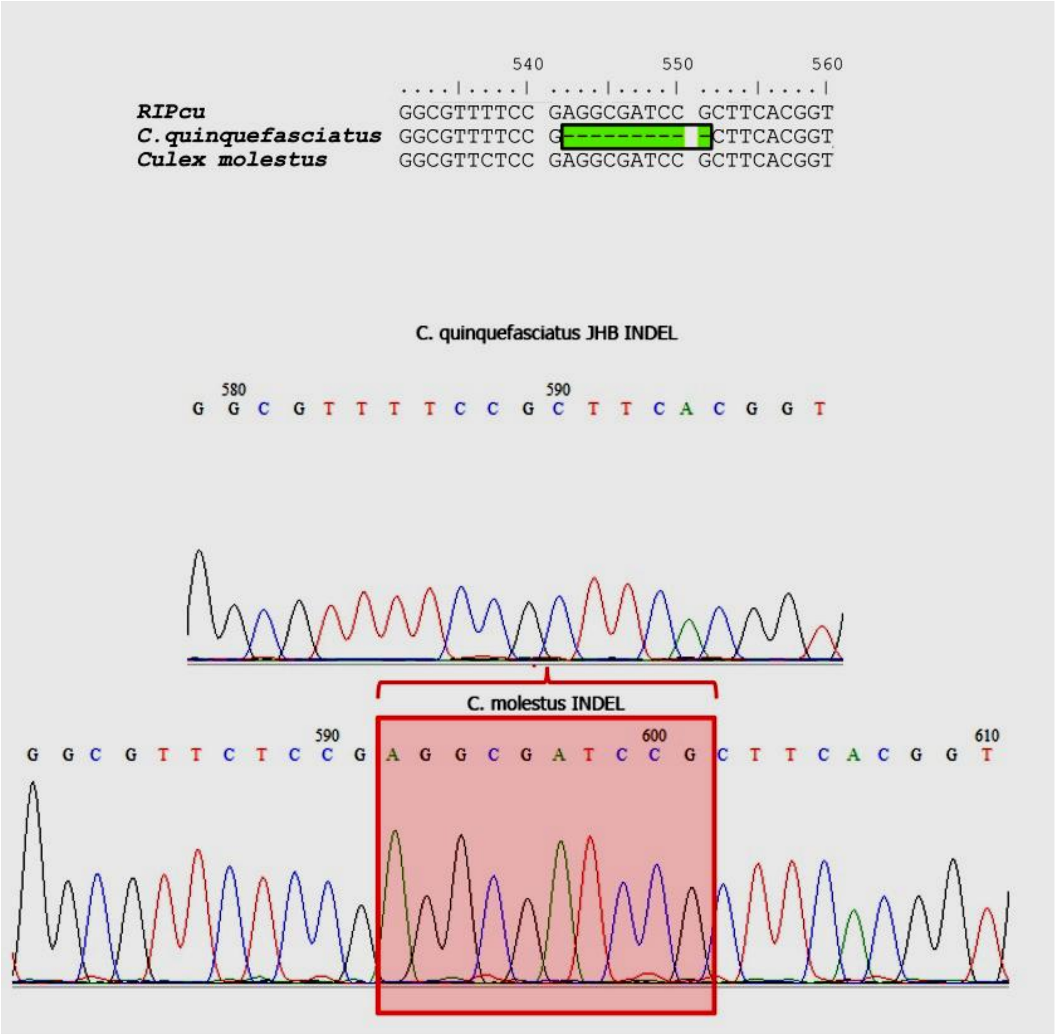

Supplementary Table 1. Orthologous genes among *C. quinquefasciatus*, *Ae. aegypti* and *An. gambiae.* The GenBank and Vector Base codes are indicated for all protein sequences. The code of each protein used in Figure 3 is shown in the last column.

C. quinquefasciatus		*Ae.. aegypti*			*An. gambiae*			
GenBank	VectorBase Code	GenBank	VectorBase Code	E-value	GenBank	VectorBase Code	E-value	Figure Code
XP_001850870	CPIJ009208							1
XP_001850871	CPIJ009209							2
XP_001850872	CPIJ009210							3
XP_001850873	CPIJ009211	XP_001650164	AAEL005004	1 e-93				**A**
XP_001850874	CPIJ009212							4
XP_001850875	CPIJ009213	XP_001650165	AAEL004999	5 e-65				**B**
XP_001850876	CPIJ009214							5
XP_001850877	CPIJ009215							6
XP_001850878	CPIJ009216							7
XP_001850879	CPIJ009217	XP_001650161	AAEL005011	0	XP_309724	AGAP010970	0	**C**
XP_001850880	CPIJ009218							8
XP_001850881	CPIJ009219							9
XP_001850882	CPIJ009220							10
XP_001850884	CPIJ009222	XP_001650166	AAEL005012	0	XP_310862	AGAP000256	0	**D**
XP_001850885	CPIJ009223	XP_001650167	AAEL005003	0	XP_318236	AGAP010327	0	**E**
XP_001850886	CPIJ009224	XP_001650168	AAEL005001	6 e-138	XP_554502	AGAP010326	6 e-87	**F**
XP_001850887	CPIJ009225	XP_001650169/70/71	AAEL005008	1 e-153	XP_318238	AGAP010325	8 e-138	**G**
XP_001850888	CPIJ009226	XP_001650173	AAEL005009	0	XP_318239	AGAP010324	0	**H**
XP_001850889	CPIJ009227							11

Supplementary Table 2. Sequences used in this work. The first column indicates lineages containing RIP encoding genes (bacteria, fungi, metazoan and plant). The second column indicates the specific organisms harboring each gene. The third column indicates the RIP name used for sequence identification in Figures 2 and 4. The fourth column indicates the Genbank accession number or Protein ID, and the fifth column indicates the source of information.

Lineage	Species	Name	GenBank or Protein ID	Source
Bacteria	*Burkholderia sp*	*Burkholderia sp*	ADG20812	Protein
	Burkholderia pyrrocinia	*Burkholderia pyrrocinia*	ALWI01000071	WGS
	Corynebacterium ulcerans	*Corynebacterium ulcerans*	YP005709811	Protein
	Flavobacterium columnare	*Flavobacterium columnare*	YP004942850	Protein
	Streptomyces coelicolor	*Streptomyces coelicolor*	AL939130	WGS
	Streptomyces lysosuperificus	*Streptomyces lysosuperificus*	AGDC01002112	WGS
	Streptomyces sp	*Streptomyces sp I*	EDX20776	Protein
		Streptomyces sp II	EDX25168	Protein
	Streptomyces olindensis	*Streptomyces olindensis*	WP037761173	Protein
	Streptomyces scabie	*Streptomyces scabie*	FN554889	WGS
	Streptomyces yerevanensis	*Streptomyces yerevanensis*	WP033322798	Protein
	Streptomyces xinghaiensis	*Streptomyces xinghaiensis*	AFRP01002228	WGS
	Spiroplasma poulsonii	*Spiroplasma poulsonii I*	WP040093770	Protein
		Spiroplasma poulsonii II	WP040093936	Protein
		Spiroplasma poulsonii III	WP040093936	Protein
		Spiroplasma poulsonii IV	WP040092751	Protein
	Spiroplasma eriocheiris	*Spiroplasma eriocheiris I*	WP047791682	Protein
		Spiroplasma eriocheiris II	AKM54001	Protein
	Spiroplasma sabaudiense	*Spiroplasma sabaudiense I*	WP025251436	Protein
		Spiroplasma sabaudiense II	AHI54300	Protein
		Spiroplasma sabaudiense III	WP025251437	Protein
		Spiroplasma sabaudiense IV	WP025250933	Protein
	Spiroplasma sp	*Spiroplasma sp*	WP025317327	Protein
	Escherichia coli	*Escherichia coli I*	EF441598	DNA
		Escherichia coli II	AF500192	DNA
	Erwinia typographi	*Erwinia typographi*	WP034895486	Protein
	Xanthomonas cassavae	*Xanthomonas cassavae*	WP029217990	Protein
	Micromonospora sp	*Micromonospora sp*	EEP74218	Protein
	Rickettsiella grylli	*Rickettsiella grylli*	WP006035658	Protein
	Calothrix parietina	*Calothrix parietina*	WP015197802	Protein
	Tolypothrix bouteillei	*Tolypothrix bouteillei*	KIE12545	Protein
Fungi	*Arthrobotrys oligospora*	*Arthrobotrys oligospora*	ADOT01000134	WGS
	Dactylellina haptotyla	*Dactylellina haptotyla*	AQGS01000454	WGS
	Cordyceps militaris	*Cordyceps militaris*	AEVU01000543	WGS
	Epichloe glyceriae	*Epichloe glyceriae*	AFRF01000004	WGS
	Epichloe amarillans	*Epichloe amarillans*	AFRB01000334	WGS

	Epichloe typhina	*Epichloe typhina*	ADFL02000265	WGS
	Epichloe festucae	*Epichloe festucae*	AFRG01000322	WGS
	Epichloe brachyelytri	*Epichloe brachyelytri*	AFSE01000013	WGS
	Fusarium circinatum	*Fusarium circinatum*	JRVE01000053	WGS
	Neotyphodium gansuense	*Neotyphodium gansuense*	AFRE01000036	WGS
	Beauveria bassiana	*Beauveria bassiana I*	EJP64846	WGS
		Beauveria bassiana II	EJP61635	WGS
	Tolypocladium sp	*Tolypocladium sp*	JPHH02000315	WGS
	Hypocrella siamensis	*Hypocrella siamensis*	JMQE01001167	WGS
	Metacordyceps chlamydosporia	*Metacordyceps chlamydosporia*	AOSW01007610	WGS
	Hirsutella minnesotensis	*Hirsutella minnesotensis*	JPUM01000046	WGS
	Hypoxylon sp	*Hypoxylon sp*	JYCQ01000009	WGS
	Eutypa lata	*Eutypa lata*	AORF01001931	WGS
	Scedosporium apiospermum	*Scedosporium apiospermum*	JOWA01000098	WGS
	Ophiostoma ulmi	*Ophiostoma ulmi*	AMZD01000108	WGS
	Magnaporthe poae	*Magnaporthe poae*	ADBL01002728	WGS
	Sphaerobolus stellatus	*Sphaerobolus stellatus*	KIJ38555	Protein
Metazoan	*Aedes aegypti*	*Aedes aegypti I*	AAGE02007824	WGS
		Aedes aegypti II	AAGE02007824	WGS
		Aedes aegypti III	AAGE02013700	WGS
	Aedes albopictus	*Aedes albopictus I*	KXJ78155	Protein
		Aedes albopictus II	KXJ78156	Protein
		Aedes albopictus III	KXJ78157	Protein
		Aedes albopictus IV	KXJ78158	Protein
		Aedes albopictus V	KXJ73764	Protein
		Aedes albopictus VI	KXJ73133	Protein
		Aedes albopictus VII	KXJ73132	Protein
	Culex quinquefasciatus	*Culex quinquefasciatus*	AWU01015132	WGS
	Culex molestus	*Culex molestus*	KX674696	Protein
	Culex Pipiens	*Culex Pipiens*	KX674697	Protein
	Culex torrentium	*Culex torrentium*	KX674698	Protein
Plant	*Aegilops tauschii*	*Aegilops tauschii*	EMT05877	Protein
	Brachypodium distachyon	*Brachypodium distachyon*	ADDN01000393	WGS
	Hordeum vulgare	*Hordeum vulgare I*	M62905	mRNA
		Hordeum vulgare II	Q00531	Protein
	Oryza sativa	*Oryza sativa I*	AB051107	mRNA
		Oryza sativa II	AAX95110	Protein
		Oryza sativa III	EEE51701	Protein
	Panicum virgatum	*Panicum virgatum*	FL902192	EST
	Phyllostachys edulis	*Phyllostachys edulis*	FP092597	mRNA
	Saccharum officinarum	*Saccharum officinarum*	CA078533	EST
	Setaria italica	*Setaria italica*	XP004975022	Protein

	Sorghum bicolor	*Sorghum bicolor I*	XM002459546	mRNA
		Sorghum bicolor II	ABXC01000035	WGS
	Triticum aestivum	*Triticum aestivum*	D13795	DNA
	Zea diploperennis	*Zea diploperennis I*	DQ147051	DNA
		Zea diploperennis II	DQ147068	DNA
	Zea mays	*Zea Mays I*	XP008665567	Protein
		Zea Mays II	M77122	mRNA
		Zea Mays III	DAA51819	Protein
		Zea Mays IV	AY105813	mRNA
		Zea mays V	NP001159316	Protein
		Zea mays VI	BT037982	ARNm
		Zea mays VII	L26305	ARNm
		Zea mays VIII	EU725161	ARNm
	Cinnamomum camphora	*Cinnamomum camphora*	AY039801	mRNA
	Elaeis guineensis	*Elaeis guineensis*	EL690296	EST
	Phoenix dactylifera	*Phoenix dactylifera*	ACYX02098007	WGS
	Drimia maritima	*Drimia maritima*	P84786	Protein
	Iris hollandica	*Iris hollandica I*	U78041	mRNA
		Iris hollandica II	AF256085	mRNA
		Iris hollandica III	AF256084	mRNA
	Muscari armeniacum	*Muscari armeniacum*	AF289116	mRNA
	Polygonatum multiflorum	*Polygonatum multiflorum*	AF213983	mRNA
	Cucumis sativus	*Cucumis sativus*	ACHR01003818	WGS
	Citrullus lanatus	*Citrullus lanatus*	AGCB01010835	WGS
	Cucurbita moschata	*Cucurbita moschata*	EU309692	DNA
	Gynostemma pentaphyllum	*Gynostemma pentaphyllum*	DQ672577	DNA
	Luffa aegyptiaca	*Luffa aegyptiaca*	X62372	mRNA
	Momordica charantia	*Momordica charantia*	X57682	mRNA
	Trichosanthes kirilowii	*Trichosanthes kirilowii I*	AY669811	DNA
		Trichosanthes kirilowii II	TKU25675	mRNA
	Abrus pulchellus	*Abrus pulchellus*	EU008736	mRNA
	Abrus precatorius	*Abrus precatorius*	M98344	mRNA
	Adenia volkensii	*Adenia volkensii*	AJ537497	DNA
	Gelonium multiflorum	*Gelonium multiflorum*	L12243	mRNA
	Jatropha curca	*Jatropha curca I*	BABX01103831	WGS
		Jatropha curca II	AY069946	mRNA
	Populus trichocarpa	*Populus trichocarpa*	AARH01008880	WGS
	Ricinus communis	*Ricinus communis*	X52908	DNA
	Manihot esculenta	*Manihot esculenta*	JPQF01109211	WGS
	Sambucus nigra	*Sambucus nigra*	AF249280	mRNA
	Panax ginseng	*Panax ginseng*	DQ683359	mRNA
	Fagus Sylvatica	*Fagus Sylvatica I*	FR617512	mRNA
		Fagus Sylvatica II	FR606563	mRNA
	Amaranthus tricolor	*Amaranthus tricolor*	AAB67746	Protein
	Atriplex patens	*Atriplex patens*	ABJ90432	Protein

	Beta vulgaris	*Beta vulgaris*	AAS67266	Protein
	Bougainvillea spectabilis	*Bougainvillea spectabilis I*	AF445416	mRNA
		Bougainvillea spectabilis II	ABI64066	Protein
	Chenopodium album	*Chenopodium album*	AAK28323	WGS
	Dianthus caryophyllus	*Dianthus caryophyllus*	X59260	mRNA
	Dianthus chinensis	*Dianthus chinensis*	AF219236	mRNA
	Mesembryanthemum crystallinum	*Mesembryanthemum crystallinum*	AAB96824	Protein
	Mirabilis expansa	*Mirabilis expansa*	AY148091	mRNA
	Mirabilis jalapa	*Mirabilis jalapa*	D10227	mRNA
	Phytolacca americana	*Phytolacca americana*	AY547315	DNA
	Saponaria officinalis	*Saponaria officinalis*	AM748700	mRNA
	Silene latifolia	*Silene latifolia*	P85101	Protein
	Spinacia oleracea	*Spinacia oleracea*	AB070925	mRNA
	Stellaria media	*Stellaria media*	ADB83313	Protein
	Ximenia americana	*Ximenia americana*	CAJ38823	Protein
	Camellia sinensis	*Camellia sinensis*	ADF45510	Protein
	Volkameria inermis	*Volkameria inermis*	EU839992	mRNA
	Centaurea solstitialis	*Centaurea solstitialis*	EH756893	mRNA
	Helianthus tuberosus	*Helianthus tuberosus*	EL455240	EST
	Parthenium argentatum	*Parthenium argentatum*	GW777166	mRNA
	Cannabis sativa	*Cannabis sativa I*	AGQN01263163	WGS
		Cannabis sativa II	AGQN01148610	WGS
		Cannabis sativa III	AGQN01213101	WGS
		Cannabis sativa IV	AGQN01137446	WGS
		Cannabis sativa V	AGQN01137447	WGS
	Malus Domestica	*Malus Domestica*	ACYM01011001	WGS
	Gossypium raimondii	*Gossypium raimondii*	ALYE01008201	WGS
	Theobroma cacao	*Theobroma cacao I*	CACC01024922	WGS
		Theobroma cacao II	CACC0102492	WGS
		Theobroma cacao III	CACC01008026	WGS

Supplementary Table 3. Integrative analyses of substitution rates by SLAC, FEL and REL tests. Those codons under purifying selection for each test are presented in violet. These results were estimated using a p-value < 0.01 for SLAC and FEL tests, and a Bayes factor cutoff= 50 for REL test. No codons were found under positive selection. Active site codons are indicated in yellow.

Codon	SLAC dN-dS	SLAC p-value	FEL dN-dS	FEL p-value	REL dN-dS	REL Bayes Factor
1	0.000	0.000	0.000	1.000	-0.930	3.542.440
9	-2.072	0.043	-2.006	0.001	-1.461	84.762
23	-1.815	0.005	-1.468	0.000	-1.114	7.982.030
30	-1.216	0.039	-483.270	0.000	-1.102	477.019
33	-0.605	0.165	-0.172	0.054	-0.836	703.730
34	-0.405	0.247	-0.120	0.078	-0.764	219.770
37	-1.335	0.007	-0.806	0.000	-0.920	2.974.950
46	-1.557	0.027	-0.890	0.007	-0.856	86.225
48	-1.517	0.052	-1.237	0.007	-0.941	59.976
49	-1.485	0.012	-1.696	0.000	-1.178	1.589.890
53	-1.199	0.012	-0.408	0.002	-0.865	473.736
55	-0.902	0.112	-0.546	0.026	-0.895	162.204
59	-0.900	0.037	-0.439	0.003	-0.867	906.730
61	-0.300	0.333	-0.073	0.121	-0.575	105.687
62	-1.401	0.031	-1.330	0.003	-1.028	393.040
66	-0.502	0.215	-0.256	0.039	-0.848	500.612
68	-1.549	0.013	-1.407	0.001	-1.089	652.738
79	-1.153	0.015	-0.526	0.001	-0.884	775.776
82	-1.787	0.005	-2.016	0.000	-1.146	4.806.020
84	-0.605	0.198	-0.172	0.068	-0.827	147.173
85	-1.134	0.018	-0.442	0.002	-0.890	945.605
87	-0.900	0.042	-0.240	0.009	-0.816	224.194
91	-1.258	0.047	-0.836	0.004	-0.913	255.985
97	-1.688	0.016	-3.395	0.000	-1.357	951.017
120	-1.170	0.076	-1.137	0.007	-0.928	84.017
131	-0.984	0.110	-5.106	0.000	-2.881	528.198
135	-1.207	0.038	-0.600	0.008	-0.871	268.034
137	-1.707	0.009	-0.654	0.001	-0.918	976.519
140	-1.628	0.004	-0.589	0.000	-0.907	2.990.570
151	-1.782	0.026	-1.153	0.006	-0.900	43.879
153	-1.377	0.033	-2.082	0.001	-1.264	727.823
155	-1.354	0.033	-1.182	0.002	-0.998	214.374
157	-1.451	0.020	-0.945	0.001	-0.948	575.293
158	-1.065	0.025	-0.661	0.001	-0.890	555.446
161	-0.816	0.050	-0.316	0.007	-0.839	219.591
165	-1.676	0.006	-1.468	0.000	-1.211	2.108.320
166	-0.585	0.171	-0.198	0.046	-0.816	400.256
169	-0.502	0.215	-0.204	0.049	-0.839	429.773
175	-1.604	0.070	-1.654.870	0.001	-3.108	191.650
178	-0.786	0.117	-0.678	0.003	-0.888	73.938
180	-1.199	0.012	-0.602	0.000	-0.901	4.823.560
181	-0.557	0.179	-0.149	0.064	-0.791	388.112
185	-1.757	0.005	-2.482	0.000	-1.103	6.544.390
187	-1.075	0.039	-0.313	0.009	-0.848	573.723
188	-1.199	0.012	-0.615	0.000	-0.914	2.766.090
190	-1.419	0.006	-0.777	0.000	-0.928	8.231.280
191	-1.178	0.029	-0.417	0.005	-0.880	1.466.510
192	-1.049	0.045	-0.702	0.003	-0.880	546.106
193	-1.349	0.018	-0.798	0.002	-0.871	349.318
194	-1.022	0.100	-1.883	0.001	-1.346	146.140
207	-1.499	0.004	-0.877	0.000	-0.957	11.951.900
213	-1.081	0.090	-2.813	0.002	-1.219	82.171
215	-1.585	0.019	-0.686	0.007	-0.906	341.278
221	-2.495	0.007	-2.569	0.001	-1.455	251.986
222	-1.783	0.023	-2.166	0.002	-1.559	216.213
225	-1.199	0.012	-0.414	0.001	-0.883	1.765.240
228	-1.825	0.013	-2.446	0.001	-2.118	433.485
233	-2.131	0.037	-0.972	0.000	-0.989	829.644
239	-1.237	0.051	-2.492	0.001	-1.833	672.254
243	0.000	0.000	0.000	1.000	-0.917	228.045
244	-1.468	0.013	-3.293	0.000	-1.342	1.222.570
246	-1.476	0.012	-1.274	0.001	-1.101	769.780
247	-1.199	0.017	-0.808	0.000	-0.970	2.253.410
269	-1.420	0.111	-0.766	0.001	-0.933	966.777
274	-1.324	0.019	-0.790	0.003	-0.914	616.562
288	-0.508	0.213	-0.195	0.049	-0.820	380.593
295	-0.903	0.043	-0.325	0.007	-0.837	52.799
299	-1.091	0.100	-0.948	0.006	-0.938	183.184
313	-1.449	0.006	-0.698	0.000	-0.911	2.645.140
315	-1.072	0.041	-0.307	0.009	-0.863	777.034
316	-1.519	0.007	-1.172	0.000	-1.118	8.088.370
322	-1.291	0.116	-2.786	0.007	-1.338	24.264
345	-1.846	0.034	-1.601	0.009	-0.972	31.907
365	-0.564	0.177	-0.131	0.072	-0.763	213.515
368	-1.199	0.014	-0.569	0.001	-0.883	1.708.740
370	-0.581	0.200	-0.443	0.004	-0.854	99.841
372	-1.538	0.004	-0.782	0.000	-0.916	1.403.450
374	-1.234	0.041	-0.865	0.004	-0.874	214.831
378	-0.915	0.073	-0.785	0.007	-0.882	146.391
380	-1.012	0.050	-0.434	0.014	-0.819	113.302
401	-0.900	0.037	-0.309	0.004	-0.851	588.744
403	-0.974	0.126	-2.015	0.003	-1.224	70.505
412	-1.138	0.018	-0.436	0.002	-0.897	1.059.120
415	-1.011	0.096	-0.321	0.058	-0.838	120.470
419	-2.660	0.032	-0.662	0.004	-0.927	770.996
429	-2.170	0.007	-4.440	0.000	-1.589	950.750
430	-1.078	0.038	-0.277	0.011	-0.846	536.350
431	-1.105	0.110	-11.486	0.002	-1.226	24.637
435	-0.839	0.087	-1.752	0.007	-1.001	89.806
436	-1.637	0.011	-0.804	0.001	-0.973	1.717.340
443	-0.871	0.115	-0.943	0.010	-0.873	52.265
458	-1.344	0.033	-1.325	0.001	-1.084	464.844
473	-1.271	0.048	-1.339	0.004	-0.976	129.196
499	-0.602	0.195	-0.154	0.065	-0.790	250.543
500	-2.521	0.001	-3.285	0.000	-1.580	10.402.000

Supplementary Table 4. List of primer sequences and annealing temperatures (Ta).

Primer Names	Primer sequences	Ta (°C)
Fw RIPcu	ATGCAGTTCAACCGCGC	64°C
Rev RIPcu	TTAGAAGCAATTCTTTGCGAAAAGTTC	64°C
Fw intergen	GCAGAGGATTGAGAAGAGAAAGG	60°C
Rev intergen	CCGTTTATGGAGAACTGGAGAAG	60°C
