# Supplementary material for: Metazoan Ribosome Inactivating Protein encoding genes acquired by Horizontal Gene Transfer
Source: Sci Rep. 2017 May 12;7:1863. doi: 10.1038/s41598-017-01859-1 (PMC5431988; doi:10.1038/s41598-017-01859-1)
Supplement: Supplementary file 2 — Supplementary information 2 [file 41598_2017_1859_MOESM2_ESM.pdf]

Metazoan Ribosome Inactivating Protein encoding genes acquired by Horizontal Gene Transfer

Walter J. Lapadula[1*], Paula L. Marcet[2], María L. Mascotti[1], María V. Sánchez Puerta[3], Maximiliano Juri Ayub[1*]

1. Instituto Multidisciplinario de Investigaciones Biológicas de San Luis, IMIBIO-SL-CONICET and Facultad de Química, Bioquímica y Farmacia, Universidad Nacional de San Luis, San Luis Argentina.

2. Centers for Disease Control and Prevention, Division of Parasitic Diseases and Malaria, Atlanta, USA.

3. Instituto de Ciencias Básicas, IBAM-CONICET and Facultad de Ciencias Agrarias, Universidad Nacional de Cuyo, Mendoza, Argentina.

*Corresponding authors: mjuriayub@hotmail.com, wlapadula@gmail.com

Supplementary Information

Supplementary Date 1. Evolution of RIP genes in metazoans

Table 1. Species of mosquitoes harboring RIP genes are indicated in the first column. RIP names used in this manuscript file are indicated in the second column. GenBank accession numbers of proteins, contigs and transcriptomic data are presented in the third, fourth and fifth columns, respectively.

Organism	RIP names	Protein GenBank	Contig GenBank	Transcriptomic GenBank
Culex quinquefasciatus	RIPcu	XP_001850873	AAWU0105132	FF167149
Culex molestus	RIPmo	ANZ90790		
Culex pipiens	RIPpi	ANZ90791		
Culex torrentium	RIPto	ANZ90792		
Aedes aegypti	RIPaeI	XP_001650164	AAGE02007824	DV375198
	RIPaeI LIKE		AAGE02007824	EE993988
	RIPaeII	XP_001658853	AAGE02013700	DV312220
Aedes albopictus	RIPalI	KXJ78155	JXUM01048494	
	RIPalII	KXJ78156	JXUM01048494	
	RIPalIII	KXJ78157	JXUM01048511	GCLM01024725
	RIPalIV	KXJ78158	JXUM01048511	
	RIPalV	KXJ73764	JXUM01085134	
	RIPalVI	KXJ73133	JXUM01090901	GCLM01024726
	RIPalVII	KXJ73132	JXUM01090901	

With the aim of better understanding the evolution of metazoan RIP sequences, their evolutionary history was investigated (Fig 1). Additionally, the reconstruction of the ancestral sequences of dipterous RIPs was carried out in order to analyze the GC content at each node. BaseML algorithm from the PAML package was implemented [1]. Crude MSA and ML tree (constructed using PhyML [2]) were used as input. Alignment parameters were calculated using JModelTest 2.1.7 following the AIC criterion. 13 sequences were reconstructed with an accuracy ranging from 0.66585 to 0.96780 for a site. The GC content was investigated for each node state (Fig 1).

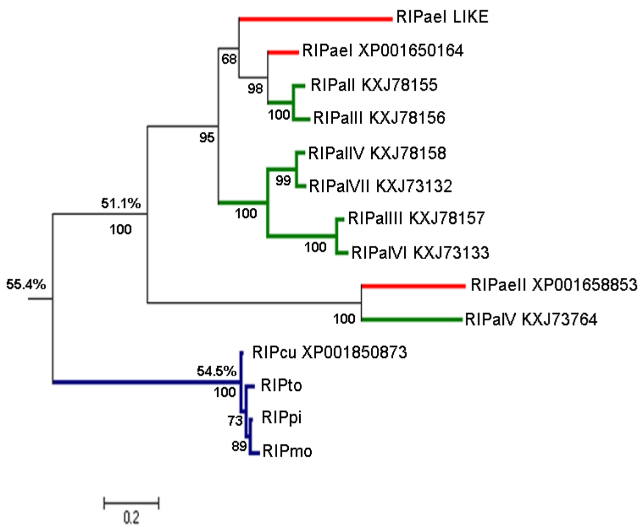

Fig 1: Maximum likelihood tree of dipterous RIPs. Numbers below branches indicate Bootstrap values (BS). The clade of *Culex* species is indicated with blue branches. RIPs of *Ae. aegypti* and *Ae. albopictus* are indicated with red and green branches, respectively. Numbers above branches inferred GC content in ancestral sequences.

According to the topology of this tree, it seems possible that multiple duplication and loss events of paralogous genes took place, yielding heterogeneity in the number of RIP genes among Culicinae species. In *Culex* species only one orthologous gene exists while in *Ae. albopictus* there are seven genes, six of which result of three specific duplication events (RIPalI/RIPalII, RIPalIV/RIPalVII and RIPalIII/RIPalVI). The higher number of RIPs found in *Ae. albopictus* could be explained by the genomic expansion occurred in this species, which has one of the largest genomes in Diptera [3]. Regarding the losses, the ortholog of RIPaeI LIKE is absent in *Ae. albopictus* while the ortholog of the clade constituted by RIPalIV, RIPalVII, RIPalIII and RIPalVI is missing in *Ae. aegypti*.

The orthologous relationships observed between *Ae. aegypti* and *Ae. albopictus* RIPs is supported by the result of performing reciprocal BLAST searches (Table 1) and the genomic context analyses (Fig 2).

Table 2. (A) BLASTp results using RIPs protein sequences of *Ae. aegypti* as queries and the non-redundant protein database of *Ae. albopictus*. **(B)** tBLASTn results using RIP protein sequences of *Ae. albopictus* as queries and the Whole Genome Sequence database from *Ae. aegypti.*

A) *Ae. aegypti vs Ae. albopictus*

	e value	identity	GenBank
**RIPaeI**	0.0	80%	KXJ78156.1 RIPal II
	0.0	79%	KXJ78155.1 RIPal I
	0.0	64%	KXJ78158.1 RIPal IV
	0.0	62%	KXJ73132.1 RIPal VII
	0.0	58%	KXJ78157.1 RIPal III
	0.0	58%	KXJ73133.1 RIPal VI
	2,00E-69	37%	KXJ73764.1 RIPal V
**RIP like**	5,00E-176	55%	KXJ78156.1 RIPal II
	6,00E-174	55%	KXJ78155.1 RIPal I
	2,00E-157	51%	KXJ78158.1 RIPal IV
	2,00E-151	48%	KXJ73133.1 RIPal VI
	4,00E-151	50%	KXJ73132.1 RIPal VII
	1,00E-148	47%	KXJ78157.1 RIPal III
	7,00E-65	36%	KXJ73764.1 RIPal V
**RIP aeII**	6,00E-157	57%	KXJ73764.1 RIPal V
	4,00E-78	36%	KXJ78156.1 RIPal II
	5,00E-78	37%	KXJ73132.1 RIPal VII
	2,00E-77	35%	KXJ78155.1 RIPal I
	9,00E-77	37%	KXJ78158.1 RIPal IV

B) *Ae. albopictus vs Ae. aegypti*

	e value	identity	GenBank
**KXJ78155.1 RIPal I**	0	80%	RIPaeI
	1xe-157	55%	RIP Like
	6xe-71	36%	RIPaeII
**KXJ78156.1 RIPal II**	0	78%	RIPaeI
	1xe-149	55%	RIP LIKE
	3xe-66	35%	RIPaeII
**KXJ78157.1 RIPal III**	0	64%	RIPaeI
	2xe-69	37%	RIPaeII
**KXJ78158.1 RIPal IV**	0	64%	RIPaeI
	3xe-142	51%	RIP LIKE
	2xe-69	37%	RIPaeII
**KXJ73764.1 RIPal V**	4XE-132	57%	RIPaeII
	9xe-57	37%	RIPaeI
	5xe-55	35%	RIP Like
	2xe-136	50%	RIP LIKE
	1xe-70	37%	RIPaeII
**KXJ73132.1 RIPal VII**	0	62%	RIPaeI
	2xe-136	68%	RIP LIKE
	1xe-70	37%	RIPaeII

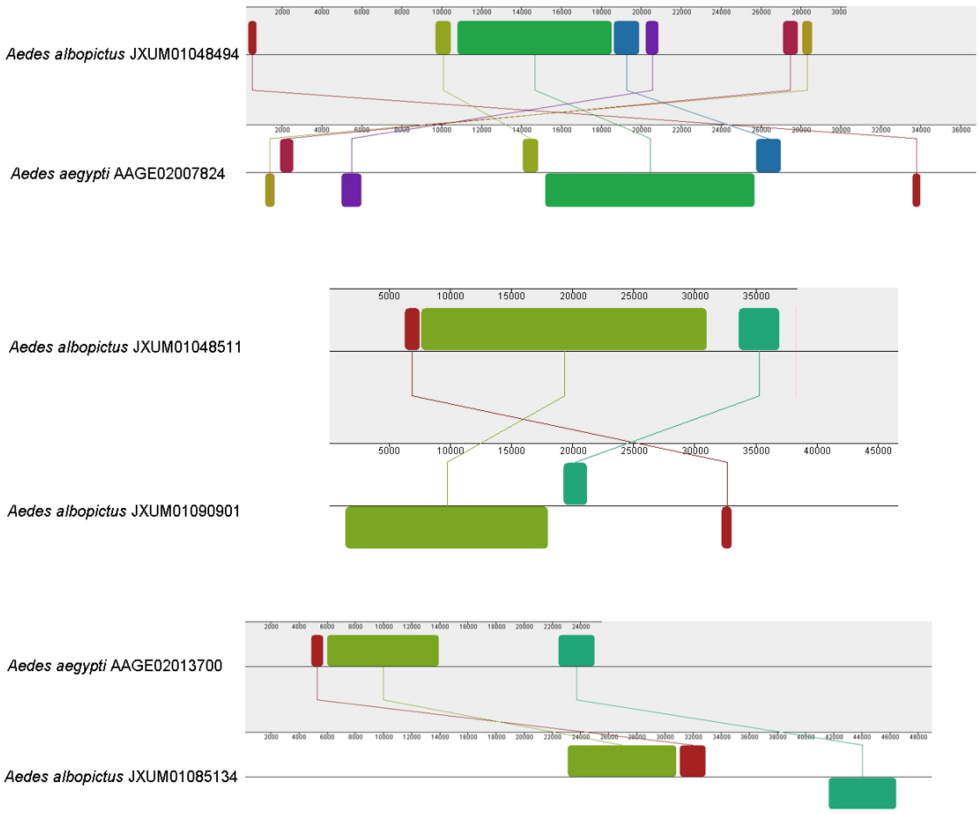

Fig 2: Genomic context analyses. Contigs containing RIP genes in *Ae. aegypti* and *Ae. albopictus* were compared in order to analyze the presence of conserved synteny blocks by using the Mauve software [4].

References

1. Yang Z (2007) PAML 4: phylogenetic analysis by maximum likelihood. Molecular biology and evolution 24: 1586-1591.
2. Guindon S, Gascuel O (2003) A simple, fast, and accurate algorithm to estimate large phylogenies by maximum likelihood. Systematic biology 52: 696-704.
3. Chen X-G, Jiang X, Gu J, Xu M, Wu Y, et al. (2015) Genome sequence of the Asian Tiger mosquito, Aedes albopictus, reveals insights into its biology, genetics, and evolution. Proceedings of the National Academy of Sciences 112: E5907-E5915.
4. Darling AC, Mau B, Blattner FR, Perna NT (2004) Mauve: multiple alignment of conserved genomic sequence with rearrangements. Genome research 14: 1394-1403.

Supplementary Data File 2. Alternative hypotheses explaining the presence of RIP genes in Metazoa

---

In many cases, misinterpretation or overestimation of HGT events is caused by not considering the alternative explanation of hidden paralogy [1]. In this context, two alternative hypotheses are possible to explain the presence of RIP genes in mosquitoes' genomes:

(i) Vertical inheritance with the occurrence of independent, multiple gene losses.

(ii) HGT of a RIP gene to the common ancestor of the genera *Culex* and *Aedes*.

These two alternative scenarios are graphically represented as follows (Fig 1).

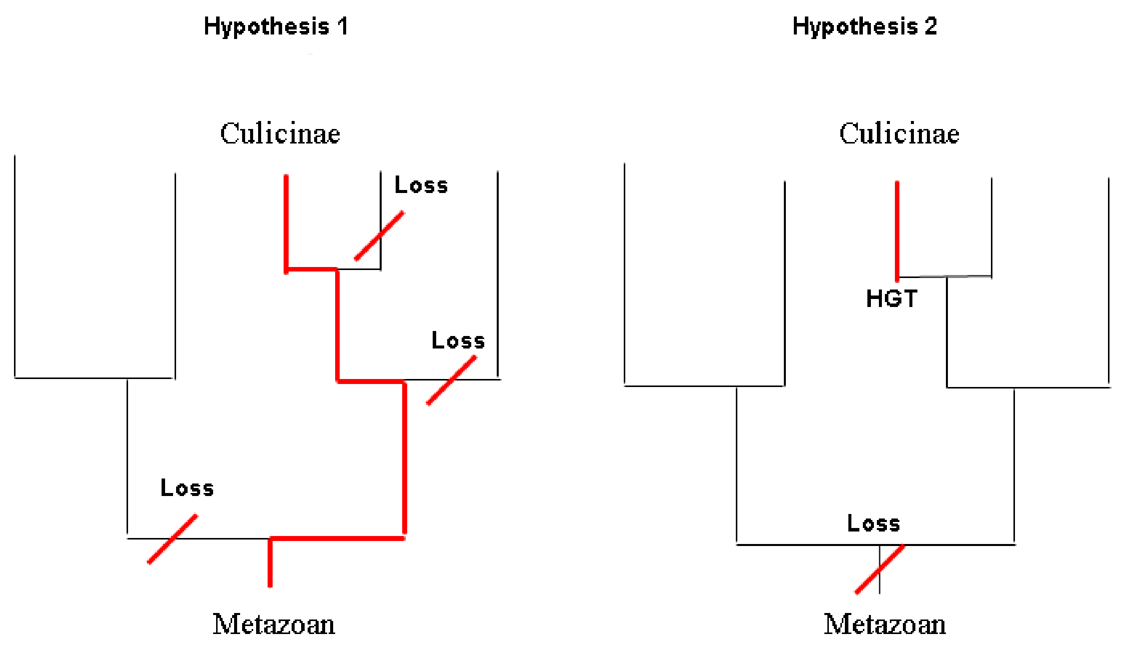

Fig 1: Schematic representation of two alternative hypotheses explaining the origin and evolution of RIP genes in Diptera. In **hypothesis 1** the presence of RIP genes along the organism's evolution is indicated in red color. In **hypothesis 2** the origin of RIP genes in metazoan is consequence of one HGT event in the ancestor of Culicinae. Gene losses are indicated as crossed red bars on branches.

To compare the likelihood of each hypothesis, we estimated the minimal number of losses required on the phylogeny of Bilateria lineage under hypothesis 1. To do this, a loss event was only considered when no RIP genes were detected in species with fully-sequenced genomes (Fig 2). The Assembly tool available at NCBI was used to get the genome sequencing project state for different organisms. All lineages with full genome representation at different assembly levels (complete genomes, chromosomes, scaffolds and contigs) were listed. Then, exhaustive searches using BLAST were conducted against the listed genomes; previously reported RIP sequences, including metazoan

RIPs, were employed as queries [2]. As a complementary searching strategy a MSA of mosquito RIPs was constructed and this was used in HMMER searches, employing the program hmmsearch [3].

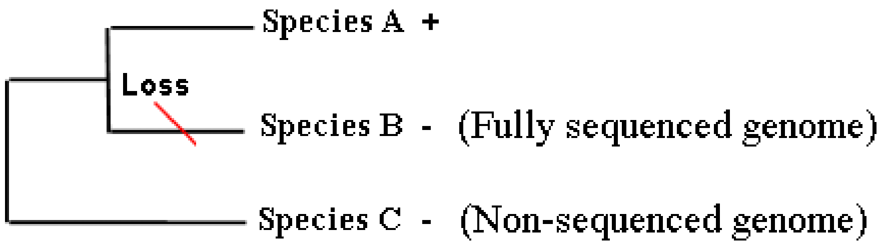

Fig 2: Schematic representation of gene losses counting methodology. The presence or absence of RIP genes is represented with (+) or (–), respectively. A loss event, in an organism with fully sequenced genome is indicated with a red crossed line on the branch.

Counting by a conservative method (*i.e*. considering only one gene loss event when polytomic branches are found) the number of necessary losses rises up to 15. If a more relaxed counting method is considered, the number of losses equals 23 (Fig 3). In the scenario involving a HGT event, the number of losses is reduced to one (the loss of a RIP encoding gene in the ancestor of all metazoans). Therefore, the hypothesis (i) becomes a less parsimonious alternative compared to the lateral transfer of a RIP gene from a donor to the ancestor of *Culex* and *Aedes* (hypothesis ii in the main text).

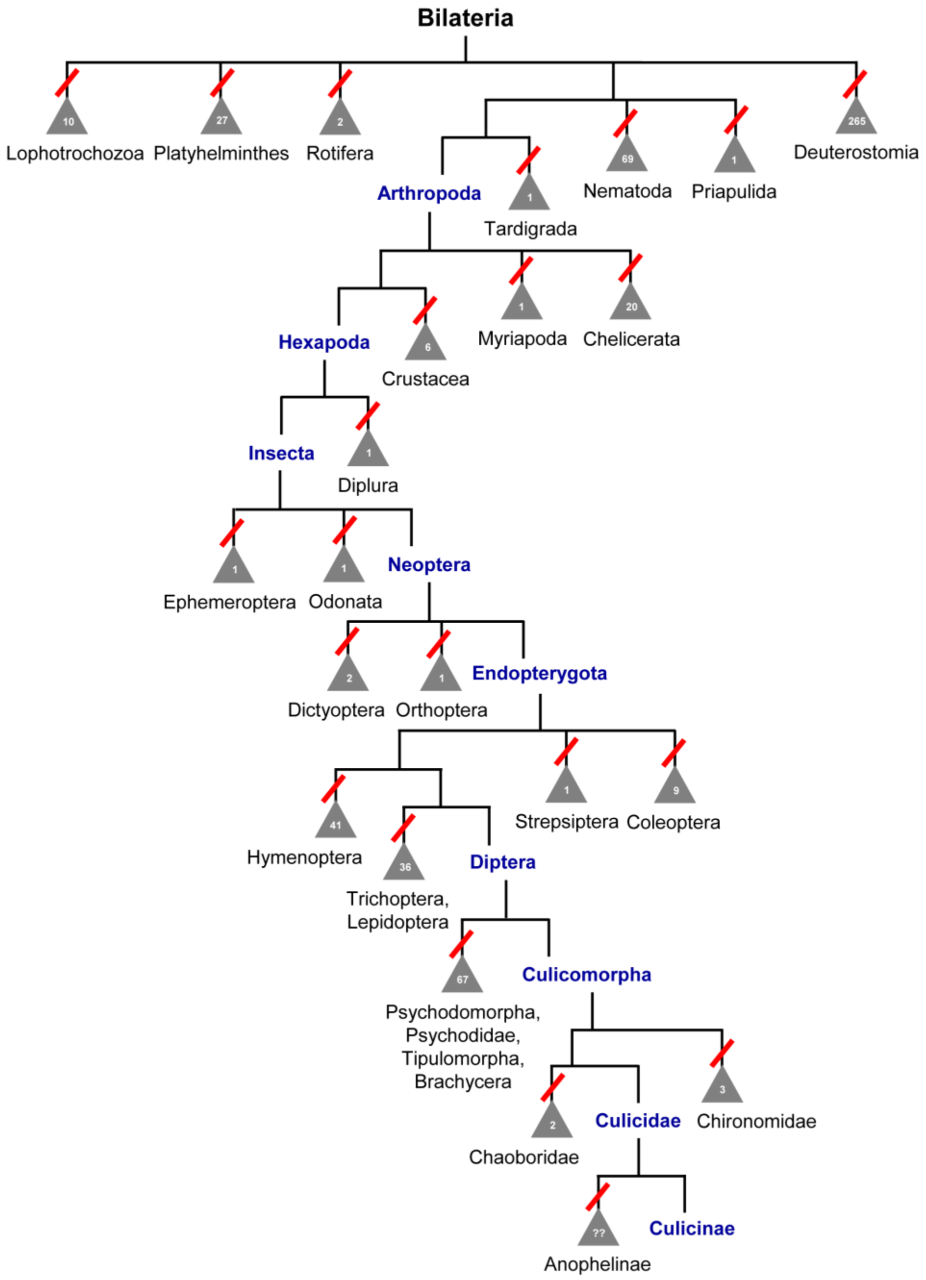

Fig 3: Schematic representation of RIP encoding genes in Bilateria genealogy. Taxa with at least one fully-sequenced genome are presented. Taxa where RIP genes are present are written in blue. Losses are represented as red crossed bars on branches. The number of available genomes for each taxon is shown inside the triangles. The phylogeny was based on the Tree of life webserver (http://tolweb.org/tree/).

References

1. Huang J (2013) Horizontal gene transfer in eukaryotes: the weak-link model. Bioessays 35: 868-875.
2. Lapadula WJ, Sanchez Puerta MV, Juri Ayub M (2013) Revising the taxonomic distribution, origin and evolution of ribosome inactivating protein genes. PLoS One 8: e72825.
3. Finn RD, Clements J, Eddy SR (2011) HMMER web server: interactive sequence similarity searching. Nucleic Acids Res 39: W29-37.

Supplementary Data File 3. Alignments and Trees

Sequence alignment Figure 2

>EF441598

YVDSLNVIRSAIGTPL--Q--TIS-------SGGTS-----LLMID----S-------GS

-----GD----NLFAVDVRGIDPEEGRF---NN-----L----RLIVERNNLYVTGF-VN

------RTNNV------FYRFA-----D-------------F-SHVTF------------

------------------PG----------TTA-VTLSGDS-SYTT----L--QR-VAG-

-------IS-RTGMQI-NRHSLTTSYLDLMSH----------------------------

---SGTSLTQSVARAMLRFVTVTAEALRFRQIQRGFR-TTLDDLS---------------

-GRSYVM--------------TAEDVDLTLNWGRLS

>AF500192

YVSSLNSIRTEISTPL--E--HIS-------QGTTS-----VSVIN----H-------TP

-----PG----SYFAVDIRGLDVYQARF---DH-----L----RLIIEQNNLYVAGF-VN

------TATNT------FYRFS-----D-------------F-THISV------------

------------------PG----------VTT-VSMTTDS-SYTT----L--QR-VAA-

-------LE-RSGMQI-SRHSLVSSYLALMEF----------------------------

---SGNTMTRDASRAVLRFVTVTAEALRFRQIQREFR-QALSET----------------

-APVYTM--------------TPGDVDLTLNWGRIS

>ZP02062680

YVDSLNKIRERIGIRLPNL--TTS-------QGNMS---ISVFVLS----P-------SA

-----SN----IGIIVTLQGIDYDDPNT---SV---P-I----RLVLSPENLYLAGF-I-

-------QGNT------FYRFR-----D-------------R-QNTVL------------

------------------PSDI-------HVQI-VDLTTGS-DYTE----L--ER-VGD-

-------VR-RDRLQI-NRHSLVTSYRDLNQF----------------------------

---SGTALNQASARAMLRFITVLPEALRFRQIQRNFR-PVLSQTAS--------------

-NSHYIM--------------SPSNISLTLNWGGLS

>WP034895486

YVKSLKDIRGNIGVPL------TN-------IGGDQ---ARIFMLP----P--QR---EG

-----GM----DGIIIAIRGVDFYNDEE---VA---P-V----NFVLDPTNLYISGF-I-

-------VNNI------YYRFS-----D-------------T-SGINA------------

------------------PG---------VIGT-IQINQES-SYTS----L--QR-VAN-

-------MQ-RSDMII-NRATLTDGYSQLTRF----------------------------

---SGGNINQETARALLRYITVIPEALRFRQIQRNFR-PALDVA----------------

-ATQYVM--------------SNPDISLTLNWGRLS

>ZP06230219

YVQSLGAIRAAMGDAM-----SLTNI-----PGNKI-----LYQLR----P-------DA

-----SN--IVEGVTIEIIGVGRNNSPS---NR---D-V----RFVINPSDLYLTGF-I-

-------VGRI------FYRFS-----D-------------F-SDTAS------------

-------GRVQVNA----PRHL-------VDFT-IDMTVDS-SYLS----L--AR-SAG-

-----VSAD-RTDLSI-DRYSLMKGYRDLINHV---------------------------

--SSTRTINGAEARALLSYATVLSEAVRFRSIQGNFA-STALGDDA--------------

-FTPYRL--------------SLEDSNRTTRWDRLS

>WP029217990

YAKSISDFRRGIGTEL------TR-------IGN-------VYETS----P-------VG

-----------DLITVSL-----SND-R---GR---Q-I----RLVLRPRDLYVIGY---

------EAYGI------FYRFN-----D--------------------------------

-------PEYQSLAL---PY--------AATTQ-LPYNMYS-TYDS----L--SS-FGD-

-------IQ-LSDVNV-SRASVYGAIDTLSRF----------------------------

--GDGSVNANSLSRALYPLIIAVSEGARFRPVSSAIV-TALGSSTAMT------------

---------------------LGRNTGLVRNWQQIS

>WP015197802

YIRAVSSIRNSQTELV----NLNI-------PG--------SSTNP----P--QIRRTRN

---QADN----VYFTVQV-----EFE-----GN---N-I----GFVFRRRDLYLQGY-IN

------SRNST------YYYFR-----E-------------A------------------

--------TVTSVRQ--------------ANSQ-TQLSATE-NYNS----L-----LGGE

----------FSRADF-NLDELRQSFRNLVNN----------------------------

---NPSRNSQSINTALVRFAVAISEAVRFNEVGRNVA-RLFRNPSARINF----------

---------------------EETHDTVLSNWSRYS

>KIE12545

Y---IRALRDLINTQS-----QIVTVDGLLETGGD------IEV------P--AT---YN

-----DS----NYFIVQV-----EFD-----NQ---S-I----GFVIRRQNLYVDGFIRN

YN--PDRRTGT------YYYVR-------------------N-ERN--------------

------TTPDNRVVS---PEDMA----GEVSNS-IELGYGG-RYGD----FFANDYSRG-

----------RQAQIF-GLEDLRQDFRNLVNA----------------------------

-----NPNSQGVQNAFAHFVIIISEAMRLDPVYDRVV-NLFQNPDARIDF----------

---------------------RAIHNEYLSRWSDHS

>EEP74218

YEDLITLIRVQNQ---------LS-------AN--------TPVTP---APTASD---AD

-----PA----GYFSVAL-----VAG-----GR---R-I----ELVIRRDDLYLVGW-YQ

ADQDRAGDADV------YFRFR-------------------H-DR---------------

------------------SGSPYDFRRTTRTRI-VDLTFTG-SYLD----L--GY-PA--

-------EK-RKALNL-GGGALIKALQLLS------------------------------

-----GTNGQQPNEALVVTIQMVLEAARFHPLFEHLR-EHWDE-WG--------------

-----AP--------------PPALVDLQNNWGDLS

>M77122

YSAFIASVRKDVIKHC-----TDH-------KG------IFQPVLP----P--EK---KV

-----PE----LWFYTEL-----KTR-----TS---S-I----TLAIRMDNLYLVGF-RT

-------PGGV------WWEFG-------------------K-DGD--------------

----------THLLG---DN------------P-RWLGFGG-RYQD----L--IG-NKG-

----------LETVTM-GRAEMTRAVNDLAKKKKMATL----EEEEVKMQMQMPEAADL-

AAAAAADPQADTKSKLVKLVVMVCEGLRFNTVSRTVD-AGFNS-----------------

-QHGVTL--------------TVTQGKQVQKWDRIS

>EU725161

YSAFIASVRKDVIKHC-----TDH-------KG------IFQPVLP----P--EK---KV

-----PE----LWFYTEL-----KTR-----TS---S-I----TLAIRMDNLYLVGF-RT

-------PGGV------WWEFG-------------------K-AGD--------------

----------THLLG---DN------------P-RWLGFGG-RYQD----L--IG-NKG-

----------LETVTM-GRAEMTRAVNDLAKKKKMATL----EEEEVQMQMQMPEAADL-

AAAAAADPQADTKSKLVKLVVMVCEGLRFNTVSRTVD-AGFNS-----------------

-QHGVTL--------------TVTQGKQVQKWDRIS

>BT037982

YSAFIASVRKDVIKHC-----TDH-------KG------IFQPVLP----P--EK---KV

-----PE----LWFYTEL-----KTR-----TS---S-I----TLAIRMDNLYLVGF-RT

-------PGGV------WWEFG-------------------K-DGD--------------

----------THLLG---DN------------P-RWLGFGG-RYQD----L--IG-NKG-

----------LETVTM-GRAEMTRAVNDLAKKKKMATL----EEEEVQMQMQMPEAADL-

AAAAAADPQAGTKSKLVKLVVMVCEGLRFNTVSRTVD-AGFNS-----------------

-QHGVTL--------------TVTQGKQVQKWDRIS

>DQ147068

YSAFIASVRKDVIKHC-----TDH-------KG------IFQPVLP----P--EK---KV

-----PE----LWFYTEL-----KTR-----TS---S-I----TLAIRMDNLYLVGF-RT

-------PGGV------WWEFG-------------------K-DGD--------------

----------THLLG---DN------------P-RWLGFGG-RYQD----L--IG-NKG-

----------LETVTM-GRAEMTRAVNDLAKKKKMATL-----EEEVQMQMQMPEAADLA

AAAAAADPQADTKSKLVKLVVMVCEGLRFNTVSRTVD-AGFNS-----------------

-QHGVTL--------------TVTQGKQVQKWDRIS

>DQ147051

YSAFITSVRKDVIKYC-----TNH-------TG------IVQPVLP----L--EK---KV

-----PE----LWFYTEL-----KTK-----TR---S-I----TLAIRMDNLYLVGF-KT

-------PGGV------WWEFG-------------------K-DGD--------------

----------THLLD---DN------------A-KWLGFGG-RYQD----L--IG-SKG-

----------LETVTM-GRAEMTTAVNYLAKK-TTTLAEAAAEEEE-L------------

LLQAAADPKADEKSNLAKLVIMVCEGLRFFTVSRKVD-EGFKK-----------------

-PQAVTI--------------SALEGKQVQK-----

>L26305

YSAFITSVRKEVIKYC-----TNH-------TG------IVQPVLP----L--EK---NV

-----PE----LWFYTEL-----KTK-----TR---S-I----TLAIRMDNLYLVGF-RT

-------PGGV------WWEFG-------------------K-DGD--------------

----------THLLD---DN------------A-KWLGFGG-RYQD----L--IG-SKG-

----------LETVTM-GRAEMTTAVNYLAKKTTTTLAEAAEEEEELL------------

LLQAAADPKAEEKSNLAKLVIMVCEGLRFFTVSRKVD-EGFKK-----------------

-PQAVTI--------------SALEGKQVQKWDRIS

>Fl902192

YGAFIAAVRKQVTKYC-----TDR-------TG------VLQPVLP----P--EK---KV

-----PE----LWFYTEL-----KTK-----TS---S-I----TLAIRMDNLYLVGF-RT

-------PAGV------WWEFG-------------------K-EGG--------------

----------THLLY---DN------------P-RWLGFGD-KYKD----L--IG-DNG-

----------LETVAM-GRQEMTGAVNYLAKKTMTL------EEEEQL------------

LQAAAADPQADAKNSLVKLVIMVCEGLRFWTVSRKVD-EGFNN-----------------

-KQEVKL--------------TAMQGKQVQKWDRIS

>EMT05877

YGDFIRIVRHHVIDYC-----SDKRG-----DG------IVQPVLP----P--EK---AV

-----PS----SWFHVVLG----NTP-----AS---S-V----TLAIRMDNLYLVGF-RT

-------PAGV------WWEFG-------------------K-EGG--------------

----------ARLI----AG------------S-TWLGFGG-RYQD----L--IG-QKG-

----------LDAVTL-GRAQMAAAVDVLAKHGTTNAL----EEEEEE------------

-EGVHADPYAVPKTMLAKLVIMVCEGLRFLTVSGRVD-REFDM-----------------

--AEVTI--------------TEIEGKQVNKWDRIS

>XP004975022

YGDFIRIVRQNVIKYC-----SDRR------PK------VVQPVLP----P--EQ---RV

-----PR----LWFHVVL-----RTR-----TS---S-L----TLAVRVDNLYLVGF-KT

-----PGPAGL------WWEFN-------------------N-EHN--------------

----------THLI----PN------------S-NWLGFGG-RYQD----L--VG-QKG-

----------LETVAL-GRAGMTAAVDVLAKHDTTTAL----EEHQQR------------

LGAHQADPYALPKSMLVKLVIMVCEGVRFHTVYGTVD-REFNT-----------------

--AVAKI--------------TEMDGKQVNKWDRIS

>AB051107

YGDFIAGIRSRVANPR-----HFS-------RN--------RPVLP----PVEPP---PP

-----PR----RWFHVVL-----RAS-P---TA---A-L----TLATRADNLYLEGF-RS

-------SDGR------WWELT---------------------------------------

----------PGILG-AAPG---------GAAA-TYVGFGG-SYRD----L--LG-DTDR

----------LTVVTL-GPQQMAQAVNALAARRPAD------------------------

--LANGAAQRRAMDAVAALLLMVHEATRFQTVSRLVA-GLMHPKAA--------------

-SKSGAI--------------TTAMRKQVNGWQVLS

>D13795

YVTFINGIRNKLRNPG-----HSS-------HN--------RPVLP----PIEPN---VP

-----PS----RWFHIVL-----KTS-P---AS---TGL----TLATRADNLYWEGF-KS

-------SDGT------WWELT---------------------------------------

----------PGLI----PG------------A-THVGFGG-TYRD----L--LG-DTDK

----------LTNVAL-GRQQMADAVTALYGRTKAD------------------------

--KTSGPKQQQAREAVTTLLLMVHEATRFQTVSGFVA-GVLHPK----------------

EKKSGKI--------------GNEMKAQVNGWQDLS

>M62905

YATFIAGIRNKLRNPA-----HFS-------HN--------RPVLP----PVEPN---VP

-----PS----RWFHVVL-----KAS-P---TS---AGL----TLAIRADNIYLEGF-KS

-------SDGT------WWELT---------------------------------------

----------PGLI----PG------------A-TYVGFGG-TYRD----L--LG-DTDK

----------LTNVAL-GRQQLADAVTALHGRTKAD------------------------

--KPSGPKQQQAREAVTTLLLMVNEATRFQTVSGFVA-GLLHPKAV--------------

EKKSGKI--------------GNEMKAQVNGWQDLS

>ADDN01000393

YGSFIDDLRRRFGKRG-----HFS-------HN--------RPVLP----PFDET---VP

-----PR----WWFHVVL-----RTT-Q---TT---T-L----TLAIRADNLYLEGF-RS

-------SDNT------WWELT-------------------------------------

----------QGFI----PG------------A-TYMGFGG-SYSD----L--LG-ETDA

----------MVRVEL-GPQQMTEAVNVLAGRRRAD-----------------------

--KGSEAKQKQAGKMLATLLLMVNEATRFVTVSAFVA-GLMHPKVA-------------

GTKSGVI--------------TALMKEQVNGWSDLS

>ABXC01000035

YGGFISGVRNQLVRHA-----RATR------HL-------ELVLLH----P--QE---ED

-----PK--KAPWFRVALRCS--SSG-----DS---A-V----LLRVRTDNLYISGY-QS

-------SDGR------WWEFR-------------------G-----------------

----------GSVI----DA------------A-TELAFTD-SYES----M--GR-AAG-

-------LE-LESVTL-SKKDLEAAVGQLAAAAGRRPS---------------------

AGGAGSSSQQDTARSLMVVAVMVCEAIRFRSVAGALA-HVMCN----------------

AARFGTL--------------PAHMVAQVKNWSSLS

>P84786

YTDFIKNLRRSLATW-------YL-------HG--------VPVLP----LYNQE---AD

-----P-----RGFDLKL-----TFR-----GQ---V-T----TVRIHRDDLVLRGY-QM

------QGAGK------WLELE-------------------R-PST-------------

---------QTGHLI----EG------------S-ELLEFGP-SYEE----L--AA-AAQ-

-------QD-ILDISY-NKNALQDAVSKLAV----------------------------

-----STNTRDRARSLIVVSQMFCEATRFVDIANHFA-FNLES----------------

SEPVKLP--------------QWMQNDLEKNWVRFS

>AF289116

YTAFIGKIRSRLEDTNVA---GVR-------PN--------IPVLP----VYNQI-----

----RPP----QGFDIVL-----TAG-----AH---T-T----TARFRRDNLYLVGY-EM

-------KTDT------WLEFG-------------------R-RRD-------------

----------PQLI----RG------------S-EFLGFDG-SYTD----L--ER-HAGS

----------VTKMDI-NRAILMTSVQDLTE----------------------------

-----STTTSVRAKALVVVIQMICEAARFISVENHFA-SNLAT----------------

-QRAKLP--------------LWMMEDLQKNWARIS

>ACYX02098007

YTDFIEGLRHRLRER-------SS-------HN--------RPVLP----P--QE---NP

-----PR----RWFDLKL-----LSSSS---NH---A-I----TLRIRRDNLYLDGY-QM

------ENPNQ------WLEFG------------------D-PSP---------------

----------QHLI----PG------------S-SFLGFDG-GYPD----L--QR-VAA-

-------ET-RKNISL-GTEELKKAVNKLAT-----------------------------

-----STSSKERAHHLIVVIQMMCESIRFDRISKHLA-TQFSG-----------------

---SSGP--------------PEWMLALENGWGDLS

>FR617512

YRQFIERLRPRLAVR-------YS-------HN--------LPVLP----L--QE---NP

-----PT----RWLDLIL-----RTR-----TS---A-I----TLRIRYGNLYLDGY-RM

GT----SSSDT------WWEFG-----SSTA---------N-PRP---------------

----------PQLI----SG------------S-SFLGFTG-EYTK----L--ET-AAG-

-------VV-TNSIRL-GQQALIDAVNTLAI-----------------------------

-----STDRTSKARSLIIVIRMICESIRYARISNHIT-NNFT------------------

---GFSP--------------ETWMSTLEKNWGRLS

>FR606563

YKKFIEDLRNNKKLGK-----TFS-------HD--------IPVLA----P--QE---KT

-----PT----RWFHVVL-----RTD-----EK---E-I----TLSIRCDNLYLECY-QM

------GKAGA------WMEFG------------------S-DTK---------------

--------------K--PPS------------P-SFLGFDG-DYGD----L--EK-AAGI

S----AHLS-RNSISL-GQQALKGAVNALANS----------------------------

-----STERQVRARSLIIVIRMICESIRFPRISNHIA-KNFKN-----------------

---GIEP--------------ESWMSDLENNWSSLS

>AFRG01000322

YTSFIAQLRTRAADPN-----NLS-------HS--------IPVLY----P--QV---NP

-----PA----RFFDVVL-----RTA-----QA---A-V----RFRLRRDNLYLEAY-RN

------ETAEQ------WFEFA------------------N-DGY---------------

----------LHLV----HG------------S-QFLSFTG-NYMG----L--TR-VAG-

-------ED-RDQIRL-GRGPLNSAVNDLAT-----------------------------

-----SEDNGARARALIIVIQMVCESMRFSWISEQIG-STYTT-----------------

--GGFVP--------------SGQMLDLENGWGLVS

>AFSE01000013

YRSFIAQIRTRAADPN-----NLS-------HS--------IPVLF----P--QV---NP

-----PT----RFFDVVL-----RTA-----QA---A-V----RLRLRRDNLYLEAY-RN

------EAGVQ------WFEFA-------------------N-GGD--------------

----------LHLV----TG------------S-EFLPFTG-NYMG----L--TR-VAG-

-------ED-RDQIRL-GQAPLNTAVNDLAN-----------------------------

-----SGDNVARARALIIVIQMVCESTRFSWISDQIG-STYTT-----------------

--GGLIP--------------SSQMLDFENGWGLVS

>AFRB01000334

YRSFIAQLRTRAADPN-----NLS-------HS--------LPVLY----P--QV---NP

-----PT----RFFDVVL-----RTA-----QA---A-V----RLRLRRDNLYLEAY-RN

------EAAEQ------WFEFA-------------------N-DGG--------------

----------LRLV----NG------------S-EFLAFTG-NYMG----L--TR-VAG-

-------ED-RDQIRL-GQGPLNSAVNDLAT-----------------------------

-----SQDNVARARALIIVIQMVCESMRFSWISDQIG-STYTT-----------------

--GGLIP--------------SSQMLDFENGWGLVS

>ADFL02000265

YRSFIAQLRTRAADPN-----NLS-------HS--------LPVLY----P--QV---NP

-----PS----RFFDVVL-----RTT-----QA---A-V----RLRLRRDNLYLEAY-RN

ET----ETAAQ------WYEFA-------------------N-GGD--------------

----------LHLV----TG------------S-ELLRFTG-SYTS----L--TR-VAG-

-------ER-RDQIRL-GQGPLSSAVNDLAT-----------------------------

-----SQDNVMRARALIVVVQMVCESMRFSWISDQIG-STYTT-----------------

--GGLIP--------------SSQMLDFENGWGLVS

>AFRE01000036

YKSFIAQLRTRAADPN-----NLS-------HS--------LPVLY----P--QV---NP

-----PT----RFFDVVL-----RTG-----QA---A-V----RFRLRRDNLYLEAY-RN

------EAGGQ------WFEFA-------------------N-GGN--------------

----------LHLV----TG------------S-QFLGFGG-NYMG----L--TR-VAG-

-------ED-RDQIRL-GQGQLNTAVNDLAT-----------------------------

-----SEDNSTRARALIIVIQMVCESMRFSWISDLIG-STYTT-----------------

--GGFLP--------------NPQMLDLENGWGLVS

>AFRF01000004

YRSFIAQIRTRAADPN-----NLS-------HS--------LPVLF----P--QV---NP

-----PT----RFFDVVL-----RTD-----QA---A-V----RFRLRRDNLYLEAY-RN

------ETGEQ------WHEFA-------------------N-EEE--------------

----------LHLV----TG------------S-ELLRFTG-SYTS----L--TR-VAG-

-------ER-RDQIRL-SQGQLISAVNDLAT------------------------------

-----SENDGTRAVALIIVVQMVCESARFSLISDQIS-STYTT-----------------

--GGLIP--------------SSQMLDLENGWGLIS

>EJP64846

YKNFIERIRRRVQDSG-----VSS-------HG--------RPVLP----P--QR---MP

-----PT----SWFDIVL-----RTQ-----SQ---A-V----RLRIRSDNLYLDGY-RA

------EDSDQ------WFEFG-------------------V-ANVD-------------

----------PQLI----DG------------S-THLGFDG-GYNS----L--QT-TAA-

-------RR-RSETAL-GQQQLTTAVNQLAT------------------------------

-----TADRRERARSLLVVIQMICEAIRFTHISDTIT-NNYSE-----------------

---GVTP--------------DERFTALENGWGDVS

>JPHH02000315

YRSFIERMRRRVQDPN-----VLS-------HG--------RPVLP----P--QQ---TP

-----PS----RWFDMVL-----RTG-----SQ---A-V----RLRIRSDNLYLDGY-RA

------ENSDQ------WFEFD-------------------N-DGSD-------------

----------LHLI----AG------------S-RHLGFDG-GYVS----L--QR-AAN-

-------RR-RDETYL-GQQQLTTAVNQLAI------------------------------

-----TADRGERARSLLVIIQMISESIRLTHISDTIS-NSYTT-----------------

---GFIP--------------DSQITALENGWGDVS

>JMQE01001167

YKEFIESLRRRLQNEQ-----EFS-------HN--------VPILP----P--QR---NP

-----PD----RWFHVVI-----TTS-----SR---S-V----RLRIRRDNIYIDGY-QE

VM---EEDEGP------WFEFS-------------------N-LGN--------------

----------RHLI----PG------------S-QFLGFGG-GYTA----L--QS-AAG-

-------RE-RHQVNL-GQQALITAVNTLVA------------------------------

-----TNSRSDRARSLIVISQMLSEAIRFVTILNQIS-NNYET-----------------

---GAQL--------------PGTMTALQNGWGTIS

>KIJ38555

YRNFIEDIRRRLQYGS-----VLS-------HD--------RPLLR----P--QE---RN

-----PE----HWFEVGL-----TTSTP---DR---T-V----WLRVRSDNLYVDAY-RA

------ENVEQ------WSEFN-------------------N-SAG--------------

----------RHLF----EG------------S-RLLEFGC-NYNS----L--ER-HGGR

----------RIDTSL-GQPQLHTTVNWLFD------------------------------

-----AETVARRAQALIVIIQMISEAIRFLAITNFVV-TNWRN-----------------

---GTTP--------------TNQIIALENGWRDIS

>HS077718.1

YRRFIDDLRNRLAGDR------VS-------HD--------RPVLR----P--QP---LP

N-ETTAN----ERFQVVL-----RTS-D---AR---E-I----RLLFRPRNLYLDAY-RM

------QNSDI------WLEFS-------------------N-PSSR-------------

----------EHLI----PG------------S-TFLGFDG-DYGSS-HGL--EV-TAG-

-------RG-RGETPL-GRDSLRDAVNVLAT-----------------------------

-----SANSRDRARSLLVVIQMISESIRFEHITNFLL-DIYFEGSSNV-----------E

PPRTNLP--------------DDRILQLENSWGRLS

>AARH01008880

YRDFIEKLRDRLGVR-------FS-------HN--------RPALA----V--QE---EP

-----PT----RFFDLVI-----RTN-----DH---S-V----RFRLRMDNLYLIGY-QM

-------ENGQ------WLEFN-------------------N-ETG--------------

----------VHLIR--EQG------------T-EFLGFNG-SYNM----L--SN-VAG-

-------LS-MEEVRV-GFYNLGYGINQLAT-----------------------------

-----STTWKIRARYLIGVIMMICESTRLIPISDYMA-TNFDNSHGTSSDD---YNNYDN

RNREGQI--------------QPWITTLVRAWDAFS

>AGQN01263163

YRNLIADLRRRLSER-------TT-------HG--------RPTLP----P--QA---EP

-----PT----RFFDLVL-----RTS-----DH---S-V----RFRFRIDNLYLIGY-QM

-------ETGE------WLEFQ-------------------N-DSN--------------

---------PLTHFI----PN-----------S-TFLNFNG-SYRS----L--AK-ASK-

-------RN-TRETRV-GFYNIGIATNRLAT-----------------------------

-----STVGEVRAACLVVVIIMISESIRLLPVQDYVSDQNFENRPRIPPVD---YIRGGN

ADREGVV--------------EEWLLSLIRNWGSLS

>JYCQ01000009

YTQFIQGLRDRVASGD------LC-------YY--------LPVLS----R--QP---ST

-----PN----SWFDVVL-----RAG-----GQ---A-L----TLRIRRDNLYLEGY-RR

--------GTT------WYEFR---------------------HGG--------------

----------PLLI----PG------------A-EQLRFDG-SYIR----L--ES-WAR-

-------QG-REAIPL-GQQALVNAVHALVDR----------------------------

-----NINDQARARQLMVVIQMISESMRFQTINRHLA-NNWEN-----------------

---SEPP--------------PSTLVNLENAWGGLS

>CACC01024922

YQTFIQSLRSILSYST--------------SHD--------INVLM----P--QT-----

----QPL----SWLDIRL-----TSG-----DS---T-I----ILRIDKRNLYVRGYSRD

-------DGAT------FWEFK-----D--------------------------------

----------TSLI----PG------------S-RTLTYTG-SYVKG-ITL--IK-VAD-

-------VN-RQTLPL-GLSNLRTAILDLARNEDP-------------------------

---TDKDALKDCARALLLLTQMIAESIRFQLITDHIV-TNWYN-----------------

---SAPL--------------TLQLVELQQGFGDFS

>CACC0102492

YQTFIQSLRSILSSST--------------SHN--------IDVLM----P--QT-----

----HSL----SWLDIRL-----TSG-----AS---T-I----ILRIDKRNLYVRGYSRD

-------EGAK------FWEFS-----D--------------------------------

----------SSMI----SG------------S-SPLAYAG-SYVDG-YTF--ID-AAGQ

D------VT-RETLQL-GLPNLSNAIANLARALDPN------------------------

--STQNNALQNCARALLVLTQMIAESTRFQLITDHIV-KNWYN-----------------

---SAPL--------------TSQLVELQQGFVSFS

>CACC01008026

YQTFIQNLRSILSSSI--------------SHD--------IHVLM----P--QT-----

----EPV----SWLDIRL-----TSG-----DS---T-I----ILRIDQRNLYVRGYSRD

------IEDEP------FWEFS-----D--------------------------------

----------SSLI----PG------------S-RHLAYGG-SYVDG-YNF--VV-AAG-

-------TT-RLNVHL-GLPNLRNAIAILATTEDPN------------------------

--STQNNALQDCARALLVLTQMIAESARFQLITNHIV-TNWFN-----------------

---SARL--------------TSQLVRMQQSFGTFS

>JPUM01000046

YADIIARLREEIRQRCPPPP-----------EG----LDNTAIVLP----Q--QQ---RL

TRGWGDE----HWFEVEL-----ETS-----QG---A-V----TFRLRNDNLYLDAY-RV

------RGSST------WWEFQ-------------------N-ERG--------------

----------VSLI----DG------------S-ESLGFGG-SYPN----L--EG--QG-

-------AT-REGTSL-GGPALVDAVNQLNRQTTLD------------------------

-----AAERRQRAKSLLVVVQMLSESLRLNAFTAWLA-RYWIE-----------------

---YRAP--------------EPQYLRLQNLWGTLS

>ADOT01000134

YLNLIVDLRRSAAAGD-----RVV-------HG--------LGVLP----A--ET---AN

-----PA----DFIDVGL-----KTT-----TN---L-I----TVRLRRDNLYLIGY-LP

------SYRTR------WYEFS-----D--------------------------------

----------THLI----QG------------S-EALPHDG-HYTT----L--EK-KAN-

-------AS-RRDIAL-GFGPTEGAVNALAQKGA--------------------------

-----PSSVEIHARSLIILIEAISEAMRSTVACEFIR-SKWEL-----------------

-GTAEKP--------------TLRIVTLENSWDPIS

>AEVU01000543

YKKLLEDLRGELEACR------RP-------VA--------LHVLP----P--QT---PN

-----PTAATVDWFDLGL-----HTG-----GF---T-I----TVRLQADNLYLLAY-RV

EG----FANPE------WRALA-----D-------------A------------------

----------GPLV----PG------------S-RALPGGF-TYSR----L--ET-QAD-

-------LR-RPELPL-GLGAFRGAASYLATHLQDP------------------------

---RNQDEKKALAKSLLVLVQMLSEALRFRVIGDHVA-----------------------

-AEGAQI--------------TERMARLENDWADIS

>EJP61635

YTKFLEDLRTELAACR------RS-------EA--------LRVLP----P--QT---LN

-----PTAETVDWFDLGL-----HTG-----GF---T-I----TVRLRADNLYLLGY-RV

EP----FANSN------WRAFS-----D-------------A------------------

----------QRLV----TG------------S-TALPGGF-TYSR----L--ET-QAG-

-------LR-RPELPL-GLSALRGAVSYLATHPQDP------------------------

---SNEEEKKSLAKALLVVVQMLCEAMRFRLIGDHVA-----------------------

-AESAQI--------------TERMTRLENDWADIS

>AQGS01000454

L--YLP---HP-------KN

-----PP----EFFDVVL-----ETT-----SS---N-L----DVKLQKDNLYLLAY-R-

-------SNDG------WREVK-------------------G------------------

----------SELIC-----------------S-TKAEFPA-DYPD----L--EK-VAAF

DTSK-GLKG-RYNTRV-GKSSLETAIDTLL------------------------------

---SNKASPKEAARAFLVLTQMLPESIRIREISEHIG-TSWGE-----------------

---NEII--------------PFTLVEKENGWKQAK

>DAA51819

NTTFIQEIRRELGDTS------VM-------QE--------AFQLP----P--QT---DT

-----P-----SFQDLVL-----IAG-----ES---T-A----TLRLRTDNLYVVGF-RN

-------QAGR------WFEFR-------------------H-ENG--------------

--------GLPPII----PG------------A-TMLHFSG-SYVG----T--NS-LAP-

-------LNRPRDVNI-GRHTIESAVHSLAQST---------------------------

-----GEEDQRLKDWLRTLIVSFIESIRFNSVASAVA-EALRN-----------------

-DQVNQL--------------EEHHIHQIRNWSAIS

>XP008665567

NTTFIQEIRRELGDTS------VM-------QE--------AFQLP----P--QT---DT

-----P-----SFQDLVL-----IAG-----ES---T-A----TLRLRTDNLYVVGF-RN

-------QAGR------WFEFR-------------------H-ENG--------------

--------GLPPII----PG------------A-TMLHFSG-SYVG----T--NS-LAP-

-------LNRPRDVNI-GRHTIESAVHSLAQST---------------------------

-----GEEDQRLKDWLRTLIVSFIESIRFNSVASAVA-EALRN-----------------

-DQVNQL--------------EEHHIHQIRNWSAIS

>JOWA01000098

---KGILP----P--QE---NP

-----PA----EYFDVVV-----HVK-----SKDDKPWT----RFRIRRDNLYLVED-KT

-------SEHR------WYIFR---NGD--------------------------------

----------RHLF----PD-----------QA-DTLNFDG-SFTS----L--MT-VARR

-------WQ-ISDVRL-GKQALRKAALTLSSSP---------------------------

---EINTDNKDIAQALILVVIMICEAMRFHLIRDHIA-ESYDS-----------------

---ACKM--------------TEQMVGCLKSWQDLS

>DBL01002728

YDSLIKTIRERLAVGE------SI-------EG--------VPILA----P--QV-PAGG

-----QL----EFFDVNLSYTD-RGT-----TA---T-V----QARFRTDNLYLVRY-RP

------ANSDV------WTEL---------------------------------------

------------------DG-------------------RE-NYNN----L--TT-DAG-

-------MG-LEDIPL-SSSRIGAAVTTLA------------------------------

---GGETDPGRRARAILTLIFAIAEAARFRDISTLIS-NSWWN-----------------

---ESTP--------------GVQFANRVRSWARLS

>AMZD01000108

YDTLIRTIRENLAVGE------SI-------EG--------VPILA----P--QV-PAGG

-----EP----EFFDVNLSYTE-FRT-----TS---T-I----QARFRTDNLYLVRY-RP

------GNVDA------WQEL---------------------------------------

------------------DG-------------------RE-NYNS----L--TT-NAG-

-------MG-LDEIPL-SSSHIGVAVTTLA------------------------------

---GGEADPGPRARAILTLIFTIAEAARFRDISSLIS-RSWWN-----------------

---ASTP--------------GVHFASRVRSWARLS

>AOSW01007610

YDAFIVELRNLLATGD------WV-------ES--------VPVLA----E--QV-PPGG

-----AL----EFFDINLTYND-RGE-----QH---I-V----QLRFRTDNLYFVGY-RP

------QNSNI------WFELA-------------------H-EDG--------------

--------GTNTLIQDQEAG----------RAT-QLLPLGE-NYCT----L--TE-VAG-

-------MR-LEDIPL-SSSRIGAAITTLATDN---------------------------

---GAPANEGNRARAILTLVFAIAEAARFRDISRLIS-NAWWT-----------------

---ESAP--------------GTQYANRVRSWCRLS

>JRVE01000053

YDAFITDLRNRLGVGG------TV-------QG--------VPLLT----P--QV-PAGG

-----QH----EFFDVNLTYTD-PGI-----TS---T-L----QARFRTDNLYLVRY-RQ

------NESSA------WQEL---------------------------------------

------------------DG-------------------NE-NYNS----L--IR-DAG-

-------RR-LEQIDL-SSTHIGAAITTLG------------------------------

---NNVTDAGQRARALLTLILSIAEAARFRNVSSVVS-NNWYV-----------------

---GSRI--------------SPEYASQIRSWARLS

>AORF01001931

YIQNIQQLRRELAVGD------YI-------ES--------VGTLA----P--QV-PPGG

-----TL----EFFDIVLSYTDDHGT-----TH---T-L----TVRLRTDNLYLVAW-RR

------PNTTV------WYELG-------------------H-ESG--------------

--------GAATIV---DPG----------ATT-QLLSLRE-SYVA----L--ER-VAG-

-------WR-LQDIPL-SARRIGEAIRTLATTE---------------------------

--VNSSTDLTPIARSVLTLSFTLAEATRLRSISALIE-TAWWN-----------------

---ESSP--------------GEHYTSQVRSWGRLS

>AAX95110

YSKFIEDLRKKLANHPHK---EMI-------HD--------VPVLA----R--QQ-SPRQ

-----PA----RWMYINL-----VGR-K---KD---R-A----TVAVRDDNVYLMGF-RN

-------MNGE------WFHLG-------------------F-------------------

------SKWSVPIL----PESS------------KFLECDV-AYRN----L--LDVPQGE

-----QVMSRLVEVEL-RKTVVLDAVHRLSRYTQRG------------------------

---QDDRIDRFTKRDLARLIIVICESARMRPHYTTVN-KGFVH-----------------

-DETTSL--------------TKLHVFYLWNWGLMS

>EEE51701

YSDFIIDLQSRLAEHPEQ---ANF-------HG--------RPVLA----R--QC-HPKQ

-----PA----RWLYINL-----VGE-N---ND---R-A----TLAVRDDNVYLIGF-RN

-------LNGK------WFHLG-------------------F------------------

------SLRSVPIL----PEPS------------TFLECDV-TYRS----L-----LGG-

-----NVKDVLVRVDV-RKISVIHAVHRLSGYAQRR------------------------

-----CGVDGATKRDLARLIVVICESARMAAHYNTVN-DGWLI-----------------

RNRQIRL--------------DLWHVDYLWNWGLMS

>Q00531

YDEFIEKVRKALAGTA-----GAK-------VGPKPKSKVESPVLDKGTFPV------EQ

-----PP----RWIHVEL-----HGK-----TQ--GT-TTPKPKVAIRSDDAYIMGF-TN

-------STGR------WFQLS-------------------K------------------

------TGTTYKLV---------------DDKA-VMAGFDG-NYNT----L-----VGG-

-------VNNLPTLNL-NKFSMAQAAAALWNKASTLSGGIG------------SDVVDD-

DDGDMLRANDPVKQAVATLAVAVCEAARFSPVSKVVN-AGWIK-----------------

--DKVSV--------------TPDEVNYIKEWGDLS

>L12243

YVNFLNELRVKLKPEG------NS-------HG--------IPLLR----K--KC--DDP

-----GK----CFVLVAL-----SND-N---GQ---L-A----EIAIDVTSVYVVGY-QV

--------RNR------SYFFK-----D-------------A-PDA--------------

--------AYEGLF----KN----------TIK-TRLHFGG-SYPS----L--EG-E-K-

-------AY-RETTDL-GIEPLRIGIKKLDENAID-------------------------

-----NYKPTEIASSLLVVIQMVSEAARFTFIENQIR-NNFQ------------------

--QRIRP--------------ANNTISLENKWGKLS

>M98344

YKQFIEALRERLRGG-------LI-------HD--------IPVLP----D--PT-TLQE

-----RN----RYITVEL-----SNS-D---TE---S-I----EVGIDVTNAYVVAY-RA

--------GTQ------SYFLR-----D-------------A-PSS--------------

--------ASDYLF----TG----------TDQ-HSLPFYG-TYGD----L--ER-WAH-

-------QS-RQQIPL-GLQALTHGISFFRSGG---------------------------

------NDNEEKARTLIVIIQMVAEAARFRYISNRVR-VSIQTG----------------

--TAFQP--------------DAAMISLENNWDNLS

>EU008736

YKQFIEALRASLTDR-------LI-------HG--------IPVLR----D--PT-TVEE

-----RN----RYITVEL-----SNS-E---RE---S-I----QVGIDVTNAYVVAY-RA

```
--------GTQ------SYFLR-----D-------------A-PRY--------------
--------ASTYLF----TD----------TQQ-HSLRFDG-SYSD----L--QR-WAQ-
-------QS-REQISL-GLQALTHAISFLHSGT---------------------------
------NDDRERAHTMIVIIQMASEAARYRYISNRVG-VSIRTS----------------
--TAFQP--------------DPAMLSLENNWDNLS
>AY039801
YTQFIEALRAQLASGE------EP-------HG--------IPVMR----E--RS-TVPD
-----SK----RFILVEL-----SNWAA---DS---P-V----TLAVDVTNAYVVAY-RT
--------GSQ------SFFLR----ED-------------N-PDP--------------
--------AIENLL----PD----------TKR-YTFPFSG-SYTD----L--EG-VAG-
-------ER-REEILL-GMDPLENAISALWISN---------------------------
-----LNQQRALARSLIVVIQMVAEAVRFRFIEYRVR-GSISRA----------------
--EMFRP--------------DPAMLSLENKWSALS
>EL690296
YTQFMKDLRDQLASGD------ER-------YG--------IPVLR----D--PS-TVPD
-----SQ----RFLLVEL-----SNW-G---EA---S-V----TLAVDVINAYVVAY-QA
--------GDQ------SYFFR-----D-------------A-PDV--------------
--------AFSNLF----TN----------TER-QTLSFNS-SYPA----L--QR-VAG-
-------ED-RENIDL-GILALEEAISSLHRTS---------------------------
-----SVQQNTQARSLIVSIQMVSEAARFRYIEQRVR-QSITSG----------------
GYQTFRP--------------DASMLSLENNWGALS
>ADF45510
YTQFMEALRDQLGSGY------EA-------HT--------IPVLR----D--VS-TVDT
-----SQ----RFLLVEL-----SNF-G---EA---T-I----TLAVDVQNAYVVAY-QA
--------GDQ------SYFFR-----D-------------A-PEV--------------
--------AFSNLF----ND----------TQQ-STFNFTG-SYVD----L--EG-RSR-
-------QD-REDIDL-GILALELAISSLRRSS---------------------------
-----AAAESTIARSLIVCIQMVSEAARFRYIERRVR-QSITSA----------------
GYEHFRP--------------DAGMLSLENNWGALS
>JPQF01109211
YSQFVQALRKELISGA------ES-------YS--------IPLLR----Q--ES-KVSN
-----AQ----RFVLVKL-----SNA-----KA---S-T----TLAIDVVNVYLVAY-QV
--------EAS------SYFFN-----D-------------T-SAA--------------
--------AFSDLF----KG----------TTK-TRFKFSG-GYPD----L--KN--LG-
```

-------AD-RENVDL-GAISLDNAIFSLNKYS---------------------------

------SDPNKIAAPLLVVIQMVSEASRISHIERKIM-TNFY------------------

--QRFRP--------------LGDVISLENKWSALS

>AF508915

YFRFITLLRDYVSSGS------FS-------NE--------IPLLR----Q--STIPVSD

-----TQ----RFVLVEL-----SNE-G---GD---S-I----TAAIDVTNLYVVAY-QA

--------GNQ------SYFLR-----D-------------A-PRG--------------

--------AETHLF----TG----------TTR-SPLPFNG-SYPD----L--ER-YAG-

-------H--RDQIPL-GIDQLIQSVTALRFPGG--------------------------

-------STRTQARSIIILVQMISEAARFNPILWRLR-QYINSG----------------

--ASFLP--------------DMYMLELETSWGQQS

>AF249280

YRDFLSNLRKTVATGT-----YEV-------NG--------LPVLR----R--ES-EVQV

-----KS----RFVLVPL-----TNY-N---GN---T-V----TLAVDVTNLYVVAF-SG

--------NAN------SYFFK-----D-------------A-TEV--------------

--------QKSNLF----VG----------TKQ-NTLSFTG-NYDN----L--ET-AAN-

-------TR-RESIEL-GPSPLDGAITSLYHG----------------------------

---------DSVARSLLVVIQMVSEAARFRYIEQEVR-RSLQQA----------------

--TSFTP--------------NASMLSMENNWSSMS

>AF213983

YRDFIEKLREIVTRGA-----TTI-----AGTS--------IPVLN----R-----VVPD

-----SR----RFVYVRL-----INL-D---GN---V-V----TIAVDVTSLYVVAF-SA

--------NNN------GYFFS-----D-------------S-TET--------------

--------ERTNLF----VG----------IPRGDPLGFTG-NYNS----L--EN-WAG-

-------AD-RGSIPL-GPALLNKAIRNLRSNG---------------------------

-------RDSKAAKSLIVVIQMVSEAARFRRIEEQVR-RSIADQ----------------

--DTFTP--------------GSLMITMEKKWSKMS

>X62372

YSKFITALRKALPSKE------KV-------SN--------IPLLL----P-----SASG

-----AS----RYILMQL-----SNY-D---AK---A-I----TMAIDVTNVYIMGY-LV

--------NST------SYFFN-----E-------------S-DAK--------------

-------LASQYVF----KG----------STI-VTLPYSG-NYER----L--QN-AAG-

-------KV-REKIPL-GFRAFDSAITSLFHY----------------------------

-------DSTAAAGAFLVIIQTTAEASRFKYIEGQII-ERIP------------------

--KNEVP--------------SPAALSLENEWSALS

>X57682

YGMFIKDLRNALPFRE------KV-------YN--------IPLLL----P-----SVSG

-----AG----RYLLMHL-----FNY-D---GK---T-I----TVAVDVTNVYIMGY-LA

--------DTT------SYFFN-----E-------------P-AAE-------------

-------LASQYVF----RD----------ARRKITLPYSG-NYER----L--QI-AAG-

-------KP-REKIPI-GLPALDSAISTLLHY----------------------------

-------DSTAAAGALLVLIQTTAEAARFKYIEQQIQ-ERAY------------------

--RDEVP--------------SLATISLENSWSGLS

>AY669811

YGVFISNLRKALPNER------RL-------YD--------IPLLR----S-----TLQG

-----SQ----RYALIHL-----TNY-A---DE---T-I----SVAIDVTNVYIMGY-RA

--------GDT------SYFFN-----E-------------A-SAT-------------

-------EAAKYVF----KD----------AMRKVTLPYSG-NYER----L--QT-AAG-

-------KI-RENIPL-GLPALDSAITTLFYY----------------------------

-------NANSAASALMVLIQSTSEAARYKFIEQQIG-KRAD------------------

--KTFLP--------------SLAIISLENSWSALS

>TKU25675

YGVFISNLRKALGNER------RL-------YD--------IPLLR----S-----TLPG

-----CE----RYALIHL-----RNY-A---DE---T-I----SVAIDVRNVYIMGY-RA

--------GDT------FYFFN-----E-------------A-SAT-------------

-------EAAKYVF----KD----------AMRKVTLPYSG-NYER----L--QT-AAG-

-------KI-RENIPL-GLPALDSAITTLFYY----------------------------

-------NANSAASALMVLIQSTSEAARYKFIEQQIG-KRVD------------------

--KTFIP--------------SFAIISLENSWSALS

>EU309692

YKTFIKNLREALPKDG------KV-------YD--------IPVLL----S-----TVMD

-----SR----RFILIDL-----VNY-D---GQ---S-I----TAAIDVLNVYIVAY-ST

--------GTV------SYFFQ-----Q-------------V-PAQ-------------

--------APKLLF----KG----------TQQ-RTLPYTG-NYEN----L--QT-AAK-

-------KL-RENIEL-GLPALDSAITTLFHY----------------------------

-------NAEAAASALLVLIQTTSEAARFRYIELQIA-NNVG------------------

--TKFKP--------------SQTIISLENNWSALS

>AGCB01010835

YTSFIGQLRNALPTTG------KV-------CN--------IPVLP----S-----TASG
-----LQ----WFTFFSL-----TNY-N---DE---T-I----TVAVNVTNVYIVAY-RA
--------GAV------SYFFE-----D-------------T-SAE-------------
--------AWNLLF----TD----------TRK-VKLPYSG-NYDR----L--QN-VVG-
-------KQ-RDFIEL-GFPALSSAITNMFYY---------------------------
-------DYKPTAAALLVLIQSTAEAARFKYIEQQIS-QYIN------------------
--HNFLP--------------DLAIISLENNWGALS
>AF256085
YSAFIQSLRTHLSSGT------SE-------YG--------IPLMR----A--QN--PSS
-----SQ----ELLLVEI-----FGW-D---NE---P-V----TLVLNLVNAYVIAY-QA
--------QGH------YYLLH-----D-------------T-PDN-------------
--------PQ---L----YG----------SDA-HRLSFDG-SYPA----L--QH-VAG-
-------EY-RENIDL-GINELGSAILVLHQWSP-------------------------
-----PTVERTVARSFIVLIQMVSEAARFRAIETRVR-RNIIQV---------------G
DYRSFRP--------------GAGMLDLETNWGTLS
>AF256084
YSAFIQSLRTHLSSGT------SE-------YG--------IPLMR----A--QN--PSS
-----SQ----QLLLVEI-----FGW-D---NE---P-I----TLVVNLVNAYVIAY-QA
--------QNR------YYLLH-----D-------------T-PDN-------------
--------PQ---L----YG----------SNP-HRLSFDG-SYPA----L--QR-VAA-
-------EN-RENIEL-GINELGSAILVLHQWSP-------------------------
-----PTVERSVARSLIVLIQMVSEVARFRAIETRVR-RNIIQV---------------G
DYRSFRP--------------GAGMLDLETNWEPLS
>U78041
YSDFLQNLRNRLSSGT------SV-------HD--------IPLLP----A--QS---GS
-----QQ----DLLFVRL-----FDW-G---NR---P-I----TLVLNRVNAYVVAY-QA
--------QNR------FYLLA-----D-------------T-PAN-------------
--------PQ---V----YG----------NNP-HRLTFIG-SYGA----L--QN-VAK-
-------QN-RENIDL-GINPLATAISTLHNWSP-------------------------
-----PTVETSVARSLIVLIQLVSETARFRAIEQRVT-NNIIDQVT------------PI
RYDNFRP--------------SVGIIDLQTNWQTLS
>ACYM01011001
YNTFIKALRAQLTNGA-----TAI-------YD--------IPVLN----P-----SVPD
-----SQ----RFLLVDL-----SNN-G---NN---T-I----TVAIDVVNXSVVAY-RA

-------RAAR------PYFLA-----D-------------A-PDE--------------

--------ALDXLF----ND----------TRG-FFLPFTS-NYXD----L--EK-AAE-

-------KS-RDKIPL-GLTPLHNAITSLWNH----------------------------

-------ESEEAAVSLLVIIQTVFEAARFRVIEQRVR-NSISSK----------------

--ANFIP--------------DPAMLSLENNWLAIS

>DQ672577

YNTFIAKLRQELSIGT-----QKV-------AN--------ITVLK----H--H--VSSN

-----TQ----RFLSINL-----FNY-N---GD---K-I----TLGIDVFDVYVVGF-LT

--------GTN------SYIFK-----E-------------A-PELA-------------

-------YNKSLLF----PG----------SVR-ENLSYTG-GYDD----L--KR-R-G-

-------AG-REDIPL-GLLPLDTAITNLFHR----------------------------

-------DSTSFRRSFIVIIQMVSEAARFKIIEAKIA-KNLYGE----------------

--NTFKP--------------DQAIISLENNWGALS

>DQ683359

FNNFVDAVRDLVVSGP------PV-------HG--------LLRLR----D--RS-TVPI

-----SQ----RFILVEL-----YNY-E---AT---P-I----TLAIDVTNLYVVGY-RT

--------GDL------FYYFL-----D-------------S-AV---------------

--------DTPGIF----RE----------LPH-IELPFNS-SYPA----L--QH-EAG-

--------D-RVNIPL-GITELDQSIQDLRHY----------------------------

-------RRTNLARPFIVVIQMVSEAVRFRYIENLVV-LSIRAD---------------G

SRQNFRP--------------TRTMISLENNWDTLS

>X52908

YTNFIRAVRGRLTTGA------DV------RHE--------IPVLP----N--RV-GLPI

-----NQ----RFILVEL-----SNH-A---EL---S-V----TLALDVTNAYVVGY-RA

--------GNS------AYFFH----PD-------------N-QEDA-------------

-------EAITHLF----TD---------VQNR-YTFAFGG-NYDR----L--EQ-LAG-

-------NL-RENIEL-GNGPLEEAISALYYYSTG-------------------------

-----GTQLPTLARSFIICIQMISEAARFQYIEGEMR-TRIRYN----------------

--RRSAP--------------DPSVITLENSWGRLS

>AY069946

YAQFIKDLREAFGFSY------SS-------HE--------IPVLR----A-----TVAP

-----NQ----KFIVAKV-----INV-A---NL---E-V----SLGLNVVNAYLVGY-KV

--------GGT------SYFFN-----D-------------P-ESLA-------------

-------DAKTYLF----TD----------TKQ-QTLSFTG-SYAD----F--LS-RAN-

-------VH-REDVDL-GVQALDNYIYTLEKSS---------------------------

-------KPADIAKPLVGFIEMVPEAARFKYIEKKVL-SQIS------------------

--KTLRP--------------GGDIISLENNWGDLS

>BABX01103831

YAKFITDLRETFGSSG------LS-------HG--------IPVLR----A-----TVAA

-----NQ----KFFVAKV-----INA-G---DI---E-V----SVGLNVINAYLVAY-KV

--------GSN------SYFFN-----D-------------S-ESLA-------------

-------DAKKYLF----TD----------TKQ-QTLAFTG-SYAD----F--ES-RAK-

-------VH-REEVDL-GVVALDNYIYDLQKSS---------------------------

-------LPADIAKPLVSFIQMVSEAARFKYIENKVL-DQIS------------------

--QTFRP--------------RGDILSRENSWDDLS

>ACHR01003818

YQSYIQTIRQKFGSKT-----HKL-------YG--------IPVLH----H-----SLSN

-----SD----RFHLIDA-----GNE-P---GD---T-I----TFAVDAQDMSVVAY-LA

-------GDND------SYFFS-----N-------------A-PKF--------------

--------AFDILF----PK----------TNQ-NLLNFDN-SFKS----I--EI-AAN-

-------TT-REATPL-GLKPSNAAIANLFHY----------------------------

-------DPVLAPVSFLIVFQMVFESAKFKFIEQRIV-NSITNG----------------

--EAFTP--------------DLAMLSLEDNWSELS

>P85101

YSSFLDSLREEFGRGT-----PKV-------CN--------IPVTK----K-------AN

-----ND----KFVLVNL-----VLPFN---RN---T-I----TLAFRASDAYLVGF-QD

RD--SKTNKLR------ANFFS-----D-------------E-YRAL-------------

------SGKYKSIF----TDAE--------VLA-PALPCAS-TYTD----L--QN-KAG-

-------VS-REKLSL-GVSSLQTAFTAVYGKV---------------------------

------FTGKNVAKFALISIQMVAEAARFKYIEDQVI-NRGMY-----------------

--SSFEA--------------GARITLLENNWSKIS

>D10227

YLSFITNIRTKVVDKT------EQ-------CT--------IQKIS----K-------TF

-----TQ----RYSYIDL-----IVS-S---TQ---K-I----TLAIDMADLYVLGY-SD

IA----NNKGR------AFFFK-----D-------------V-TEA--------------

--------VANNFF----PGAT-------GTNR-IKLTFTG-SYGD----L--EK-NGGL

----------RKDNPL-GIFRLENSIVNIYGKA---------------------------

------GDCKKQAKFFLLAIQMVSEAARFKYISDKIP-SEKY------------------

```
--EEVTV--------------GEYMTALENNWAKLS

>AY148091

YPPFITNMRNVLSEKDKNG-K-DV-----LLCT--------MKKIS----------TTVP

-----SP----RYAYVDI-----KAS-A---TQ---T-V----TLAIDRTNTYVLGY-RD

IF----GGTDR------AAFFK-----D-------------V-YD---------------

--------DAKDLF----PDAK-------GKNR-IKLSYGS-QYTT----L---------

-------GD-RTKVPL-GIKSLRISITAIYGEAAG-------------------------

-----TDLDKNRREFFLLALQMVAEATRFKYISDKIP-TERDY-----------------

--DTLKV--------------DNHMIALENGWDLLS

>AF445416

YPTFIQDLRNELAKGT------PV-------CQ--------LPVTL----Q-----TIAD

-----DK----RFVLVDI-----TTT-S---KK---T-V----KVAIDVTDVYVVGY-QD

KW----DGKDR------AVFLD-----K-------------V-PTV--------------

--------ATSKLF----PG---------VTNR-VTLTFDG-SYQK----L--VN-AAK-

-------VD-RKDLEL-GVYKLEFSIEAIHGKT---------------------------

------INGQEIAKFFLIVIQMVSEAARFKYIETEVV-DRGLY-----------------

--GSFKP--------------NFKVLNLENNWGDIS

>ABI64066

YSTVIQELRNALANGA------PV-------CN--------FPVTA----K-----TIAN

-----DK----RFVLVDL-----TTT-S---MK---T-I----TLAIDVTDVYVVGY-RD

LY----NNKDR------AVFLA-----E-------------V-PSV--------------

--------AIHDLF----PG---------VTNR-EMLTFSG-HYQK----L--QE-AAK-

-------VN-RENLEL-GVNKLGFAIESIYGKA---------------------------

------LNGKDIARFFLIAIQMMSEAARFKYIENEVS-NGLY------------------

--GSFTP--------------NPKVLNLENNWGDIS

>AB070925

YSSFMTRLRNAVEAPT------RA-------CG--------LQSTR----S-----TPLP

-----GA----EYIHVDL-----KIS-N---TQ---W-V----TLGIDVKDFYVWGY-QD

NV--RYNGNYR------ASFLN-----D-------------A-PQA--------------

--------AKNNLF----RG----------STI-RTTRFGG-NYNS----L--EP-AAG-

-------IT-RRNLVL-GIQNLDGAIRTVYGKQESE------------------------

-----LNKGNAEARFFLIAIQMVAEAARFKFMEEAIV-RNDN------------------

-----TPD-----------F-KRKMVAFQNDWDPIS

>AAB67746
```

YANFLRRLRSAVSGPT------RA-------CN--------LNITQ----S-----NPPI

-----DR----EYVYIRL-----QFS-D---TQ---W-V----VLGMAAKDMYIWGY-VD

NR--PGFGPGQ---PPESNFLM-----D-------------S-PPE--------------

--------ARQRLF----PG----------SNR-RITDYGG-NYNS----L--QQ-RAQ-

-------RN-RDNVPL-GLTSLDGALKSVYGKSTSQ------------------------

-----LNEGNAEARFFLTAIQMVAEAARFKYMERGIS----------------------A

PPANF----------------RQNMIAFQNGWARIS

>AGQN01148610

YQAFMQSLRRELSSGI------TS-------YD--------IPLLR----K--KA-EAIK

-----DK----QFVYVRL-----ENE-----KF---K-I----TFAVSTLNCYMIAY-QV

------DTKKR------CYFFK-----E-------------A-PPE--------------

--------AKTLLF----KE---------STPR-VNVNLST-NYNN----L---------

-------GN-RENVNL-GFKSLDDSLEAFTEFDS--------------------------

-----VNPTNKLRQILIVVIQMVAEAARSKYIQQKIEWKGFK------------------

--SGFFP--------------KGDILSYENKWEDLS

>AGQN01137446

YQAFMQSLRRELSSGI------TS-------YD--------IPLLR----K--KS-EAIK

-----DK----QFVYVRL-----ENQ-----SF---K-I----TFAVSTLNCYMIAY-QV

------DAEKR------CYFFK-----E-------------A-PPE--------------

--------AKTLLF----KE---------STRR-VNVNLST-NYNN----L---------

-------GN-RENVNL-GFKSLDNSLEAFKRFDS--------------------------

-----ENPTNELRRILIVVSQMVAEAARSKYIQQKIEWKGFD------------------

--SGFFP--------------RGDILSYENKWEDLS

>AGQN01137447

YQTFMQSLRRELSSGT------TS-------YD--------IPLLR----K--KS-EAIR

-----DK----QFVYVRL-----ENQ-----SF---K-I----TFAISALNSYVIGY-QV

------DVEKR------CYFFK-----E-------------A-SSE--------------

--------AKALLF----KE---------STRR-VNVNLNT-NYDN----L---------

-------GN-RENVNL-GFKSLDNSLEAFKRFDS--------------------------

-----ENPTNELRQILIVVIQMVAEAGRSKYIQQKMEWKGFE------------------

--SGFFP--------------KGDILSYENKWEDLS

>AGQN01213101

YQIFMQSLRRELSSNI------ES-------HG--------IPLLR----T--KS-AAVK

-----DK----QFVYVRL-----NNP-----SV---A-I----TFAISALNSYVVAY-QV

------DAERR------CYFFK-----E-------------A-PPN--------------

--------SKTLLF----KQ---------CTKK-VEVNLKT-NYDI----L---------

-------GN-REKTNL-GFKSLDNSLQAFKRFDS--------------------------

-----TNPTDELRQNLLVVIQMIAEAARSKYIQQKLEWKGFE------------------

--TGFFP--------------RADIISYENKWEDLS

>AJ537497

YTRFIRVLRDELAGGV------SP-------QG--------IRRLR----N--PA-EIQP

-----SQ----GFILIQL-----TGY-----VG---S-V----TLIMDVRNAYLLGY-LS

--------HNV------LYHFN-----D-------------V-SAS--------------

--------SIASVF----PD----------AQR-RQLPFGG-GYPS----M--RN-YAP-

--------E-RDQIDH-GIVELAYAVDRLYYSQ---------------------------

-------NNNQIALGLVICAGMVAEASRFRYIEGLVR-QSIVGP---------------G

DYRTFRP--------------DALMYSIVTQWQTLS

>ALYE01008201

YQMFMKDLQNALTVRA------DR-------SG-------DIPVLP----P--RSAEPSD

-----PQ----QYLLVKL-----SNG-----YQ---T-V----TLALDVSDVYILGY-QP

------GGSGN------SSFFS-----D-------------V-PTN--------------

--------VRNILF----PN----------TQD-LSLPFTG-NYGA----L--ET-AAG-

-------AY-RREIPL-GIGELRQHIDNMNYIQP--------------------------

-----STDRRPIARALIVCIQMISEAVRLRNIQQELL-TIAEPRHD-------------G

TYSVFYP--------------DGLVMEYETSWEDIS

>AY547315

YATFLNDLRNEAKDPS------LK------CYG--------IPMLP----N--TN----T

-----NP----KYVLVEL-----QGS-N---KK---T-I----TLMLRRNNLYVMGY-SD

PF---ETNKCR------YHIFN-----D-------------I-SGTER------------

------QDVETTLC----PNAN-------SRVS-KNINFDS-RYPT----L--ES-KAGV

-------KS-RSQVQL-GIQILDSNIGKISGVMS--------------------------

------FTEKTEAEFLLVAIQMVSEAARFKYIENQVK-TNFN------------------

--RAFNP--------------NPKVLNLQETWGKIS

>EU839992

YTTFITSLRNAARDAS------LR------CYG--------IPMLP----P--TT----Q

-----QP----KYVLVKL-----EAD-----AK---T-V----TLIYKRNNLYIMGY-SD

PF----NGRCR------YHIFS-----D-------------F-TGTER------------

------TEAINTLC----PDSN-------NREQ-KDIRYES-NYPS----I--EN-KAG-

-------KS-RTQVEL-GINILKSSIGKISGVST-------------------------

------FTESVEAGFFLVAIQMTAEAARYKYIENLVK-TNFN-----------------

--RNFLP--------------DHDMIRLEETWGKIS

>AAS67266

YGTFLSNLRNIVKDSK------LV------YEG--------IPMLP----A--PI----K

-----PA----KYLLAEL-----KAK-KAGTDI---T-I----TLAVSKNDLYVVAF-TD

QV----AGKLR------AHYFP-----D-------------I-SLA-------------

--------TAKAIF----PT---------ATQY-IQIGYTS-NYVS----I--EG-AAG-

-------SN-RVNFQL-GFVKLKEYMLNVYGKNVQD-----------------------

-----SDYRRSEARFLLAAIQMVAEAARFKYVESKAI-NNVV-----------------

------P--------------DYKVPSLENNWSKIS

>AAK28323

YNTFMQSIRNQAKDPS------LV------YEG--------IPMIR----P--PT----N

-----PD----TYILVEL-----ESR-KDNNEI---S-I----TLALSKNDLYVVAY-AD

KF----GGKVR------GHYFK-----N-------------L-GIST------------

------IDKAKKVF----PN---------VQDF-INITYGE-SYNQ----I--ET-NAG-

-------TN-RLSFPL-GFDNLKTFTNKVYGMDTKA-----------------------

-----GNYTQTEARFLLIAIQMVAEAARFKYIQGRAI-VTTA-----------------

------P-------------NNYKIVSLETNWGALS

>ABJ90432

YNTFLQAIRNQVKDPK------LV------YEG--------IPMIR----A--PT----D

-----PD----TYLLVGL-----ESV-KNNNKI---S-L----TLALSRNDLYVVAF-AD

KY----GGKVR------GHFFP-----N-------------L-KIDT------------

------IDKAKKVF----PD---------VEVL-VQIKYGE-SYSQ----I--ES-NAG-

-------TN-RLSSPL-GFDNLRTYMEKVYGLDTSA-----------------------

-----KDYSKTEARFLLIAIQMVAEAARFKYIQGRAV-VTTA-----------------

------P-------------ENYKIVSLETNWGSIS

>AAB96824

YSDFMTKLRTEAKGKD------PM------YVG--------LSMMP----K--PT----K

-----PP----TYLLVEL-----KVS-K---DM---S-I----TLSLSRNDLYVVGY-SD

MY----KGKCR------YHVFP-----D-------------H-DSKKP-----------

------PYEQHSLC----DKAK-------DAIR-KPIGYVS-SYEI-------ER-KAKV

-------RN-RKEIGL-GVNKLKTLIPKVYGSES-------------------------

------KDYQDEAKFLLVAIQTIAEAARFPYIEKIAE----------------------

--TAANP--------------DDTAICLENNWSKIS

>AM748700

YSSFVDKIRNNVKDPN------LK-------YGGTD-----IAVIG----P--PS-----

-----KE----KFLRINF-----QSS-----RG---T-V----SLGLKRDNLYVVAY-LA

MD---NTNVNR------AYYFR----SE-------------I-TSA-------------

--------ELTALF----PEAT-------TANQ-KALEYTE-DYQS----I--EK-NAQI

TQ---GDKS-RKELGL-GIDLLLTSMEAVNKK----------------------------

-----ARVVKNEARFLLIAIQMTAEVARFRYIQNLVT-KNFP------------------

--NKFDS--------------DNKVIQFEVSWRKIS

>X59260

YSSFLDQIRNNVRDTS------LI-------YGGTD-----VAVIG----A--PS-----

----TTD----KFLRLNF-----QGP-----RG---T-V----SLGLRRENLYVVAY-LA

MD---NANVNR------AYYFK----NQ-------------I-TSA-------------

--------ELTALF----PEVV-------VANQ-KQLEYGE-DYQA----I--EK-NAKI

TT---GDQS-RKELGL-GINLLITMIDGVNKK----------------------------

-----VRVVKDEARFLLIAIQMTAEAARFRYIQNLVT-KNFP------------------

--NKFDS--------------ENKVIQFQVSWSKIS

>AF219236

YSSFLDQIRNNMRDPK------LK-------YCSTE-----IAVIK----A--PS-----

----VAD----KFLRINF-----QGP-----RG---T-V----SLGLARVNLYVVAY-FA

VD---NRNVNR------AYYFR----TE-------------I-NSA-------------

--------ELRTVF----PEVT-------VANQ-RPLEYTE-DYQS----I--EK-NAKI

TT---GDKS-RKELGL-GIDLLISTIDKVNKK----------------------------

-----VRVVKDEARFLPNWIQMSAEAVRFRYIQNLVT-RNFP------------------

--KKFNS--------------DNTVIQYQTSWGKIS

>ADB83313

YTAFLTSIRDNVKGAG------LA-------YGGTN-----IPVIG----P--PT-----

-------D---TFLRIDF-----TLA-----TG---T-V----SLCLKRSDLYVVGF-VV

KN---DKGKFR------AYYFN----GQ-------------I-RSP-------------

--------QLDTIF----PEAK-----GTANQQ-IITEYAE-NYAS----I--ES-AAK-

-------VS-RKAAGL-GIGKLVTYLGAVNGK----------------------------

-----ARKVQDEAKFMLVAIQMVSEAARFGYIQNLVL-QNFP------------------

--NGFTP--------------DDKVLILERNWNRIS

>CAJ38823

```
YNQFVRDLRDRLARHN-----DRR-------HG--------VPVLR----Q--PG-XVLE
-----NE----RFILVEL-----TNMAE---DX---T-M----TWAIDVTNLYLVGY-QV
---------GR------SYFFR-----G-------------X-PEE--------------
--------ATRNLF----XD----------TTX-QSYDFTG-DYTQ----L--RP-RAGV
-------GN-IDSITL-GFNQLDESINYLFRHSGQX------------------------
---HTEEEKNKQATAMIVCIVMIAEAVRFRYIQGTLA-GTIIGD---------------G
RYRSFNN--------------NGAVARFINTWQDLS
>GW777166
YVKLLEELRSRLASGT------DA-------HG--------IAVTR----P--AT-NVPI
-----RE----RFVQVQL-----ENS-G---GN---V-I----TVIINTVNVYVVGY-HV
--------GTASTLNPNILYLD-----D-------------I-PKE--------------
--------ALLQAF----PHPP--------YNH-EPLCFAG-NYDS----L---------
-------PS-RETHEL-GHGALNDAIRNLYYR----------------------------
---------QSEPSALLVIIQMVAEAVRIRYIEHLIL-RNMLGD----------------
-NLNFIP--------------DPRAISMENSWWRLS
>EH756893
YVRLLEELRSRLASGT------DV-------HG--------FAVTR----P--PM-NVPL
-----QE----RFVQVQL-----QNG-F---EHV--M-I----TVIIDTENVYVVGY-LN
--------GTT------LYYLE-----G-------------I-TRGE-------------
--------ELLRAF----PEPG--------YTH-SCLNFTG-SYQS----L---------
-------GN-RSACEL-GHGALNVAIENLYRG----------------------------
---------VSQPKALLVIVQMVAEAVRIKYIEYLIA-RNMEVC----------------
--QNFIP--------------DNRAILLENNWSALS
>EL455240
YARLLTELRSRLRSGT------DV-------HG--------INVTR----P--ST-DIPL
-----KE----RYIQVQL-----ENG-G---GE---I-I----TVIINTENVYVVGY-VT
--------RSNSTLHPTLHYLR-----D-------------N-ETNEA------------
--------LLLQAF----PNTQ--------YAH-DRLRFAG-NYDS----L---------
-------PS-RKTTPL-GHGALNIAIQNLYIGK---------------------------
---------SHQDTAVLVIIQMVAEAVRIWYIESVIL-RQMDEK----------------
YSENFLP--------------DVCFRSIENKWSDLS
>XM002459548
YKSFIASVRAGLVSTAAEAAGTNTS------GG--------IPVLV----S--ED-DPLA
-----LE----ASLNITL-----VNK-A---GR---S-V----SLKMDISGAYIAGY-E-
```

-------AGNF--------------SCL-------------V-KRSG-------------

------------------SG--H------TLSS-VTCYRSSYPWG-------------GP

PTVDVAVPE-V--SAW-RVEDLDEAISSLFLFP---------------------------

---TGNATKEDLSRGLAACDVMVATAATFPYVERRMS-AGMWD-----------------

-GNGVSN--------------DGSLRGLQESWPDLS

>CA078531

YKSFIASVRAGLVSTAEST---NG-------SG--------IPVLV----S--ED-DPLA

-----LE----AFLNVTL-----VNK-A---GR---S-V----SLKMDVSGAYFAGY-E-

-------AGNY--------------SCL-------------L-KRSG-------------

------------------SG--R------TVSS-VTCYRNLYPWG-------------GP

PTVTVDDDP-AAXAAW-RVEDLDEAISSLFLFP---------------------------

---TGNATKEDLSRGLAACDVMIASAATFPYVERRMS-AGMWD-----------------

-GNGVSN--------------DGSLRGLQESWPELS

>AY105813

YKSFIASVRAGLVSKA----GGNS-------SG--------IPVLV----D--ED-DPLA

-----LE----SFLNITL-----VNK-A---GR---S-V----SLKMDVSRAYFVAY-V-

-------AGQR--------------SCL-------------L-KR---------------

------------------SG--R------TFSS-AICYYGG-PWGTSSAPVTRPAAAAG-

-----DGED-PGVAAW-RARDLDEAISSLFLFP---------------------------

---TGNATEEELRRGVAACDMMVASAATFPYVERRMS-AGMWD-----------------

-SNGVAN--------------DPSLRGLQARWPWLS

>FP092597

YENFIAAVRAALKKD-------EI-------HG--------IPVLRCN--P-----YPLL

-----SE----RYLFVTL-----TNK-A---WY---S-I----TLKLDVTGAYFTAY-R-

-------AGNY--------------SCD-------------I------------------

----------HKII----SGLS------------VTCKYAR-GTSS--HVL-MEPSSTGS

HPVDHLAED-LEGVRW-GTNALDEAISSLYRFP---------------------------

---LGMATIREWADGIRTCIMMITNAARFQYIERRMS-AAIRH-----------------

-GNDETE--------------DPSLHSLALRWRDLS

>WP033322798

YSDFLNRLRQRVTGGR------FL-------QGD-------TLRTD----P-------AA

-----RN----DFTIVQL-GAGNEGA-A---AN---E-I----MLLVRNSDLFVVGW-LS

------RSTNR------FTSLE----ND-------------I-PTL--------------

------NDPIDDQR----PP----------AEA-VRARFSG-NYVD----L--ER-RAG-

-------QS-RLEIGL-SPDGVRQSIRDLDRA----------------------------

-----QSTEAQQARALIVLIQVIAEGARFRPIETLYR-DTYIN-----------------

--GARQP--------------DQHLVELENAWQPVS

>NP001159316.1

FEEYIGELREAVRDPTEPP--PLA-----ATHP-------NLYRLR-------QT-TRRE

-----PR----QWLHLRM-----VAR-----NA---E-I----IVWIRTDNLYVVGF-EQ

-------VRGA------RYEFGVDPAND-------------AKEKNKKKKDKEKKAKKKN

KKEDQEQQERRPLI----SG------------A-TFLGFNG-GYGD----L--RQLQTGP

DP-----ER-RTVPDL-GREPLVEAIQGLAAYT---------------------------

-----PAAPGLVRDWLRTIVVTVSESLRLHNVCRHIA-RLLLGEYTR--------GWL--

---------------------DQSLVDCIRDWGDVS

>WP037761173

YNSVISQIRARVSNGS------VR-------DG--------ILRQR----P-------AN

-----VP----DYFAVHF-----QSGVH---TP---Q-L----SLVFNAANLYVVGY-YN

------HATNT------FVRMG-------------------AGPAN--------------

------------------PV-------GAAHVR-NDFLRQG-DYGY----L--ER-VGH-

-------FD-RADQTL-GMGALSAALSTLSNGRPLG------------------------

--SVAATQTAEQARAITTMIQMFAEGARFDFISSNIA-QWTRDDRTYTAGSFVQVRGDGG

TAVELAP------------TQPVNAIDFENVWAQLS

>AL939130

YRSVVQQLRRGAGHLI---------------HG--------NNIYE----TMGQG-----

----QNA----GLVALNL-----YNADW---VH---Q-T----TLYFNASNLYLVGF-KS

-------RTGQ------AYLFS-----D-------------A-SANA-------------

------REEVGREV----RG----------APV-TTLPFAG-SYTS----L--VNTLPG-

-------AE-TEPTTI-GIYAIQTNMEALARTPNPA------------------------

--AATGAYRGSIARAMLIMIGAFAEAARFPMFRDHFE-AAFRR----------------F

ANPSVVV--------------TPTMQALRTAWGQMS

>FN554889

YWNMVAAMHRVTG------------------HD------FFRDTLD----E-----TTTN

-----GN----ALFQVSV----HRYG-----TY-----V---GALYFWTNDLYLAGFYQA

------GEGGG------HYAFN-----E---------------PRR--------------

-------ARFNELL---------------RIQS-TALPWNG-SYTD----F--SG-NAG-

-----DQGS-RSNLQI-NGPRLDNALQQLGRAGSHL------------------------

---QSQNGRAVLSQALVMIIQATSEAARFGRIFDNIR-TNIRD----------------Y

HTGGAQM--------------GAENVNLQQNWGTIS

>YP005709811

YRDLLARIGNAVSHPI--------------VNG--------IGAIP----E--NT-----

----ATN----ARVDIEV-----LTS-E---GN---A-V----TFHMLRRNMYIQAF---

------TLSGT------TYITS-----D-------------A------------------

--------FGRGLI----SG--------------AGLPFSS-DYAS----L--GAHNTG-

-----NISD-PTLFTV-GGASLDTLLCNLFHAV---------------------------

--ENTQNFNRSAARALYAAAILFSEATRFSPSLGRNIAQAIEN-----------------

-GHPYNL--------------TNADSEMIRNWGQLS

>AAWU01015132

YLTFIDSLRGDLSQPN------VA-------HGG-------IRVTK----T---------

-----DN----GITKVIL-----KSG-----TT---Q-I----PFIYRNSDLYIVGF---

------VVGSV------FYADN-----DVFKWISPRETVGKL-PAK--------------

-------HAWRSVLG--------------DVTV-DTLSVPL-SYNN----I-----LQG-

----------RSKVPI---NKMGDSLKKLTEV----------------------------

--GDRNKKSAIVKEHLVPFVVAFSEAIRFTVVARAVR-DAFVKNGGEL-----------D

MKTNVLKLAGAGGPDQRKST-RYDITWLTLNWGSLS

>AAGE02007824

YLGFIQNLISALTESD------EV-------YGN-------IKVTK----R---------

-----KD----GITKVTL-----KDSST---NS---Q-V----GFIYRNRDLYIVGL---

------VVGTT------FYIDK-----EVYDGLG-----KKI-PTT--------------

---------WKGVIN--------------AANT-VSLSNPF-SYNQ----I--LP-SGE-

----------GTVLQI---GKLGDSLTHLTKI----------------------------

--GDTSKRSQIMTQHLGPFVVAFSEAIRFPVVAREIQ-GAIRKATGTV-----------S

LSKNLVRPLSLGHPSERTSNHVFTMYSLLLNWSKLS

>AAGE02007

YLNFIQSLTAAFTESN------EA-------YGG-------VKVTK----R---------

-----TE----GITKVTI-----KDSKA---NT---E-V----GFIYRNRDLYIVGF---

------VVGNT------FYIDK-----EVYNDLK-----NQI-PDK--------------

---------WKKVIN--------------AANT-VQLSNSF-NYID----I--MV-EKG-

----------ATKFQM---VKLSDSLKQLTLV----------------------------

--GDTSKRFDIMRNHLSPFVVLFSEAIRFPVLAREVQ-SELLKPDGSV-----------S

LSMNIVPKLGLGHPSDR--NEVITLFTALTKWGKIS

>AAGE02013700

YIKFIEDLTTTLTEAD------EA-------YDG-------IRVSK----R---------

-----TS----GYTIVII-----K--HG---ET---E-V----PLVYRIRDLYIIGV---

------IVGKV------CYMDH-----KAYAEVN-----NMN-ELN--------------

-----------KVIF--------------PAKA-ESLTREF-SYHF----I--MP-KGE-

----------NTEIRF---EELEFYLQKLTQI----------------------------

--GDKMKRIEVMQKYLGPFVILFSEAVRFPAIAILAQ-QAFRSDDGKI-----------K

LSEDVAPPEVV------LNDNVIKIHTLLLIWGRLS

>EDX20776

YQLLTSTIRSRAEDAQ-------------GFFGGDSRQPGEMAYLP---LQV-QV-----

-----------RDVFIEL-----------Y--------I----ELRLQDAGMRIVGF-RN

-----AFESGEAPSEECVRHVQ-----DSI-----------A------------------

-----------------PPG---------IRRT-EVLPFGG-SRSD----L--ES-AAA-

-------VR-RLGIFL-GRRPLGIAVTWLHR-----------------------------

-----NRDPKCTAHGMLVLSEMICEAARYPALADVMS-----------------------

-----------------------------RIWMTGG

>AGDC01002112

YRSLTSTIRAKADDAQ-------------GFFGGAGRKEGEMPHLP---LHI-KL-----

-----------RGLLIEL-----------F--------I----ELRPRNTSLRIAGF-RN

-----LVENGQAPPEASVHRVR-----DAV-----------A------------------

-----------------PQG---------VDRT-ETLPFGG-DRAD----L--ET-AAA-

-------VR-RAGIML-GRSHLSLAVIRLHR-----------------------------

-----NHDPRSTAYGMLVLSEMLCEAARYPALADAMS-----------------------

-----------------------------RLWTTGG

>EDX25168

YRLLASSIRSRAALAQ-------------CLPGRAGNEEVSARYLP---VRI-QL-----

-----------RGLFIEL-----------F--------I----ELRTQNSVLRITGF-RN

-----TFENWQAPPEGRFRHVR-----DAV-----------A------------------

-----------------PPG---------VHHA-EALSFEG-EVPA----L--EA-AAE-

-------VG-RTGLSL-GRRPMMKAVMWLHR-----------------------------

-----NTDPKCTALGMLVLGEMLCEAASSPVLAQEMS-----------------------

-----------------------------RIWMTGG

>CP003222

YNSNIQNIRDAFRGNA------N------------------VPNVL-------QVKVKIN

-----EA-SCLLYLNAAF-------------ES---R-A----CIPAPNASLYVIGF-KG

-------GDGR------DYLFN-------------------IEPFPN-------------

------------------PELK-------GVNS-VSLPVDG-SCRS----L--GH-----

----------ASSLPIINKAELETLVRTISNF----------------------------

---KGTIISSALSSALTKIIIITCEASRFKSVETDVY-NMLRTSTPFD------------

---------------------ASTRYDMIKRWNTTL

>AFRP01002228

VQRLICSMRERFADPDPTP---------VGPHGFTRRL---IGTGD------------EG

-----TR----NWGLVGV---GHLNYLD---AD---E-V----QLVVDPDDLYIRGFYR-

------RSNNT------LYHFR-----G-------------A------------------

-------GVPDELL----RG----------ANR-VQLGFPE-NYRD----L--AT-----

-------------ITV-DQANIRGAVDSLLHN----------------------------

---SQTGVGSPLQSALELMAVTLAETARNRMINHEVY-NALRQDGAWH------------

---------------------VGAHADVITSWNHMG

Bayesian Inference Figure 2

(EF441598:0.1572734,AF500192:0.2075382,((ZP02062680:0.2549178,(WP034895486:0.2609692,ZP06230219:0.530126):0.1022974):0.1010112,(WP029217990:0.636119,((WP015197802:0.3165974,KIE12545:0.4433638):0.2993933,(AAWU01015132:0.2532313,(AAGE02007824:0.1229897,(AAGE02007:0.1572478,AAGE02013700:0.4676993):0.06084838):0.1688612):0.5064007):0.1877391,(((EEP74218:0.6246986,(((((((((((M77122:0.002442648,EU725161:0.006102643,BT037982:0.006249708,DQ147068:0.002490463):0.0717053,(DQ147051:0.006961918,L26305:0.01233524):0.07794913):0.03707179,Fl902192:0.07565065):0.1564678,(EMT05877:0.10899,XP004975022:0.1118915):0.05694146):0.4146894,((AB051107:0.1247967,(D13795:0.05125172,M62905:0.02008173):0.1029646):0.08560172,ADDN01000393:0.1295216):0.2454883):0.1322524,(((DAA51819:0.00230025,XP008665567:0.002451016):0.4207093,'NP001159316.1':0.6415057):0.173071,((AAX95110:0.1675156,EEE51701:0.171308):0.4479097,Q00531:0.5042111):0.2643989):0.1417685):0.1064183,ABXC01000035:0.5094584):0.1163825,JOWA01000098:0.5129481):0.0819585,(AARH01008880:0.1986276,AGQN01263163:0.2339956):0.2199107):0.0816187,(P84786:0.3333365,AF289116:0.3200164):0.2258956,ACYX02098007:0.2209179,(FR617512:0.2042792,FR606563:0.2330737):0.07257735,((((AFRG01000322:0.04606134,(AFSE01000013:0.04279912,AFRB01000334:0.0165006,(ADFL02000265:0.01970343,AFRF01000004:0.09029904):0.04431858):0.02925616):0.03049635,AFRE01000036:0.03094147):0.3025765,(EJP64846:0.1412242,JPHH02000315:0.06607079):0.08149531):0.05896687,(JMQE01001167:0.2573142,KIJ38555:0.3702156):0.07008009):0.1088148,'HS077718.1':0.3347173,JPUM01000046:0.6204414):0.08769955,JYCQ01000009:0.3414304):0.08810409,(CACC01024922:0.06932143,(CACC0102492:0.06578549,CACC01008026:0.1176928):0.05601078):0.436268,(ADOT01000134:0.4967738,((((DBL01002728:0.0161597,AMZD01000108:0.1136308):0.06784287,JRVE01000053:0.2557418):0.07969744,AOSW01007610:0.13522):0.06417833,AORF01001931:0.2691707):0.4577401):0.07652227,(AEVU01000543:0.1216943,EJP61635:0.02830872):0.4153464,(AQGS01000454:0.5416077,((EDX20776:0.1326875,EDX25168:0.2655527):0.0658845,AGDC01002112:0.1379887):0.7577334):0.1643734):0.1143907,((((((((((L12243:0.3843311,JPQF01109211:0.2041374):0.02957653,(((AGQN01148610:0.04471699,AGQN01137446:0.01270096):0.02654182,AGQN01137447:0.03768705):0.0591192,AGQN01213101:0.08142137):0.4433103):0.1222436,(AY069946:0.09437654,BABX01103831:0.08035363):0.3444457):0.1229693,(EL690296:0.06365666,ADF45510:0.1311725):0.03914307):0.04560314,((AF256085:0.03434386,AF256084:0.02409201):0.08033367,U78041:0.1867328):0.3152332,AJ537497:0.5738623):0.03665021,((M98344:0.08655186,EU008736:0.07956224):0.2093525,AF508915:0.3389128):0.09034974,AY039801:0.2027897,(DQ683359:0.3662408,(X52908:0.2080582,(((XM002459548:0.05433495,CA078531:0.02532897):0.06878213,AY105813:0.08549342):0.4273857,FP092597:0.1543443):0.4850161):0.1306462):0.05546631):0.09871691,(AF249280:0.1445902,AF213983:0.3016243):0.1839177,(((((X62372:0.147019,X57682:0.08481575):0.1159643,(AY669811:0.007386973,TKU25675:0.03377403):0.1106396):0.07057531,AGCB01010835:0.2487693):0.05652456,EU309692:0.1448717):0.1471061,DQ672577:0.3065075):0.08592991,(ACYM01011001:0.2138912,ACHR01003818:0.4563517):0.08256154):0.04936279,(ALYE01008201:0.510084,CAJ38823:0.3977673):0.1326001):0.05606947,((P85101:0.4214711,(AF445416:0.1637413,ABI64066:0.07860575):0.1470844):0.1024911,((D10227:0.209569,AY148091:0.3284198):0.2028173,(AB070925:0.11439,AAB67746:0.3409442):0.2872705,((((AY547315:0.1448082,EU839992:0.156699):0.2056847,AAB96824:0.332852):0.0664972,(AAS67266:0.1716071,(AAK28323:0.0802294,ABJ90432:0.07991755):0.228392):0.1811436):0.0536185,((AM748700:0.03586322,(X59260:0.0627823,AF219236:0.1223534):0.04378211):0.1871076,ADB83313:0.2453459):0.2363663):0.2162):0.05849359):0.1826963):0.07181559,(GW777166:0.1356747,EH756893:0.1883038,EL455240:0.24271):0.3700963):0.2105104):0.07742442,(WP033322798:0.6638547,WP037761173:0.6113422):0.1218101):0.1319887,(AL939130:0.7047102,FN554889:0.6198619):0.1919069):0.09754736,(YP005709811:0.8204234,AFRP01002228:0.820693):0.1593677):0.09869219,CP003222:0.933879):0.3247458):0.1268979);

Maximum Likelihood Figure 2

(XM002459548:0.06630254,CA078531:0.02818049,(AY105813:0.10364069,(FP092597:0.18218521,(X52908:0.26616350,(DQ683359:0.47776815,(AY039801:0.25203589,(((ACHR01003818:0.61438941,ACYM01011001:0.25557543)46:0.10398802,((AF249280:0.18534267,AF213983:0.38293367)99:0.23070899,((DQ672577:0.39317043,(EU309692:0.18434544,(AGCB01010835:0.32303916,((AY669811:0.00528270,TKU25675:0.04090020)100:0.13821859,(X62372:0.18505172,X57682:0.10537773)100:0.14486629)94:0.08242560)63:0.06318835)98:0.18400523)39:0.11690604,((ALYE01008201:0.67661357,CAJ38823:0.51710441)33:0.17493363,((((ADB83313:0.31145053,(AM748700:0.03965839,(X59260:0.07554709,AF219236:0.14999611)98:0.05438292)100:0.23682974)100:0.29889999,((AAS67266:0.21449943,(AAK28323:0.09710547,ABJ90432:0.09828165)100:0.29409188)97:0.23350519,(AAB96824:0.44025793,(AY547315:0.17848911,EU839992:0.19630378)100:0.25667882)73:0.07849315)59:0.06634989)98:0.32160449,((P85101:0.54792286,(AF445416:0.20606043,ABI64066:0.09390975)97:0.18834199)65:0.12477198,((AB070925:0.14215064,AAB67746:0.43631952)100:0.36137529,(D10227:0.25616658,AY148091:0.43520697)99:0.27019027)40:0.07515383)29:0.05526601)90:0.21061369,((EH756893:0.22983612,(GW777166:0.17934352,EL455240:0.31057215)46:0.03965901)100:0.45082787

,((WP033322798:0.88203217,WP037761173:0.83324429)40:0.14742019,((((((EF441598:0.1996310
5,AF500192:0.25188533)95:0.14919059,(ZP02062680:0.32499318,(WP034895486:0.32782635,ZP062
30219:0.71503684)60:0.13172878)57:0.13464665)93:0.42365442,((AAWU01015132:0.30343531,(AA
GE02007824:0.15340076,(AAGE02013700:0.60505758,AAGE02007:0.19851085)63:0.06855421)90:0.2
3973457)100:0.71348061,(WP015197802:0.42356450,KIE12545:0.61293930)98:0.45827591)62:0.25
712851)21:0.11360756,(CP003222:1.48689933,WP029217990:0.91421351)19:0.05439409)14:0.1162
6003,(AFRP01002228:1.18357598,YP005709811:1.22324232)16:0.18869635)19:0.12745057,(FN5548
89:0.88395027,AL939130:0.99541170)38:0.22247645)15:0.18529011,(EEP74218:0.88545341,(ADOT
01000134:0.75231261,(((CACC01024922:0.07623424,(CACC0102492:0.07880857,CACC01008026:0.14
734413)71:0.07414969)100:0.64748235,((AEVU01000543:0.16061174,EJP61635:0.02426226)100:0.
52861460,(AQGS01000454:0.73124454,(EDX20776:0.16725631,(AGDC01002112:0.21426675,EDX25168
:0.35690467)35:0.00906056)100:1.04004946)24:0.18238688)10:0.04057709)6:0.10305477,(((AOR
F01001931:0.28028421,(AOSW01007610:0.17108164,(JRVE01000053:0.33233146,(DBL01002728:0.01
485043,AMZD01000108:0.14605056)69:0.08107194)94:0.10630565)86:0.13278727)100:0.63484678,
(JOWA01000098:0.68556246,(ABXC01000035:0.71517785,((NP001159316.1:0.88120737,(DAA51819:0
.00000000,XP008665567:0.00000000)100:0.53707927)73:0.28723403,(((EMT05877:0.09445841,(XP
004975022:0.14057771,(F1902192:0.09245785,((DQ147051:0.00505669,L26305:0.01331398)100:0.
09727886,(EU725161:0.00458954,(M77122:0.00000022,(BT037982:0.00458421,DQ147068:0.0000000
0)18:0.00000028)37:0.00000022)100:0.08543233)62:0.04163075)98:0.23303123)32:0.05493644)1
00:0.60855894,(ADDN01000393:0.17130600,(AB051107:0.15788262,(D13795:0.06263849,M62905:0.
02138280)100:0.12842866)78:0.09498236)100:0.30836626)72:0.19790241,(Q00531:0.61557209,(A
AX95110:0.20812815,EEE51701:0.22328290)100:0.65974781)91:0.38665730)31:0.09733110)31:0.0
8165464)35:0.13106647)8:0.04774102)8:0.09715449,(JYCQ01000009:0.47733392,(JPUM01000046:0
.86924606,((P84786:0.43723492,AF289116:0.40579158)97:0.29463773,((FR617512:0.26347632,FR
606563:0.30666766)71:0.07835314,(ACYX02098007:0.27909144,((AARH01008880:0.25829635,AGQN0
1263163:0.29072927)99:0.35927585,(HS077718.1:0.47692942,((JMQE01001167:0.30906262,KIJ385
55:0.50033871)31:0.08600589,((AFRE01000036:0.03046768,(AFRG01000322:0.05518724,((ADFL020
00265:0.02098372,AFRF01000004:0.11066052)58:0.05380483,(AFSE01000013:0.05251150,AFRB0100
0334:0.01942258)22:0.00000090)34:0.03491190)44:0.03708112)99:0.38863749,(EJP64846:0.1832
2844,JPHH02000315:0.07438358)71:0.09737340)33:0.06909406)23:0.11889776)16:0.04628223)8:0
.02873774)8:0.04667405)6:0.03930223)9:0.08360767)22:0.06240544)14:0.07557165)1:0.0694799
4)3:0.02761696)13:0.15267382)4:0.04764388)13:0.08216494)56:0.26698773)21:0.10385836)15:0
.05491643)4:0.04857503)2:0.04120827)1:0.03180715)7:0.15893894,((AF508915:0.45238035,(M98
344:0.11149727,EU008736:0.09036011)97:0.26945102)47:0.12310905,((U78041:0.23356760,(AF25
6085:0.04117048,AF256084:0.02639512)90:0.09628888)100:0.44559829,(((AY069946:0.12068217,
BABX01103831:0.09503966)100:0.44317945,((AGQN01213101:0.09842048,(AGQN01137447:0.0463276
1,(AGQN01148610:0.05334923,AGQN01137446:0.01337809)94:0.02958262)88:0.07219227)100:0.590
45225,(JPQF01109211:0.26681063,L12243:0.51869004)51:0.00009037)67:0.16305156)37:0.151441
39,(AJ537497:0.78323031,(EL690296:0.07850852,ADF45510:0.17487632)46:0.03354975)4:0.01880
102)2:0.03351869)3:0.03980909)0:0.01714634)5:0.02766428)19:0.06164821)57:0.16013116)100:
0.65284096)100:0.58553898)97:0.08470069);

Sequence alignment Figure 4

>Xanthomonas_cassavae

YVDSLNVIRSAIGTPLQTI----------SSGGTSLLMIDSGSGDN--LFAVDVRGID--

------------PEEGRFNNLRLIVERNNLYVTGFVNR--------TNNVFYRFADFSHV

TFPGTTA------------------VTLSGDSSYTTLQRVA---GISRTGMQINRHSLTT

SYLDLMSHSGT-----SLTQSVARAMLRFVTVTAEALRFRQIQRGFRTTLDDLSGRSYV-

-------------------MTAEDVDLTLNWGRLS

>Flavobacterium_columnare

YVSSLNSIRTEISTPLEHI----------SQGTTSVSVINHTPPGS--YFAVDIRGLD--

------------VYQARFDHLRLIIEQNNLYVAGFVNT--------ATNTFYRFSDFTHI

SVPGVTT------------------VSMTTDSSYTTLQRVA---ALERSGMQISRHSLVS

SYLALMEFSGN-----TMTRDASRAVLRFVTVTAEALRFRQIQREFRQALSETAPVYT--

-------------------MTPGDVDLTLNWGRIS

>Streptomyces_coelicolor

YVDSLNKIRERIGIRLPNLTT------SQGNMSISVFVLSPSASNI--GIIVTLQGID--

-----------YDDPNTSVPIRLVLSPENLYLAGFI----------QGNTFYRFRDRQNT

VLPSDIHVQI---------------VDLTTGSDYTELERVG---DVRRDRLQINRHSLVT

SYRDLNQFSGT-----ALNQASARAMLRFITVLPEALRFRQIQRNFRPVLSQTASNSHYI

-------------------MSPSNISLTLNWGGLS

>Streptomyces_scabie

YVKSLKDIRGNIGVPLTNI----------GGDQARIFMLPPQREGGMDGIIIAIRGVD--

-----------FYNDEEVAPVNFVLDPTNLYISGFI----------VNNIYYRFSDTSGI

NAPGVIGT-----------------IQINQESSYTSLQRVA---NMQRSDMIINRATLTD

GYSQLTRFSGG-----NINQETARALLRYITVIPEALRFRQIQRNFRPALDVAATQYV--

-------------------MSNPDISLTLNWGRLS

>Streptomyces_lysosuperificus

YVQSLGAIRAAMGDAMSLT---------NIPGNKILYQLRPDASNIVEGVTIEIIGVG--

-----------RNNSPSNRDVRFVINPSDLYLTGFI----------VGRIFYRFSDFSDT

ASGRVQVNAP--------RHLVDFTIDMTVDSSYLSLARSAG-VSADRTDLSIDRYSLMK

GYRDLINHVSST---RTINGAEARALLSYATVLSEAVRFRSIQGNFASTALGDDAFTPYR

-------------------LSLEDSNRTTRWDRLS

>Streptomyces_spI

YVSSLNVIRRALGDPIN------------SPYVNDVLQLRSRSREPVDGVEIRIRGTQEY

DN---TPPPDPSQPDPSQPDITFILNPSDLYIAGFVAPGPNS----VGRIFHRFREYSPG

TNANGIPNLV-------VNANATSVVTINVAVQYSIISIAG--VLSDRTNLSINRHSITS

GYTSLMLRNVNS---TLLDSGEANAILIYATIISEALRYRNIQGIFAGGALGVSGGTFR-

-------------------LNAQQSALTIRWSRLS

>Streptomyces_spII

YAKSISDFRRGIGTEL--------------TRIGNVYETSPVGDLI--------------

---------TVSLSNDRGRQIRLVLRPRDLYVIGYE----------AYGIFYRFNDPEYQ

SLAL--------------PYAATTQLPYNMYSTYDSLSSFG---DIQLSDVNVSRASVYG

AIDTLSRFGDGS----VNANSLSRALYPLIIAVSEGARFRPVSSAIVTALGSSTAMTLGR

-----------------------NTGLVRNWQQIS

>Micromonospora_sp

YFDKLKKQILSLLDNGIILN-------LNNSGGTSYFAINNTQFN--SFVIPFSVE----

------------RDNQSINIDLVFRSSDFYLQGFS----------YDTIYYHFSDSTITS

INGQTP------------------RNLNFDSNYNTLIG--------DSNPTISWSGIEQA

FNDLVNYGENP----GNNSVIRGALARVILATAESMRFRQVRENIERTYNQN--------

--------------IQLYWRDYFYPIITNWSRTTS

>Streptomyces_yerevanensis

YFVSLCNNVLTAMLENNIISSVTESTPELNCNNPNMYIINSNYQFN--YFIIKISVP---

------------DDNGHWKLIDLVFRSNDFYLSGFIYRSN------NVDIFYHFSDVQIE

RNENGQTEFDN----MISGRNNLEYYNLRFNSNYTTLIG--------DSNPTITWPGIVQ

AFNDLVNYANRPNY-QGNIAVIRGALARVILATAESIRFREVRNNLSQSNN---------

---------------TTFYWRTNFLDTITNWNRIS

>Streptomyces_olindensis

YFSDLCNAINFLQNNDNIRLINPE-TIGTTERYYETYFINSNNNDN-KYFVMPI------

-------------NIENDRRISLIFRSSDFYLQGFIGHPTSN--NQTLNVYYYFNDSTIT

NVSGTTSS-----------------QRMSFGSNYTGSNG-----LRADERAPIRWTGIID

AFHQLANYGENPSF--VDSNTLRSSFLRVILATAESMRFREVRNNIISNHNSDT------

---------------STSNWGWYFNNFLRNWDRIS

>Streptomyces_xinghaiensis

-----------------------------FYLQGFIGHPTSN--NQTLNVYYYFNDSTIT

NVSGTTSS-----------------QRMSFGSNYTGSNG-----LRADERAPIRWTGIID

AFHQLANYGENPSF--VDSNTLRSSFLRVILATAESMRFREVRNNIISNHNSDT------

---------------STSNWGWYFNNFLRNWDRIS

>Corynebacterium_ulcerans

-----------------------------LYLQGFILREDNGRQNSADGTYYYFSDSTYR

TNLGINHLTN---------------VNLNFGGSYTNLIA--------DGDLNISSQSLFS

SFRFLASFNNSN----QQRINISSNLMRIILATSESLRFNNIISTIQRNN----------

---------------NPISWRSYFSNLLTNWGSMS

>Escherichia_coliII

-----------------------------LYINGFITTT-------DVKRYYYFNDARIT

SLPPSFKAIA---------------TNLGYASNYNYLVG--------SDNFEISNYTISD

AIAKLQKVTLNT----MFEQEVKKSLAIASLISTESLRFFSVRNAINKILNAE-------

---------------ETKHWTADFKQIVTNWDTYS

>Escherichia_coliI

-----------------------------LYIDGIVAPIPTG----GGDRYYYFNDSVIT

SIPNTTA------------------IGFGFPGNYNNLIG--------SQQLIISRNSLEN

AFYEIIKFNG------VSNDRVKKALVQIIFATSEAMRFFNFAYLTRLIWIEG-------

---------------GYINWQTQIRPDATRWGDIS

>Rickettsiella_grylli

-------------YLETGKRAVLILNPEDLELQGFIQLDSND----TQFKYVYFSNAKIK

NIGTTYV------------------ENLYYTNYYSDLIG--------DAEFIVTRDSMAH

AFNSIANYSGNP-------LIMKKHLVAAMLVTTESFRFFKMADRVGQIWNYE-------

---------------AGYDWQIQKKLYLNNWGKWS

>Erwinia_typographi

------------IYLETGKRAVLILNPEDLELQGFIQLDSND----TQFKYVYFSNAKIK

NIGTTYV------------------ENLYYTNYYSDLIG--------DAEFIVTRDSMAH

AFNSIANYSGNP-------LIMKKHLVAAMLVTTESFRFFKMADRVGQIWNYE-------

---------------AGYDWQIQKKLYLNNWGKWS

>Burkholderia_sp

-----------------------------LYIQGFLD---------KNNIYHYFTKADYK

TYPGAKDN-----------------LSLNYNGDYRSLTA-------GLSEVKLTYNGFNK

MITNLQTPAQ------FSELVVRESLINAILLTAEAFRFANVLYDLDAIINQH-------

---------------QTLNWFTIKDDYLMNWSQWS

>Burkholderia_pyrrocinia 025250933

-----------------------------LYVAGMIN---------NNNEYFHFGDADQQ

FRNINGFATA---------------GILYRSGNYNHLIG--------NGDLQINWAAISN

MYQSLLNFSSSS----NSSGGQNKNLVILITLISESLRFYRITSLMAGALEEDS------

--------------FNSINWGERIRPTVTNWGGIT

>Culex_quinquefasciatus

YIRAVSSIRNSQTELVNLNI------PGSSTNPPQIRRTRNQADNV--YFTVQV------

--------------EFEGNNIGFVFRRRDLYLQGYINS--------RNSTYYYFREATVT

SVRQANSQ-----------------TQLSATENYNSLLGGE------FSRADFNLDELRQ

SFRNLVNNNPS-----RNSQSINTALVRFAVAISEAVRFNEVGRNVARLFRNPS------

---------------ARINFEETHDTVLSNWSRYS

```
>Culex_pipiens
YIRALRDLINTQSQIVTVD--------GLLETGGDIEVPATYNDSN--YFIVQV------
--------------EFDNQSIGFVIRRQNLYVDGFIRNYNPDR---RTGTYYYVRNERNT
TPDNRVVSPE------DMAGEVSNSIELGYGGRYGDFFAND--YSRGRQAQIFGLEDLRQ
DFRNLVNANPNS-------QGVQNAFAHFVIIISEAMRLDPVYDRVVNLFQNPD------
---------------ARIDFRAIHNEYLSRWSDHS
>Culex_torrentium
YWNMVAAMHRVTGHDF----------------FRDTLDETTTNGNA--LFQVSV------
-------------HRYGTYVGALYFWTNDLYLAGFYQAG-------EGGGHYAFNEPRRA
RFNELLRIQS---------------TALPWNGSYTDFSGNAG-DQGSRSNLQINGPRLDN
ALQQLGRAGSHLQS-QNGRAVLSQALVMIIQATSEAARFGRIFDNIRTNIRDYHTGGAQ-
-------------------MGAENVNLQQNWGTIS
>Culex_molestus
YRSVVQQLRRGAGHLI---------------HGNNIYETMGQGQNA-GLVALNL------
------------YNADWVHQTTLYFNASNLYLVGFKS---------RTGQAYLFSDASAN
AREEVGR-----------EVRGAPVTTLPFAGSYTSLVNTL--PGAETEPTTIGIYAIQT
NMEALARTPNPAAATGAYRGSIARAMLIMIGAFAEAARFPMFRDHFEAAFRRFANPSVV-
-------------------VTPTMQALRTAWGQMS
>Aedes_albopictusII
YEDLITLIRVQNQLSANTPVTPAP--------------TASDADPA-GYFSVAL------
--------------VAGGRRIELVIRRDDLYLVGWYQADQDR--AGDADVYFRFRHDRSG
SPYD------------FRRTTRTRIVDLTFTGSYLDLGY----PAEKRKALNLGGGALIK
ALQLLS---------GTNGQQPNEALVVTIQMVLEAARFHPLFEHLREHWDEWGAPPPA-
-----------------------LVDLQNNWGDLS
>Aedes_aegyptiI
YNSVISQIRARVSNGS---------------VRDGILRQRPANVPD--YFAVHF------
------------QSGVHTPQLSLVFNAANLYVVGYYNH--------ATNTFVRMGAGPAN
PVGAAHVR-----------------NDFLRQGDYGYLERVG---HFDRADQTLGMGALSA
ALSTLSNGRPLGSVAATQTAEQARAITTMIQMFAEGARFDFISSNIAQWTRDDRTYTAGS
FVQVRG--DGGTAVELAPTQPVNAIDFENVWAQLS
>Aedes_albopictusI
YSDFLNRLRQRVTGGR--------------FLQGDTLRTDPAARND--FTIVQLGAG---
------------NEGAAANEIMLLVRNSDLFVVGWLSR--------STNRFTSLENDIPT
LNDPIDD-----------QRPPAEAVRARFSGNYVDLERRA---GQSRLEIGLSPDGVRQ
```

SIRDLDRAQSTE-------AQQARALIVLIQVIAEGARFRPIETLYRDTYINGARQPDQH

-----------------------LVELENAWQPVS

>Aedes_albopictusIV

YLTFIDSLRGDLSQPN--------------VAHGGIRVTKTDNGIT--KVILKS------

----------------GTTQIPFIYRNSDLYIVGFV----------VGSVFYADNDVFKW

ISPRETVGKLPAKHAWRSVLGDVTVDTLSVPLSYNNI---------LQGRSKVPINKMGD

SLKKLTEVGDRN----KKSAIVKEHLVPFVVAFSEAIRFTVVARAVRDAFVKNGGE-LDM

KTNVLKLAGAGGPD-QRKSTRYDITWLTLNWGSLS

>Aedes_albopictusVII

YLTFIDGLRGDLSQPK--------------VAHGGIRVTKTDNGIT--KVILKS------

----------------GTTQIPFIYRNSDLYIVGFV----------IGSVFYADNDVFKW

ISPRETVGKLPAKHAWRSVLGDVTVDTLSVPLSYNNI---------LQGRSKVPINKMGD

SLKKLTEVGDRN----KKSAIVKEHLVPFVVAFSEAIRFTVVARAVKDAFVKNGGE-LDM

KTNVLKLAGAGGPD-QRKSTRYDITWLTLNWGSLS

>Aedes_albopictusIII

YLTFIDGLRGDLSQPK--------------VAHGGIRVTKTDNGIT--KVILKS------

----------------GSTQIPFIYRNSDLYIVGFV----------VGSVFYADNDVFKW

ISPRETVGKLPAKHAWRSVLGDVSVDTLSVALSYNNI---------LQGRSKVPINKMGD

SLKKLTEVGDRN----KKSGIVKEHLVPFVVAFSEAIRFTVVARAVRDAFVKNGGE-LDM

KTNVLKLAGAGGPD-QRKSTRYDITWLTLNWGSLS

>Aedes_albopictusVI

YLTFINSLRGDLSQPK--------------VAHGGIRVTKTDNGIT--KVILKS------

----------------GSTQIPFIYRNSDLYIVGFV----------VGSVFYADNDVFKW

ISPRETVGKLPAKHAWRSVLGDVSVDTLSVALSYNNI---------LQGRSKVPINKMGD

SLKKLTEVGDRN----KKSGIVKEHLVPFVVAFSEAIRFTVVARAVRDAFVKNGGE-LDM

KTNVLKLAGAGGPD-QRKSTRYDITWLTLNWGSLS

>Aedes_aegyptiIII

YLGFIQNLISALTESD--------------EVYGNIKVTKRKDGIT--KVTLKD------

--------------SSTNSQVGFIYRNRDLYIVGLV----------VGTTFYIDKEVYDG

LGKKIPTT-------WKGVINAANTVSLSNPFSYNQI-------LPSGEGTVLQIGKLGD

SLTHLTKIGDTS----KRSQIMTQHLGPFVVAFSEAIRFPVVAREIQGAIRKATGT-VSL

SKNLVRPLSLGHPSERTSNHVFTMYSLLLNWSKLS

>Aedes_aegyptiII

YLGFIQSLTSALTESG--------------EVYGKIKVTKRNNGIT--KVTIKD------

--------------SASNAQVGFIYRNRDLYIVGLV----------VGNTFYIDKEVYDG

LSKRIPNS-------WKSVINAPNTVALSNPFSYNQI-------LPSGEGTVLQIGKLGN

SLSQLTKIGDTS----KRSQIMTQHLGPFVVAFSEAIRFPVVAREIQGAIRKATGT-VSL

SKNLVKPLSLGHPSERTKNHVFTMYSLLLNWSKLS

>Aedes_albopictusV

YINFIESLTSALTESN--------------EVYGNIKVTKRNDGIT--KVTIKD------

--------------SASNTEVGFIYRNRDLYIVGLV----------VGNTFYIDKEVYDG

HGKKIPNS-------WKNVINAPNTVALSNPFSYNQI-------LPSGESTVLQIGKLGD

SLKQLTNIGDTS----KRSQIMTQHLGPFVVAFSEAIRFPVVAREIQDAIRKATGT-VSL

SKNLVQPLSLGHPSERTDKHVFTMYSLLLNWSKLS

>Calothrix_parietina

YLKFIDSLVTRFSDPN--------------VKFGQIKVTKKDDGIT--KVTIKD------

--------------SSTSTQVGFIYRNRDLYIVGLL----------VDTTFYIEGDAYKK

AKAETLNS-------WKSAIGAKNMVDVS-SFTYANI-------LPYGENTNFELSKLGE

SLTQLVQIGDTK----KRKDIVETHLSPFVVAFAEAIRFPVVKREVQDMIRKPPGTFLKL

SKNIIPMLSLGHPDERTSKQVFSIYSLLLIWGKLS

>Tolypothrix_bouteillei

YLKFIDSLVTRFTDPN--------------VRFGQIKVTRNNDGIT--KVTIKD------

--------------SSTSTQVGFIYRNRDLYIVGLL----------VDTTFYIEGDAYKN

AKAETLNS-------WKSAIGAKNMVDVS-SFTYANI-------LPYGENTNFELSKLGE

SLTQLVQIGDKK----KRKDIVETHLSPFVVAFAEAIRFPVVKREVQDMIRKPPGTFLKL

SKNIVPILSLGHPDQRTPKHVFSIYSLLLSWGKLS

>Spiroplasma_poulsoniiIV

YLKFINSLVSRLTEPN--------------EKYGQIKVTKRDDGVT--KVTIKD------

--------------SSTNTQVGFIYRNRDLYIVGLI----------VDGTFYIDSQVYGE

LGKKIPNA-------WKSVINAQKTVEIR-PFIYGNI-------LPKGEKTELKIAKLGE

SLQQLVQIGDTF----KRIDIMKDHLSPFVVAFAEAIRFPVVAREVQDVIRKPLGT-LKL

SKNLVRMLSLGHPDARTPNHVFSIYSLLLSWGKLS

>Spiroplasma_poulsoniiIII

YLKFINSLVSRLTEPN--------------EKYGQIKVTKRDDGVT--KVTIKD------

--------------SSTNTQVGFIYRNRDLYIVGLI----------VDGTFYIDSQVYGE

LGKKIP--------------NAQKTVEIR-PFIYGNI-------LPKGDKTELKIAKLGE

SLQQLVQIGDTF----KRIDIMKDHLSPFVVAFAEAIRFPVVAREVQDVIRKPLGT-LKL

SKNLVRTLSLGHPDARTPNHVFSIYSLLLSWGKLS

>Spiroplasma_poulsoniiII

YLNFIQSLTAAFTESN--------------EAYGGVKVTKRTEGIT--KVTIKD------

--------------SKANTEVGFIYRNRDLYIVGFV----------VGNTFYIDKEVYND

LKNQIPDK-------WKKVINAANTVQLSNSFNYIDI-------MVEKGATKFQMVKLSD

SLKQLTLVGDTS----KRFDIMRNHLSPFVVLFSEAIRFPVLAREVQSELLKPDGS-VSL

SMNIVPKLGLGHPSDR--NEVITLFTALTKWGKIS

>Spiroplasma_poulsoniiI

YIKFIEDLTTTLTEAD--------------EAYDGIRVSKRTSGYT--IVIIKH------

----------------GETEVPLVYRIRDLYIIGVI----------VGKVCYMDHKAYAE

VNNMNELN---------KVIFPAKAESLTREFSYHFI-------MPKGENTEIRFEELEF

YLQKLTQIGDKM----KRIEVMQKYLGPFVILFSEAVRFPAIAILAQQAFRSDDGK-IKL

SEDVAP------PEVVLNDNVIKIHTLLLIWGRLS

>Spiroplasma_sp

YKNFIAELTTLFTEAN--------------EQYDGIKVSKRASGYT--KVIIKH------

----------------GQTEVPLVYRNRDLYIIGVI----------VGKVCYVDKKAHAE

MNDLQELK---------KVIAPAKDERLSAEFSYQFI-------MPKAEHTEIRFEELEY

YLQKLTLISDKM----KRMEAMT-FLSPFVVLFAEAIRFPVIATLAQRAFRSVDGT-LIL

STDVAP------PEMARNSYVIMIHKLLLNWGTLS

>Spiroplasma_sabaudienseIV

YRDLLARIGNAVSHPI-------------VNGIGAIPENTATNARV--------------

---------DIEVLTSEGNAVTFHMLRRNMYIQAFT----------LSGTTYITSDA---

-----------------FGRGLISGAGLPFSSDYASLGAHNTGNISDPTLFTVGGASLDT

LLCNLFHAVENT---QNFNRSAARALYAAAILFSEATRFSPSLGRNIAQAIENG------

---------------HPYNLTNADSEMIRNWGQLS

>Spiroplasma_sabaudienseIII

YNSNIQNIRDAFRGNANVPNVLQV------------------------KVKINEASC---

---------LLYLNAAFESRACIPAPNASLYVIGFKG---------GDGRDYLFNIEPFP

NP----------------ELKGVNSVSLPVDGSCRSL-------GHASSLPIINKAELET

LVRTISNFKGT-----IISSALSSALTKIIIITCEASRFKSVETDVYNMLRTSTPFDAST

-----------------------RYDMIKRWNTTL

>Spiroplasma_eriocheirisI

VQRLICSMRERFADPDPTPVGPH-------------------------GFTRRLIGTGDE

GTRNWGLVGVGHLNYLDADEVQLVVDPDDLYIRGFYRR--------SNNTLYHFRGAGVP

----------------DELLRGANRVQLGFPENYRDLATIT-----------VDQANIRG

AVDSLLHNSQT-----GVGSPLQSALELMAVTLAETARNRMINHEVYNALRQDGAWHVGA

-----------------------HADVITSWNHMG

>Spiroplasma_eriocheirisII

YQLLTSTIRSRAEDAQ--------------GFFGGDSRQPGEMAYL--PLQVQVRDV---

---------------FIELYIELRLQDAGMRIVGFRNAFESGE-APSEECVRHVQDSIA-

------------------PPGIRRTEVLPFGGSRSDLESAA---AVRRLGIFLGRRPLGI

AVTWLHRNRDPK---C-----TAHGMLVLSEMICEAARYPALADVMSR------------

-----------------------------IWMTGG

>Spiroplasma_sabaudienseI

YRSLTSTIRAKADDAQ--------------GFFGGAGRKEGEMPHL--PLHIKLRGL---

---------------LIELFIELRPRNTSLRIAGFRNLVENGQ-APPEASVHRVRDAVA-

------------------PQGVDRTETLPFGGDRADLETAA---AVRRAGIMLGRSHLSL

AVIRLHRNHDPR---S-----TAYGMLVLSEMLCEAARYPALADAMSR------------

-----------------------------LWTTGG

>Spiroplasma_sabaudienseII

YRLLASSIRSRAALAQ--------------CLPGRAGNEEVSARYL--PVRIQLRGL---

---------------FIELFIELRTQNSVLRITGFRNTFENWQ-APPEGRFRHVRDAVA-

------------------PPGVHHAEALSFEGEVPALEAAA---EVGRTGLSLGRRPMMK

AVMWLHRNTDPK---C-----TALGMLVLGEMLCEAASSPVLAQEMSR------------

-----------------------------IWMTGG

Bayesian Inferences Figure 4

((Xanthomonas_cassavae:0.6408855,((Streptomyces_scabie:0.7944141999999998,Streptomyces_c
oelicolor:0.7665703000000001):0.1791224000000001,(Micromonospora_sp:0.6246067,((Strepto
myces_olindensis:0.7267022999999999,Streptomyces_yerevanensis:0.7431888):0.1894008000000
0004,((Streptomyces_spI:0.1883265999999999,Streptomyces_spII:0.3301588999999998):0.12735
399999999997,Streptomyces_lysosuperificus:0.06280814000000001):1.141549):0.1601056999999
999):0.18693819999999994):0.17320809999999986,Corynebacterium_ulcerans:1.063374999999999
7,(Flavobacterium_columnare:1.076569,Streptomyces_xinghaiensis:0.8384946):0.2875603):0.0
7340660000000065,((((((Spiroplasma_poulsoniiI:0.22165729999999995,Spiroplasma_sp:0.31156
08999999999):0.20490619999999993,(Spiroplasma_poulsoniiII:0.003661877000000091,Spiroplas
ma_poulsoniiIII:0.003513569000000105):0.38953269999999995):0.2622188000000001,Spiroplasm
a_poulsoniiIV:0.4432212):0.12430570000000007,((Spiroplasma_eriocheirisI:0.5666544,(Spiro
plasma_sabaudienseI:0.003729163000000353,Spiroplasma_sabaudienseII:0.003696563999999957
8):0.6335593000000002,Spiroplasma_eriocheirisII:0.7693274999999999):0.14393959999999995,
Spiroplasma_sabaudienseIII:0.5492665000000001,Spiroplasma_sabaudienseIV:0.7605297):0.130
4239000000004):0.2054475,((Calothrix_parietina:0.2035669,Tolypothrix_bouteillei:0.64422
22):0.23128499999999996,((Culex_quinquefasciatus:0.004145777999999822,(Culex_pipiens:0.0
1093481000000184,(Culex_torrentium:0.0043256910000002,Culex_molestus:0.007060270999999
84):0.015439639999999866):0.007216478000000137):0.40215080000000003,((((Aedes_aegyptiI:0
.05963799000000014,Aedes_albopictusI:0.0319436600000004):0.02313289999999979,Aedes_albo
pictusII:0.02833713000000155):0.08385292999999994,((Aedes_albopictusIII:0.0221263500000
00183,Aedes_albopictusVI:0.026418829999999893):0.19083229999999984,(Aedes_albopictusIV:0
.002582448000000705,Aedes_albopictusVII:0.009310945999999982):0.06420090999999983):0.120
38249999999984):0.047248549999999945,Aedes_aegyptiIII:0.18972139999999982,(Aedes_aegypti
II:0.13043130000000014,Aedes_albopictusV:0.1304411000000001):0.4224983):0.18462020000000
012):0.850519):0.2533924000000001):0.23067519999999986,((Rickettsiella_grylli:0.33308249

99999997,(Erwinia_typographi:0.3161166999999998,(Burkholderia_sp:0.3121929999999997,Burk
holderia_pyrrocinia:0.5774568999999998):0.4007271000000001):0.09966162):0.10197039999999
99,(Escherichia_coliII:0.21712450000000016,Escherichia_coliI:0.2173267000000001):0.18570
00000000002):0.3709794999999998):0.0);

Maximum Likelihood Figure 4

((Xanthomonas_cassavae:1.0682038,(Flavobacterium_columnare:2.2395021699999997,(((Strepto
myces_coelicolor:1.2604359199999995,Streptomyces_scabie:1.2926153299999998)[&bs=33]:0.24
450225000000003,((Streptomyces_lysosuperificus:0.029638330000000046,(Streptomyces_spI:0.
2761339500000002,Streptomyces_spII:0.4832000599999997)[&bs=86]:0.22734743000000002)[&bs=
100]:2.0821775299999996,(Micromonospora_sp:0.9966382699999996,(Streptomyces_yerevanensis
:1.18608693,Streptomyces_olindensis:1.1891925900000002)[&bs=62]:0.3140075699999998)[&bs=
39]:0.14316686)[&bs=35]:0.22461790000000015)[&bs=42]:0.18655584999999997,(Streptomyces_x
inghaiensis:1.75127504,Corynebacterium_ulcerans:1.9168879699999999)[&bs=5]:0.01851841999
9999924)[&bs=16]:0.1677572500000002)[&bs=9]:1.3640000000147978E-
5)[&bs=34]:0.10837007999999893,(((Escherichia_coliII:0.3177058100000001,Escherichia_coli
I:0.30059250000000004)[&bs=97]:0.24701959999999978,(Rickettsiella_grylli:0.4736370600000
0017,(Erwinia_typographi:0.4364206799999999,(Burkholderia_sp:0.44822507999999983,Burkhol
deria_pyrrocinia:0.8815233799999995)[&bs=96]:0.6568654899999999)[&bs=39]:0.1465354799999
9983)[&bs=45]:0.14272316000000007)[&bs=91]:0.60432285,((((Culex_quinquefasciatus:5.30000
0003316541E-7,(Culex_pipiens:0.009528200000000098,(Culex_torrentium:3.299999997707914E-
7,Culex_molestus:0.009501430000000255)[&bs=83]:0.019321190000000032)[&bs=72]:0.0096233699
99999909)[&bs=100]:0.5871084799999999,(((Aedes_albopictusII:0.03596727000000044,(Aedes_a
egyptiI:0.07824199000000043,Aedes_albopictusI:0.04004461999999975)[&bs=86]:0.02715984000
000038)[&bs=98]:0.11285744999999991,((Aedes_albopictusIV:2.8000000007466497E-
7,Aedes_albopictusVII:0.009816350000000362)[&bs=99]:0.0805258999999996,(Aedes_albopictus
III:0.027206239999999937,Aedes_albopictusVI:0.03308140000000037)[&bs=97]:0.2641026000000
002)[&bs=94]:0.16920340999999972)[&bs=49]:0.05398679999999967,(Aedes_aegyptiIII:0.241316
99999999956,(Aedes_aegyptiII:0.18956564999999959,Aedes_albopictusV:0.1686070700000002)[&
bs=100]:0.6596825300000004)[&bs=32]:0.07569982999999958)[&bs=65]:0.2523923499999996)[&bs
=100]:1.36816767,(Calothrix_parietina:0.2979312599999999,Tolypothrix_bouteillei:0.975308
3500000002)[&bs=86]:0.32440824999999984)[&bs=59]:0.3987411899999995,(Spiroplasma_poulson
iiIV:0.6400776599999998,(((Spiroplasma_poulsoniiIII:3.400000001541059E-
7,Spiroplasma_poulsoniiII:9.99999993922529E-
9)[&bs=100]:0.5768278099999997,(Spiroplasma_poulsoniiI:0.31871411000000016,Spiroplasma_s
p:0.44721301999999996)[&bs=98]:0.29361767999999966)[&bs=95]:0.4093976699999997,((Spiropl
asma_sabaudienseIV:1.3062889700000007,Spiroplasma_sabaudienseIII:0.8590218600000004)[&bs
=15]:0.1264995399999993,(Spiroplasma_eriocheirisI:0.9470348100000008,(Spiroplasma_erioch
eirisII:1.2808265299999997,(Spiroplasma_sabaudienseI:9.99999993922529E-
9,Spiroplasma_sabaudienseII:3.800000003550963E-
7)[&bs=100]:0.9788998700000002)[&bs=25]:0.12561971999999955)[&bs=41]:0.21691772000000054
)[&bs=18]:0.21435748000000032)[&bs=25]:0.2001107099999997)[&bs=52]:0.32564373000000035)[
&bs=30]:0.35032967999999975)[&bs=34]:0.18546084000000107);
